# Supplementary material for: Invasive acupuncture for gastroparesis after thoracic or abdominal surgery: a systematic review and meta-analysis
Source: BMJ Open. 2023 Jun 26;13(6):e068559. doi: 10.1136/bmjopen-2022-068559 (PMC10410841; doi:10.1136/bmjopen-2022-068559)
Supplement: Supplementary data [file bmjopen-2022-068559supp003.pdf]

Supplemental Table 2 Search strategy

Database	#	Search syntax
PubMed	1	(acupuncture[MeSH Terms]) OR (acupuncture therapy[MeSH Terms]) OR (acupoint[MeSH Terms]) OR (electroacupuncture[MeSH Terms]) OR (electric acupuncture[MeSH Terms]) OR (auriculotherapy[MeSH Terms]) OR (auricular acupuncture[MeSH Terms]) OR (elongated needle[MeSH Terms]) OR (scalp acupuncture[MeSH Terms]) OR (hand acupuncture[MeSH Terms]) OR (manual acupuncture[MeSH Terms]) OR (body acupuncture[MeSH Terms]) OR (warm* acupuncture[MeSH Terms]) OR (warm* needl*[MeSH Terms]) OR (fire needl*[MeSH Terms]) OR (trigger point[MeSH Terms]) OR (meridian[MeSH Terms]) OR (needl*[MeSH Terms]) OR (abdominal acupuncture[MeSH Terms]) OR (float* needl*[MeSH Terms]) OR (embedded acupuncture[MeSH Terms]) OR (intradermal acupuncture[MeSH Terms]) OR (needl*[MeSH Terms]) OR (acupuncture*[Title/Abstract]) OR (acupuncture therapy[Title/Abstract]) OR (acupoint*[Title/Abstract]) OR (electroacupuncture*[Title/Abstract]) OR (electric acupuncture*[Title/Abstract]) OR (auriculotherapy[Title/Abstract]) OR (auricular acupuncture*[Title/Abstract]) OR (elongated needl*[Title/Abstract]) OR (scalp acupuncture*[Title/Abstract]) OR (hand acupuncture*[Title/Abstract]) OR (manual acupuncture*[Title/Abstract]) OR (body acupuncture*[Title/Abstract]) OR (warm* acupuncture*[Title/Abstract]) OR (warm* needl*[Title/Abstract]) OR (fire needl*[Title/Abstract]) OR (trigger point*[Title/Abstract]) OR (meridian*[Title/Abstract]) OR (needl*[Title/Abstract]) OR (abdominal acupuncture*[Title/Abstract]) OR (float* needl*[Title/Abstract]) OR (embedded acupuncture*[Title/Abstract]) OR (intradermal acupuncture*[Title/Abstract]) OR (electro-acupuncture*[Title/Abstract]) OR (micro-puncture*[Title/Abstract]) OR (needl* embedding[Title/Abstract])
	2	(surgery[MeSH Terms]) OR (operation[MeSH Terms]) OR (postoperative[MeSH Terms]) OR (surger*[Title/Abstract]) OR (operation*[Title/Abstract]) OR (postoperati*[Title/Abstract]) OR (postsurgical[Title/Abstract]) OR (excision*[Title/Abstract]) OR (resecti*[Title/Abstract])
	3	(gastroparesis[MeSH Terms]) OR (gastropareses[MeSH Terms]) OR (gastric stasis[MeSH Terms]) OR (gastric stases[MeSH Terms]) OR (stasis, gastric[MeSH Terms]) OR (gastr* paralysis[MeSH Terms]) OR (delay* gastric emptying[MeSH Terms]) OR (stomach palsy[MeSH Terms]) OR (gastr* motility disorder[MeSH Terms]) OR (gastroparesis[Title/Abstract]) OR (stasis, gastric[Title/Abstract]) OR (gastr* paralysis[Title/Abstract]) OR (delay* gastric emptying[Title/Abstract]) OR (stomach palsy[Title/Abstract]) OR (gastr* motility disorder[Title/Abstract]) OR (gastric stasis[Title/Abstract]) OR (gastroparesis syndrome[Title/Abstract]) OR (impaired gastric accommodation[Title/Abstract]) OR (stomach paresis[Title/Abstract])
	4	(Randomized controlled trial) or ((controlled) and (study or design or trial)) or (random*) or (intermethod comparison) or (placebo) or (compar*) or ((singl* or doubl* or trebl* or tripl*) and (blind* or mask*)) or (cross over) or (trial[Title/Abstract])
	5	#1 AND #2 AND #3 AND #4
Embase	1	'acupuncture'/exp OR 'needle'/exp OR 'electroacupuncture'/exp OR 'auricular acupuncture'/exp OR 'acupuncture needle'/exp OR 'scalp acupuncture'/exp OR 'manual acupuncture'/exp OR 'body acupuncture'/exp OR 'warm acupuncture'/exp OR 'meridian'/exp OR 'abdominal acupuncture'/exp OR 'trigger point'/exp OR 'dry needling'/exp OR 'acupuncture*':ti,ab,kw OR 'needl*':ti,ab,kw OR 'electroacupuncture*':ti,ab,kw OR 'auricular acupuncture*':ti,ab,kw OR 'acupuncture* needl*':ti,ab,kw OR 'scalp acupuncture*':ti,ab,kw OR 'manual acupuncture*':ti,ab,kw OR 'body acupuncture*':ti,ab,kw OR 'warm* acupuncture*':ti,ab,kw OR 'meridian*':ti,ab,kw OR 'abdominal acupuncture*':ti,ab,kw OR 'trigger point*':ti,ab,kw OR 'dry needl*':ti,ab,kw OR 'acupuncture* therap*':ti,ab,kw OR 'acupoint*':ti,ab,kw OR 'electric

		acupuncture*':ti,ab,kw OR 'electro-acupuncture*':ti,ab,kw OR 'auricular-acupuncture*':ti,ab,kw OR 'auriculotherap*':ti,ab,kw OR 'elongated needl*':ti,ab,kw OR 'micro-puncture*':ti,ab,kw OR 'hand acupuncture*':ti,ab,kw OR 'warm* needl*':ti,ab,kw OR 'fire needl*':ti,ab,kw OR 'float* needl*':ti,ab,kw OR 'embedd* acupuncture*':ti,ab,kw OR 'needl* embedd*':ti,ab,kw OR 'intradermal acupuncture*':ti,ab,kw
	2	'surgery'/exp OR 'excision'/exp OR 'surger*':ti,ab,kw OR 'excision*':ti,ab,kw OR 'operation*':ti,ab,kw OR 'postoperation*':ti,ab,kw OR 'postsurgical*':ti,ab,kw OR 'postoperative*':ti,ab,kw OR 'resection*':ti,ab,kw
	3	'stomach paresis'/exp OR 'gastric stasis'/exp OR 'stomach emptying'/exp OR 'gastric dysmotility'/exp OR 'stomach paresis':ti,ab,kw OR 'gastric stasis':ti,ab,kw OR 'stomach emptying':ti,ab,kw OR 'gastric dysmotility':ti,ab,kw OR 'gastroparesis':ti,ab,kw OR 'gastropareses':ti,ab,kw OR 'stasis, gastric':ti,ab,kw OR 'gastr* paralysis':ti,ab,kw OR 'gastroparesis syndrome':ti,ab,kw OR 'delay* gastr* empty*':ti,ab,kw OR 'disturbance of gastric emptying':ti,ab,kw OR 'gastr* motility disorder':ti,ab,kw OR 'impaired gastric accommodation':ti,ab,kw OR 'stomach paralysis':ti,ab,kw
	4	'Randomized controlled trial' or 'controlled adj7 (study or design or trial)' or 'random*' or 'intermethod comparison' or 'placebo' or 'compar*' or '(singl* or doubl* or trebl* or tripl*) adj (blind* or mask*)' or '(cross-over or crossover or cross over)' or 'trial':ti,kw,ab
	5	#1 AND #2 AND #3 AND #4
CENTRAL	1	(MeSH descriptor:[Acupuncture OR 'Acupuncture Therapy' OR 'Acupuncture Points' OR 'Needles' OR 'Auriculotherapy' OR 'Acupuncture, Ear' OR 'Electroacupuncture' OR 'Trigger Points' OR 'Meridians' OR 'Dry Needling'] explode all trees) OR (acupuncture* OR needl* OR electroacupuncture* OR 'auricular acupuncture*' OR 'acupuncture* needl*' OR 'scalp acupuncture*' OR 'manual acupuncture*' OR 'body acupuncture*' OR 'warm* acupuncture*' OR meridian* OR 'abdominal acupuncture*' OR 'trigger point*' OR 'dry needl*' OR 'acupuncture* therap*' OR acupoint* OR 'electric acupuncture*' OR electro-acupuncture* OR auricular-acupuncture* OR auriculotherap* OR 'elongated needl*' OR 'micro-puncture*' OR 'hand acupuncture*' OR 'warm* needl*' OR 'fire needl*' OR 'float* needl*' OR 'embedd* acupuncture*' OR 'needl* embedd*' OR 'intradermal acupuncture*'):ti,ab,kw
	2	MeSH descriptor:[General Surgery] explode all trees OR (surger* OR excision* OR operation* OR postoperation* OR postsurgical* OR postoperative* OR resection*):ti,ab,kw
	3	MeSH descriptor:[Gastroparesis] explode all trees OR (gastroparesis OR gastropareses OR gastric stasis OR gastric stases OR 'gastr* paralysis' OR 'delay* gastric emptying' OR 'disturbance of gastric emptying' OR 'weakness of gastric emptying' OR 'weakness of stomach' OR 'stomach palsy' OR 'gastr* motility disorder' OR 'gastroparesis syndrome' OR 'impaired gastric accommodation' OR 'stomach paresis'):ti,ab,kw
	4	#1 AND #2 AND #3
Medline (Ovid) (1946-)	1	exp Acupuncture/ or exp Acupuncture Therapy/ or exp Acupuncture Points/ or exp Needles/ or exp electroacupuncture/ or exp auriculotherapy/ or exp Acupuncture, Ear/ or exp Trigger Points/ or exp Meridians/ or exp dry needling/ OR (acupuncture* OR acupuncture therapy OR acupoint* OR electroacupuncture* OR electric acupuncture* OR auriculotherapy OR auricular acupuncture* OR elongated needl* OR scalp acupuncture* OR hand acupuncture* OR manual acupuncture* OR body acupuncture* OR warm* acupuncture* OR warm* needl* OR fire needl* OR trigger point* OR meridian* OR needl* OR abdominal acupuncture* OR float* needl* OR embedded acupuncture* OR intradermal acupuncture* OR needl* OR electro-acupuncture* OR micro-puncture* OR needl* embedding).ab,kw,ti.
	2	exp General Surgery/ or exp Postoperative Complications/ or exp "Postoperative Nausea and Vomiting"/ or exp Postoperative Period/ or (surger* or operation* or postoperati* or postsurgical

		or postoperati* or excision* or resecti*).ab,kw,ti.
	3	exp Gastroparesis/ or exp Gastrointestinal Motility/ or exp Gastric Emptying/ or (gastroparesis or stasis, gastric or delay* gastric emptying or disturbance of gastric emptying or weakness of gastric emptying or weakness of stomach or stomach palsy or gastr* motility disorder or gastric stasis or gastroparesis syndrome or impaired gastric accommodation or stomach paresis).ab,kw,ti.
	4	((randomised controlled trial or controlled clinical trial or pragmatic clinical trial).pt. or (random* or placebo or trial or groups).ti,ab. or drug therapy.fs.) not (animals not (humans and animals)).sh.
	5	#1 AND #2 AND #3 AND #4
Web of Science	1	TS=(acupuncture* OR acupuncture therapy OR acupoint* OR electroacupuncture* OR electric acupuncture* OR auriculotherapy OR auricular acupuncture* OR elongated needl* OR scalp acupuncture* OR hand acupuncture* OR manual acupuncture* OR body acupuncture* OR warm* acupuncture* OR warm* needl* OR fire needl* OR trigger point* OR meridian* OR needl* OR abdominal acupuncture* OR float* needl* OR embedded acupuncture* OR intradermal acupuncture* OR electro-acupuncture* OR micro-puncture* OR needl* embedding OR dry needl*)
	2	TS=(surger* OR operation* OR postoperati* OR postsurgical OR excision* OR resecti*)
	3	TS=(gastroparesis OR gastroparesis OR gastric stasis OR gastric stases OR stasis, gastric OR stases, gastric OR gastr* paralysis OR delay* gastric emptying OR disturbance of gastric emptying OR weakness of gastric emptying OR weakness of stomach OR stomach palsy OR gastr* motility disorder OR gastroparesis syndrome OR impaired gastric accommodation OR stomach paresis)
	4	ALL=((Randomized controlled trial) or ((controlled) "near/7" (study or design or trial)) or (random*) or (intermethod comparison) or (placebo) or (compar*) or ((singl* or doubl* or trebl* or tripl*) "near" (blind* or mask*)) or (cross over)) or TS=(trial)
	5	#1 AND #2 AND #3 AND #4
EBSCO	1	(SU (acupuncture* OR “acupuncture therapy” OR acupoint* OR electroacupuncture* OR “electric acupuncture*” OR auriculotherapy OR “auricular acupuncture*” OR “elongated needl*” OR “scalp acupuncture*” OR “hand acupuncture*” OR “manual acupuncture*” OR “body acupuncture*” OR “warm* acupuncture*” OR “warm* needl*” OR “fire needl*” OR “trigger point*” OR meridian* OR needl* OR “abdominal acupuncture*” OR “float* needl*” OR “embedded acupuncture*” OR “intradermal acupuncture*” OR electro-acupuncture* OR micro-puncture* OR “needl* embedding” OR “dry needl*”)) OR (TI (acupuncture* OR “acupuncture therapy” OR acupoint* OR electroacupuncture* OR “electric acupuncture*” OR auriculotherapy OR “auricular acupuncture*” OR “elongated needl*” OR “scalp acupuncture*” OR “hand acupuncture*” OR “manual acupuncture*” OR “body acupuncture*” OR “warm* acupuncture*” OR “warm* needl*” OR “fire needl*” OR “trigger point*” OR meridian* OR needl* OR “abdominal acupuncture*” OR “float* needl*” OR “embedded acupuncture*” OR “intradermal acupuncture*” OR electro-acupuncture* OR micro-puncture* OR “needl* embedding” OR “dry needl*”)) OR (AB (acupuncture* OR “acupuncture therapy” OR acupoint* OR electroacupuncture* OR “electric acupuncture*” OR auriculotherapy OR “auricular acupuncture*” OR “elongated needl*” OR “scalp acupuncture*” OR “hand acupuncture*” OR “manual acupuncture*” OR “body acupuncture*” OR “warm* acupuncture*” OR “warm* needl*” OR “fire needl*” OR “trigger point*” OR meridian* OR needl* OR “abdominal acupuncture*” OR “float* needl*” OR “embedded acupuncture*” OR “intradermal acupuncture*” OR electro-acupuncture* OR micro-puncture* OR “needl* embedding” OR “dry needl*”))

	2	(SU (surger* OR operation* OR postoperati* OR postsurgical OR excision* OR resecti*) OR (TI (surger* OR operation* OR postoperati* OR postsurgical OR excision* OR resecti*)) OR (AB (surger* OR operation* OR postoperati* OR postsurgical OR excision* OR resecti*))
	3	(SU (gastroparesis OR gastropareses OR gastric stasis OR gastric stases OR “gastr* paralysis” OR “delay* gastric emptying” OR “disturbance of gastric emptying” OR “weakness of gastric emptying” OR “weakness of stomach” OR “stomach palsy” OR “gastr* motility disorder” OR “gastroparesis syndrome” OR “impaired gastric accommodation” OR “stomach paresis”)) OR (TI (gastroparesis OR gastropareses OR gastric stasis OR gastric stases OR “gastr* paralysis” OR “delay* gastric emptying” OR “disturbance of gastric emptying” OR “weakness of gastric emptying” OR “weakness of stomach” OR “stomach palsy” OR “gastr* motility disorder” OR “gastroparesis syndrome” OR “impaired gastric accommodation” OR “stomach paresis”)) OR (AB (gastroparesis OR gastropareses OR gastric stasis OR gastric stases OR “gastr* paralysis” OR “delay* gastric emptying” OR “disturbance of gastric emptying” OR “weakness of gastric emptying” OR “weakness of stomach” OR “stomach palsy” OR “gastr* motility disorder” OR “gastroparesis syndrome” OR “impaired gastric accommodation” OR “stomach paresis”))
	4	(TX “Randomized controlled trial” OR “controlled N7 “study OR design or trial”” OR “random*” OR “intermethod comparison” OR “placebo” OR “compar*” OR ““singl* OR doubl* OR trebl* OR tripl*” N “blind* OR mask*”” OR “cross over”) OR TI trial OR AB trial
	5	#1 AND #2 AND #3 AND #4
Scopus	1	TITLE-ABS-KEY (acupuncture* OR "acupuncture therapy" OR acupoint* OR electroacupuncture* OR "electric acupuncture*" OR musicotherapy OR "auricular acupuncture*" OR "elongated needl*" OR "scalp acupuncture*" OR "hand acupuncture*" OR "manual acupuncture*" OR "body acupuncture*" OR "warm* acupuncture*" OR "warm* needl*" OR "fire needl*" OR "trigger point*" OR meridian* OR needl* OR "abdominal acupuncture*" OR "float* needl*" OR "embedded acupuncture*" OR "intradermal acupuncture*" OR electro-acupuncture* OR micro-puncture* OR "needl* embedding" OR "dry needl*")
	2	TITLE-ABS-KEY (surger* OR operation* OR postoperati* OR postsurgical OR excision* OR resecti*)
	3	TITLE-ABS-KEY (gastroparesis OR gastroparesis OR gastric AND stasis OR gastric AND stases OR "gastr* paralysis" OR "delay* gastric emptying" OR "disturbance off gastric emptying" OR "weakness off gastric emptying" OR "weakness off stomach" OR "stomach palsy" OR "gastr* motility disorder" OR "gastroparesis syndrome" OR "impaired gastric accommodation" OR "stomach paresis")
	4	ALL ("Randomized controlled trial" OR "controlled W/7 " study OR design OR trial "" OR "random*" OR "intermeshed comparison" OR "placebo" OR "compar*" OR "" singl* OR doubl* OR trebl* OR tripl* " W " blind* OR mask* "" OR "cross over") OR TITLE-ABS-KEY (trial)
	5	#1 AND #2 AND #3 AND #4
OpenGrey	1	acupuncture* OR “acupuncture therapy” OR acupoint* OR electroacupuncture* OR “electric acupuncture*” OR auriculotherapy OR “auricular acupuncture*” OR “elongated needl*” OR “scalp acupuncture*” OR “hand acupuncture*” OR “manual acupuncture*” OR “body acupuncture*” OR “warm* acupuncture*” OR “warm* needl*” OR “fire needl*” OR “trigger point*” OR meridian* OR needl* OR “abdominal acupuncture*” OR “float* needl*” OR “embedded acupuncture*” OR “intradermal acupuncture*” OR electro-acupuncture* OR micro-puncture* OR “needl* embedding” OR “dry needl*”
	2	gastroparesis OR gastropareses OR gastric stasis OR gastric stases OR “gastr* paralysis” OR “delay* gastric emptying” OR “disturbance of gastric emptying” OR “weakness of gastric

		emptying” OR “weakness of stomach” OR “stomach palsy” OR”gastr* motility disorder” OR “gastroparesis syndrome” OR “impaired gastric accommodation” OR “stomach paresis”
	3	#1 AND #2
CNKI	1	术后 + 手术 + 切除
	2	胃瘫综合征 + 胃瘫 + 胃轻瘫 + 胃排空延迟 + 胃排空障碍 + 胃排空无力 + 胃无力 + 胃麻痹 + 胃动力障碍
	3	针灸 + 针刺 + 针法 + 刺法 + 穴位 + 温针灸 + 温针 + 电针 + 腹针 + 浮针 + 揿针 + 皮内针 + 埋针 + 火针 + 毫针 + 热针 + 耳针 + 头皮针 + 头针
	4	#1 AND #2 AND #3
Wanfang Database	1	术后 or 手术 or 切除
	2	胃瘫综合征 or 胃瘫 or 胃轻瘫 or 胃排空延迟 or 胃排空障碍 or 胃排空无力 or 胃无力 or 胃麻痹 or 胃动力障碍
	3	针灸 or 针刺 or 针法 or 刺法 or 穴位 or 温针灸 or 温针 or 电针 or 腹针 or 浮针 or 揿针 or 皮内针 or 埋针 or 火针 or 毫针 or 热针 or 耳针 or 头皮针 or 头针
	4	#1 AND #2 AND #3
VIP	1	术后 or 手术 or 切除
	2	胃瘫综合征 or 胃瘫 or 胃轻瘫 or 胃排空延迟 or 胃排空障碍 or 胃排空无力 or 胃无力 or 胃麻痹 or 胃动力障碍
	3	针灸 or 针刺 or 针法 or 刺法 or 穴位 or 温针灸 or 温针 or 电针 or 腹针 or 浮针 or 揿针 or 皮内针 or 埋针 or 火针 or 毫针 or 热针 or 耳针 or 头皮针 or 头针
	4	#1 AND #2 AND #3
CBM	1	术后 or 手术 or 切除
	2	胃瘫综合征 or 胃瘫 or 胃轻瘫 or 胃排空延迟 or 胃排空障碍 or 胃排空无力 or 胃无力 or 胃麻痹 or 胃动力障碍
	3	主题词："针刺 + 针刺疗法"[不加权:扩展] + 自由词 19 个
	4	#1 AND #2 AND #3
ICTRP	1	acupuncture* OR (acupuncture therapy) OR acupoint* OR electroacupuncture* OR (electric acupuncture*) OR auriculotherapy OR (auricular acupuncture*) OR (elongated needl*) OR (scalp acupuncture*) OR (hand acupuncture*) OR (manual acupuncture*) OR (body acupuncture*) OR (warm* acupuncture*) OR (warm* needl*) OR (fire needl*) OR (trigger point*) OR meridian* OR needl* OR (abdominal acupuncture*) OR (float* needl*) OR (embedded acupuncture*) OR (intradermal acupuncture*) OR electro-acupuncture* OR micro-puncture* OR (needl* embedding) OR (dry needl*)
	2	gastroparesis OR gastropareses OR gastric stasis OR gastric stases OR (gastr* paralysis) OR (delay* gastric emptying) OR (disturbance of gastric emptying) OR (weakness of gastric emptying) OR (weakness of stomach) OR (stomach palsy) OR (gastr* motility disorder) OR (gastroparesis syndrome) OR (impaired gastric accommodation) OR (stomach paresis)
	3	#1 AND #2
ClinicalTrials.gov	1	acupuncture* OR “acupuncture therapy” OR acupoint* OR electroacupuncture* OR “electric acupuncture*” OR auriculotherapy OR “auricular acupuncture*” OR “elongated needl*” OR “scalp acupuncture*” OR “hand acupuncture*” OR “manual acupuncture*”
	2	gastroparesis OR gastropareses OR gastric stasis OR gastric stases OR “gastr* paralysis” OR “delay* gastric emptying” OR “disturbance of gastric emptying” OR “weakness of gastric emptying” OR “weakness of stomach” OR “stomach palsy”
	3	#1 AND #2
ChiCTR	1	胃瘫综合征 or 胃瘫 or 胃轻瘫 or 胃排空延迟 or 胃排空障碍 or 胃排空无力 or 胃无力 or 胃麻痹 or 胃动力障碍
