# Supplementary material for: Invasive acupuncture for gastroparesis after thoracic or abdominal surgery: a systematic review and meta-analysis
Source: BMJ Open. 2023 Jun 26;13(6):e068559. doi: 10.1136/bmjopen-2022-068559 (PMC10410841; doi:10.1136/bmjopen-2022-068559)
Supplement: Supplementary data [file bmjopen-2022-068559supp004.pdf]

Supplemental Table 3 Basic characteristics of the included studies

Study ID	Study period	Age (years)	Sample size (M/F)	Surgical site (Ab/Th)	Intervention			Outcomes	Multicenter trial	Funding
					Control (mg)	Experiment	Course (days)			
Chen 2013	2009-2011	E:48-76<br>C:45-78	E:14/6<br>C:12/8	E:20/0<br>C:20/0	Mosa (15)	C; FN 30min qd	14	1)9)	No	Provincial
Chen 2016	2010-2015	E:60<br>C:58	E:15/10<br>C:14/11	E:22/3<br>C:22/3	Prokinetic	C; FN, EA, WNM 30min bid	14	1)2)3)4)9)	No	NA
Dong 2017	2010-2017	E:59.12<br>C:60.03	E:13/18<br>C:15/16	E:31/0<br>C:31/0	Meto (20)	C; FN, EA, WNM 30min bid	N/A	1)2)3)4)6)7)8)11)12)13)	No	Provincial
Huang 2020	2018-2019	E:56<br>C:56	E:36<br>C:35	E:36/0<br>C:35/0	Meto (20)	C; FN 1min bid	7	1)3)4)6)	No	NA
Li 2005	N/A	E:54<br>C:50	E:37<br>C:30	E:37/0<br>C:30/0	Cisa (30)	C; FN, EA 20min qd	7	5)	No	NA

Lin 2017	2016	E:60.50<br>C:61.00	E:27<br>C:29	E:30/0<br>C:28/2	Meto (10)	C; FN 30min qd	Until cured	3)5)6)9)	No	NA
Liu 2017	2008-2012	E:37.2<br>C:38.1	E:15/11<br>C:13/7	E:26/0<br>C:20/0	Cisa (30), EM (500)	C; FN 25min qd	20	1)2)	No	NA
Ma 2014	2007-2012	35-80	E:29<br>C:29	E:29/0<br>C:29/0	Meto (20), Mosa (30-60), EM, Seda	C; FN 30min qd	N/A	7)	No	NA
Meng 2012	2006-2011	E:56<br>C:55	E:14/10<br>C:8/4	E:24/0<br>C:12/0	Meto, Cisa, EM	C; FN, EA<br>20min qd	14	1)2)5)	Yes	NA
Sun 2010	2007-	E:61.3<br>C:59.9	E:11/7<br>C:12/6	E:0/18<br>C:0/18	Mosa (15)	C; FN 20min qd	N/A	1)	No	NA
Wang 2013	2010-2012	44.37	E:11<br>C:11	E:11/0<br>C:11/0	Prokinetic	C; FN, EA, WNM 30min qd	21	1)9)	No	NA

Yang 2010	2007-2009	E:60.66<br>C:63.7	E:11/8<br>C:11/7	E:19/0<br>C:18/0	Meto (40)	C; FN, EA<br>20min qd-bid	28	1)2)7)	No	NA
Zhang 2021	2019-2020	E:46.78<br>C:46.25	E:0/33<br>C:0/32	E:34/0<br>C:34/0	Mosa (15)	C; FN qd	7	1)2)3)4)6)7)8)	No	Provincial
Zhang 2022	2018-2020	58.58	E:14/12<br>C:13/9	E:26/0<br>C:22/0	Mosa (15), Dom (30)	C; FN 30min qd	14	1)2)5)6)9)	No	NA
Zhao 2021	2017-2019	E:62.6<br>C:62.5	E:15/18<br>C:17/15	E:28/5<br>C:25/7	Cisa (30)	C; FN, WNM 30min qd	14	1)2)5)6)10)	No	Provincial

E, experiment group; C, control group; M, male; F, female; Ab, abdominal surgery; Th, thoracic surgery; Cisa: Cisapride; EM, Erythromycin; Meto, Metoclopramide; Mosa, Mosapride; Seda, Sedative; Dom, Domperidone; FN, filiform needle; EA, electro-acupuncture; WNM, warming needle moxibustion; 1), total effective rate; 2), recovery rate; 3), motilin; 4), gastrin; 5), gastric juice volume; 6), days of gastrointestinal motility recovery; 7), days of gastrointestinal decompression; 8), days of resume diet for the first time; 9), clinical symptom score; 10), adverse events; 11), albumin; 12), hemoglobin; 13), transferrin.

Supplemental Table 4 Evidence quality of each study (GRADE)

Outcome	Quality assessment							Relative effect (95% CI)	Absolute effect	Quality
	No. of studies	Design	Risk of bias	Inconsistency	Indirectness	Imprecision	Other considerations			
Total effective rate	12	randomised trials	serious	no serious inconsistency	no serious indirectness	no serious imprecision	none	RR 1.23 (1.14 to 1.33)	-	⊕⊕⊕O MODERATE
Recovery rate	8	randomised trials	serious	no serious inconsistency	no serious indirectness	no serious imprecision	none	RR 1.55 (1.29 to 1.86)	-	⊕⊕⊕O MODERATE
Motilin	5	randomised trials	serious	serious	no serious indirectness	no serious imprecision	none	-	MD 68.15 higher (42.89 to 93.40 higher)	⊕⊕OO LOW
Gastrin	4	randomised trials	serious	no serious inconsistency	no serious indirectness	no serious imprecision	none	-	MD 40.28 higher (30.86 to 49.70 higher)	⊕⊕⊕O MODERATE
Gastric juice volume	5	randomised trials	very serious	no serious inconsistency	no serious indirectness	no serious imprecision	none	-	MD 202.95 lower (216.96 to 170.2 lower)	⊕⊕OO LOW
Days to gastrointestinal motility recovery	6	randomised trials	very serious	serious	no serious indirectness	no serious indirectness	none	-	MD 4.87 lower (7 to 1.94 lower)	⊕OOO VERY LOW

Days to gastrointestinal decompression	4	randomised trials	serious	serious	no serious indirectness	no serious indirectness	none	-	MD 3.47 lower (5.74 to 1.21 lower)	⊕⊕OO LOW
Days to resumption of diet for the first time	2	randomised trials	no serious indirectness	serious	no serious indirectness	serious	none	-	MD 2.68 lower (4.23 to 1.12 lower)	⊕⊕OO LOW
Clinical symptom score	3	randomised trials	very serious	serious	no serious indirectness	serious	none	-	MD 1.31 lower (3.24 lower to 0.61 higher)	⊕OOO VERY LOW

Supplemental Table 5 Sensitivity analysis

	Total effective rate	Recovery rate	Motilin	Gastrin	Gastric juice volume	Days to gastrointestinal motility recovery	Days to gastrointestinal decompression	Days to resumption of diet for the first time	Clinical symptom score
Random	1.18 (95% CI 1.09, 1.27)	1.46 (95% CI 1.22, 1.74)	68.15 (95% CI 42.89, 93.40)	40.28 (95% CI 30.86, 49.70)	-193.58 (95% CI -216.96, -170.20)	-4.87 (95% CI -7.80, -1.94)	-3.47 (95% CI -5.74, -1.21)	-2.68 (95% CI -4.23, -1.12)	-1.31 (95% CI -3.24, 0.61)
Fixed	1.23 (95% CI 1.14, 1.33)	1.55 (95% CI 1.29, 1.86)	60.38 (95% CI 51.05, 69.71)	38.16 (95% CI 32.59, 43.73)	-193.58 (95% CI -216.96, -170.20)	-5.41 (95% CI -5.88, -4.94)	-2.68 (95% CI -3.13, -2.23)	-2.87 (95% CI -3.56, -2.18)	-0.85 (95% CI -0.93, -0.76)

Supplemental Table 6 Egger's test

	Std_Eff	Coef.	Std. Err.	t	P>|t|	[95% Conf. Interval]	
total effective rate	slope	0.8623299	0.978937	0.88	0.399	-1.318878	3.043537
	bias	0.6521827	0.886963	0.74	0.479	-1.324094	2.628459
