# Supplementary material for: Invasive acupuncture for gastroparesis after thoracic or abdominal surgery: a systematic review and meta-analysis
Source: BMJ Open. 2023 Jun 26;13(6):e068559. doi: 10.1136/bmjopen-2022-068559 (PMC10410841; doi:10.1136/bmjopen-2022-068559)
Supplement: Supplementary data [file bmjopen-2022-068559supp001.pdf]

Supplemental Figures

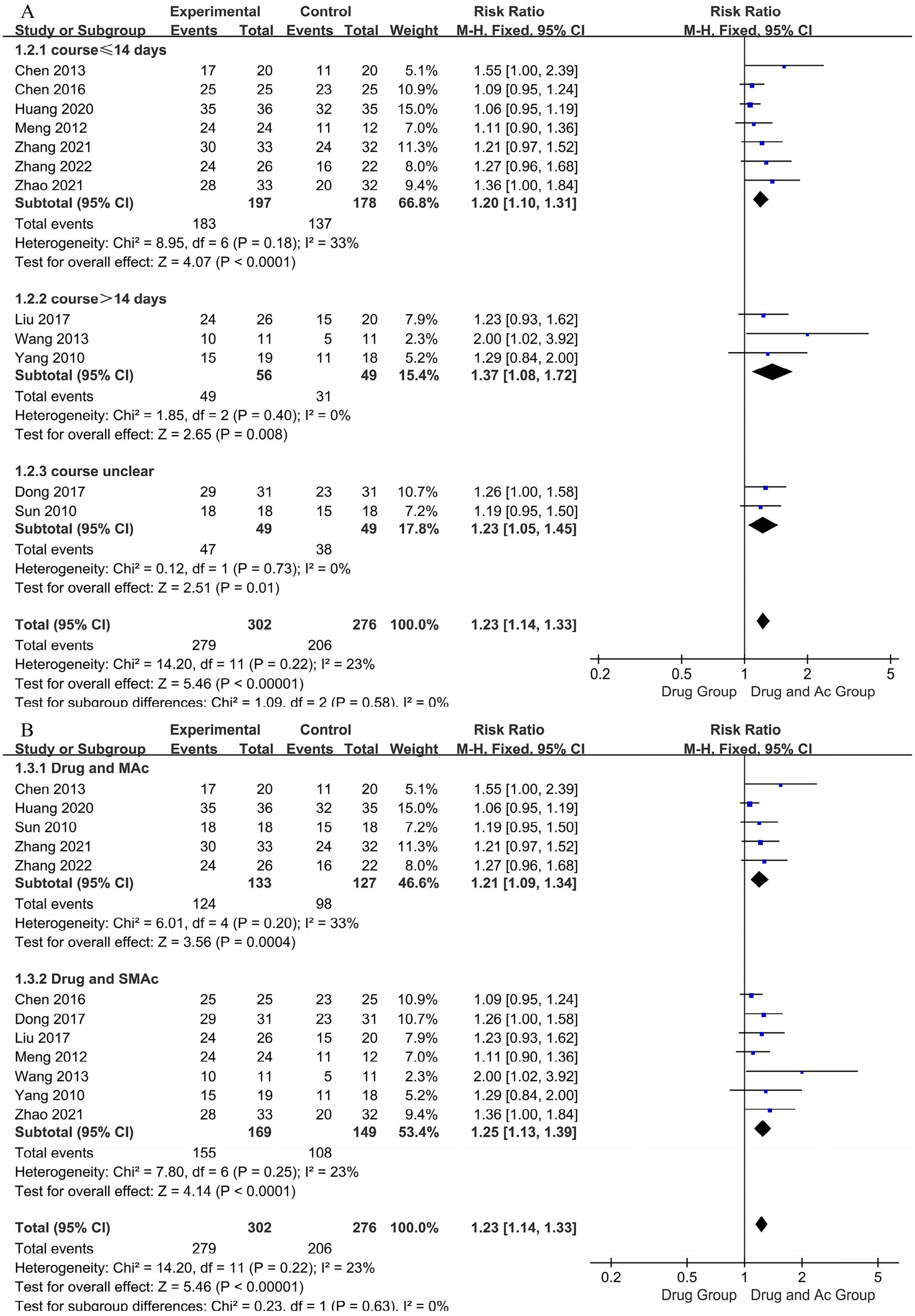

Supplemental Figure 1 Forest plots for subgroup analysis of acupuncture course(A) and acupuncture method(B) for the total effective rate.

A

Study or Subgroup	Experimental Events	Experimental Total	Control Events	Control Total	Weight	Risk Ratio M-H, Fixed, 95% CI
**8.3.1 course≤14 days**						
Chen 2016	18	25	13	25	14.9%	1.38 [0.88, 2.17]
Meng 2012	21	24	6	12	9.2%	1.75 [0.97, 3.14]
Zhang 2021	17	33	4	32	4.7%	4.12 [1.56, 10.92]
Zhang 2022	20	26	12	22	14.9%	1.41 [0.91, 2.18]
Zhao 2021	16	33	11	32	12.8%	1.41 [0.78, 2.55]
**Subtotal (95% CI)**		**141**		**123**	**56.5%**	**1.68 [1.31, 2.16]**
Total events	92		46			
Heterogeneity: Chi² = 4.96, df = 4 (P = 0.29); I² = 19%						
Test for overall effect: Z = 4.06 (P < 0.0001)						
**8.3.2 course＞14 days**						
Liu 2017	20	26	12	20	15.6%	1.28 [0.85, 1.94]
Yang 2010	15	19	11	18	13.0%	1.29 [0.84, 2.00]
**Subtotal (95% CI)**		**45**		**38**	**28.5%**	**1.29 [0.95, 1.74]**
Total events	35		23			
Heterogeneity: Chi² = 0.00, df = 1 (P = 0.98); I² = 0%						
Test for overall effect: Z = 1.64 (P = 0.10)						
**8.3.3 course unclear**						
Dong 2017	20	31	13	31	14.9%	1.54 [0.94, 2.51]
**Subtotal (95% CI)**		**31**		**31**	**14.9%**	**1.54 [0.94, 2.51]**
Total events	20		13			
Heterogeneity: Not applicable						
Test for overall effect: Z = 1.72 (P = 0.08)						
**Total (95% CI)**		**217**		**192**	**100.0%**	**1.55 [1.29, 1.86]**
Total events	147		82			
Heterogeneity: Chi² = 6.01, df = 7 (P = 0.54); I² = 0%						
Test for overall effect: Z = 4.69 (P < 0.00001)						
Test for subgroup differences: Chi² = 1.81, df = 2 (P = 0.41), I² = 0%						

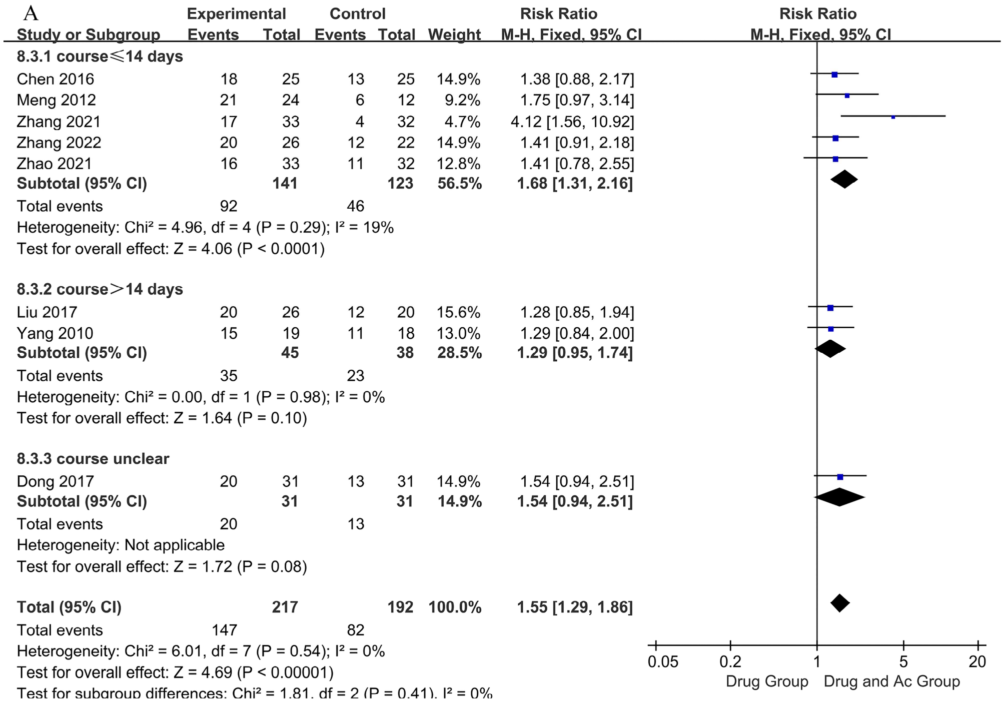

B

Study or Subgroup	Experimental Events	Experimental Total	Control Events	Control Total	Weight	Risk Ratio M-H, Random, 95% CI
**8.2.1 Drug and MAc**						
Zhang 2021	17	33	4	32	3.3%	4.12 [1.56, 10.92]
Zhang 2022	20	26	12	22	16.3%	1.41 [0.91, 2.18]
**Subtotal (95% CI)**		**59**		**54**	**19.6%**	**2.24 [0.70, 7.15]**
Total events	37		16			
Heterogeneity: Tau² = 0.57; Chi² = 4.83, df = 1 (P = 0.03); I² = 79%						
Test for overall effect: Z = 1.36 (P = 0.17)						
**8.2.2 Drug and SMAc**						
Chen 2016	18	25	13	25	15.4%	1.38 [0.88, 2.17]
Dong 2017	20	31	13	31	12.9%	1.54 [0.94, 2.51]
Liu 2017	20	26	12	20	18.0%	1.28 [0.85, 1.94]
Meng 2012	21	24	6	12	9.0%	1.75 [0.97, 3.14]
Yang 2010	15	19	11	18	16.3%	1.29 [0.84, 2.00]
Zhao 2021	16	33	11	32	8.8%	1.41 [0.78, 2.55]
**Subtotal (95% CI)**		**158**		**138**	**80.4%**	**1.40 [1.15, 1.71]**
Total events	110		66			
Heterogeneity: Tau² = 0.00; Chi² = 1.03, df = 5 (P = 0.96); I² = 0%						
Test for overall effect: Z = 3.39 (P = 0.0007)						
**Total (95% CI)**		**217**		**192**	**100.0%**	**1.46 [1.22, 1.74]**
Total events	147		82			
Heterogeneity: Tau² = 0.00; Chi² = 6.01, df = 7 (P = 0.54); I² = 0%						
Test for overall effect: Z = 4.18 (P < 0.0001)						
Test for subgroup differences: Chi² = 0.60, df = 1 (P = 0.44), I² = 0%						

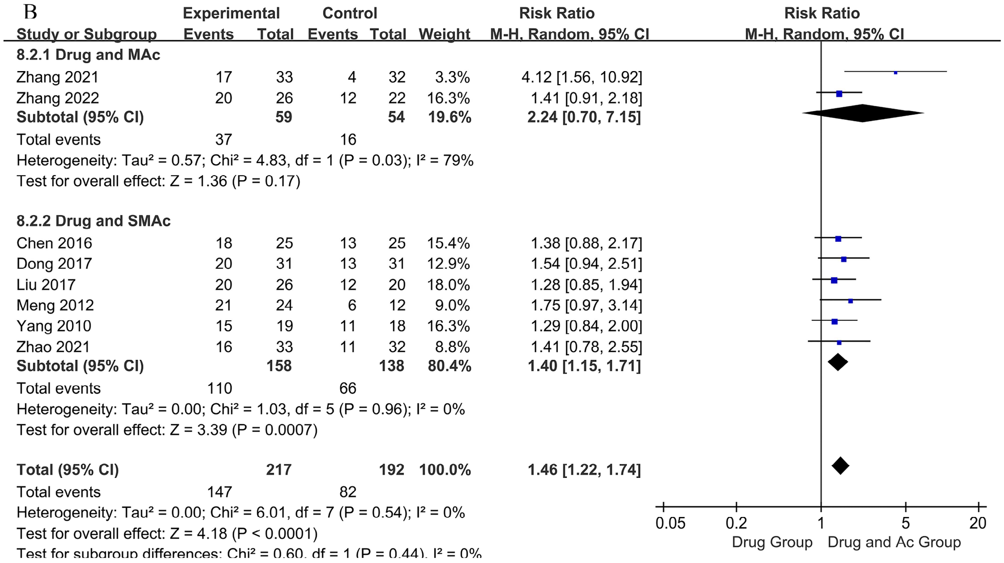

Supplemental Figure 2 Forest plots for subgroup analysis of acupuncture course(A) and acupuncture method(B) for recovery rate.

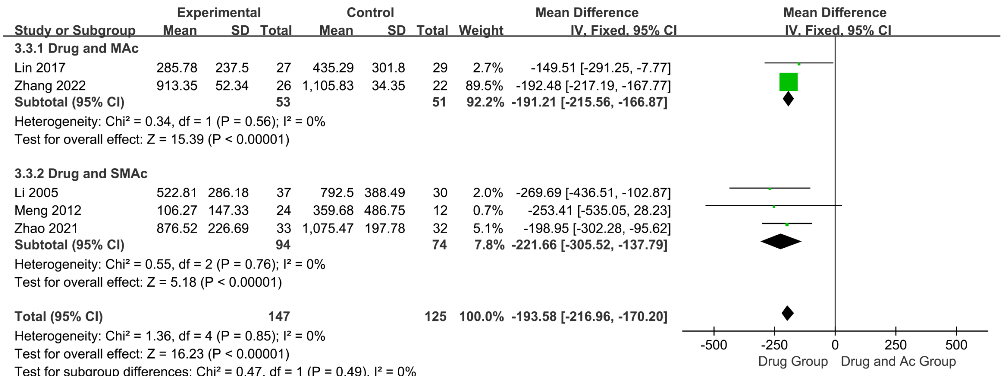

Supplemental Figure 3 Forest plot for subgroup analysis of acupuncture method for gastric juice volume.

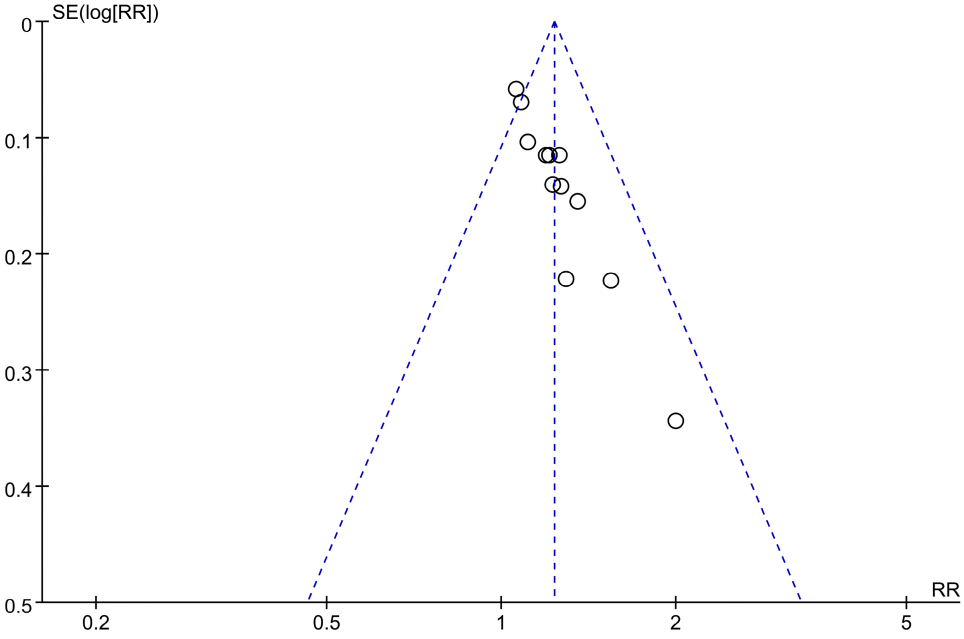

Supplemental Figure 4 Funnel plot of the total effective rate.

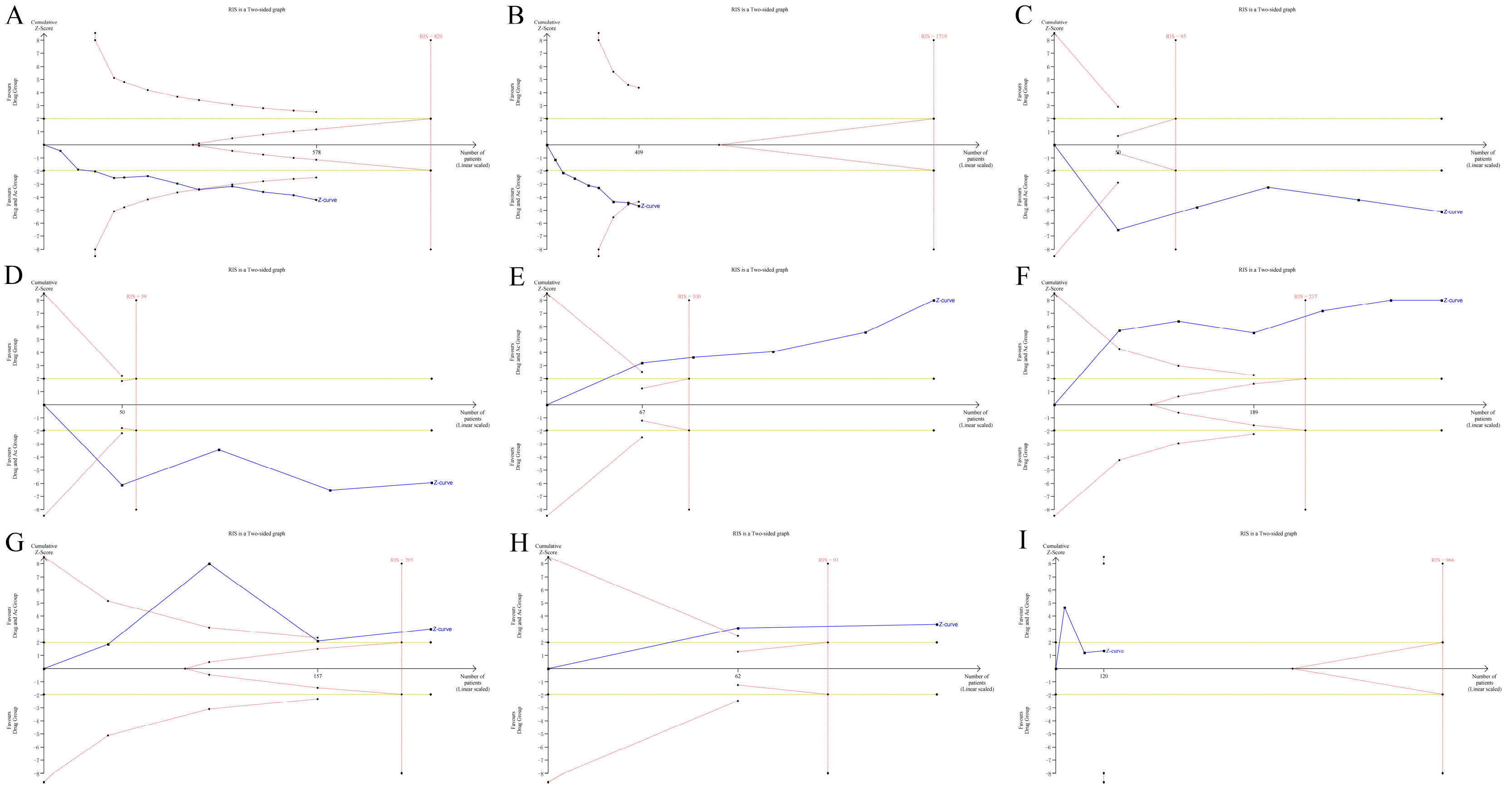

Supplemental Figure 5 TSA for (A)TER, (B)RCR, (C)MTL, (D)GSA, (E)GJV, (F)GMR, (G)GD, (H)RDT, (I)CSS. The continuous blue line represents the Z line (cumulative effect size), red dashed lines represent the trial sequential monitoring boundary and RIS, and the yellow dashed

lines represent the conventional test boundary.
