# Supplementary material for: Invasive acupuncture for gastroparesis after thoracic or abdominal surgery: a systematic review and meta-analysis
Source: BMJ Open. 2023 Jun 26;13(6):e068559. doi: 10.1136/bmjopen-2022-068559 (PMC10410841; doi:10.1136/bmjopen-2022-068559)
Supplement: Author's manuscript [file bmjopen-2022-068559.draft_revisions.pdf]

BMJ Open

BMJ Open is committed to open peer review. As part of this commitment we make the peer review history of every article we publish publicly available.

When an article is published we post the peer reviewers' comments and the authors' responses online. We also post the versions of the paper that were used during peer review. These are the versions that the peer review comments apply to.

The versions of the paper that follow are the versions that were submitted during the peer review process. They are not the versions of record or the final published versions. They should not be cited or distributed as the published version of this manuscript.

BMJ Open is an open access journal and the full, final, typeset and author-corrected version of record of the manuscript is available on our site with no access controls, subscription charges or pay-per-view fees (http://bmjopen.bmj.com).

If you have any questions on BMJ Open's open peer review process please email info.bmjopen@bmj.com

BMJ Open

Invasive acupuncture for gastroparesis after thoracic or abdominal surgery: a systematic review and meta-analysis

Journal:	*BMJ Open*
Manuscript ID	bmjopen-2022-068559
Article Type:	Original research
Date Submitted by the Author:	21-Sep-2022

Complete List of Authors:	Fan, Zhenjia; Beijing University of Chinese Medicine, Third Affiliated Hospital<br>Qiu, Yuqin; The First Affiliated Hospital of Chongqing Medical University<br>Qi, Xuewei; Beijing University of Chinese Medicine, Third Affiliated Hospital<br>Xu, Jingnan; Beijing University of Chinese Medicine, Third Affiliated Hospital<br>Wan, Yuxiang; Beijing University of Chinese Medicine, Third Affiliated Hospital<br>Hao, Yingxu; China-Japan Friendship Hospital, Oncology Department of Integrated Traditional Chinese and Western Medicine<br>Niu, Wenquan; China-Japan Friendship Hospital, Institute of Clinical Medical Sciences<br>Huang, Jinchang; Beijing University of Chinese Medicine, Third Affiliated Hospital

Keywords:	COMPLEMENTARY MEDICINE, Motility disorders < GASTROENTEROLOGY, STATISTICS & RESEARCH METHODS, SURGERY

SCHOLARONE™
Manuscripts

I, the Submitting Author has the right to grant and does grant on behalf of all authors of the Work (as defined in the below author licence), an exclusive licence and/or a non-exclusive licence for contributions from authors who are: i) UK Crown employees; ii) where BMJ has agreed a CC-BY licence shall apply, and/or iii) in accordance with the terms applicable for US Federal Government officers or employees acting as part of their official duties; on a worldwide, perpetual, irrevocable, royalty-free basis to BMJ Publishing Group Ltd ("BMJ") its licensees and where the relevant Journal is co-owned by BMJ to the co-owners of the Journal, to publish the Work in this journal and any other BMJ products and to exploit all rights, as set out in our licence.

The Submitting Author accepts and understands that any supply made under these terms is made by BMJ to the Submitting Author unless you are acting as an employee on behalf of your employer or a postgraduate student of an affiliated institution which is paying any applicable article publishing charge ("APC") for Open Access articles. Where the Submitting Author wishes to make the Work available on an Open Access basis (and intends to pay the relevant APC), the terms of reuse of such Open Access shall be governed by a Creative Commons licence – details of these licences and which Creative Commons licence will apply to this Work are set out in our licence referred to above.

Other than as permitted in any relevant BMJ Author's Self Archiving Policies, I confirm this Work has not been accepted for publication elsewhere, is not being considered for publication elsewhere and does not duplicate material already published. I confirm all authors consent to publication of this Work and authorise the granting of this licence.

For peer review only

Invasive acupuncture for gastroparesis after thoracic or abdominal surgery: a systematic review and meta-analysis

Zhenjia Fan[1], Yuqin Qiu[2], Xuewei Qi[1], Jingnan Xu[1], Yuxiang Wan[1], Yingxu Hao[3*], Wenquan Niu[4*], Jinchang Huang[1*]

*** Correspondence:**
Jinchang Huang, zryhhuang@163.com; Wenquan Niu, niuwenquan_shcn@163.com; Yingxu Hao, haoyingxu@126.com

Word count: 4633 words.

For peer review only

ABSTRACT

Objectives This meta-analysis aims to systematically evaluate the efficacy and safety of acupuncture in the treatment of postsurgical gastroparesis syndrome (PGS) after thoracic or abdominal surgery.

Design Systematic review and meta-analysis.

Data sources Twelve databases and three trial registration websites were searched since creation to September 2022, and citations to the included literature were screened.

Eligibility criteria All randomized controlled trials addressing invasive acupuncture for PGS.

Data extraction and synthesis Key information of included studies was completed by two reviewers independently. Risk ratio (RR) with 95%CI was used for categorical data, while mean difference (MD) with 95%CI for continuous data. The quality of evidence was assessed with GRADE.

Results Fifteen studies met inclusion criteria and 759 patients were included. This meta-analysis demonstrates that acupuncture had a greater positive effect on the TER (9 trials, n = 427; RR = 1.20; 95%CI 1.08 to 1.32; I^2 =28 %, P = 0.0004) and RR (6 trials, n = 294; RR = 1.61; 95%CI 1.30 to 1.98; I^2 =19 %, P < 0.0001) of PGS after abdominal surgery. Relatively, there was no significant advantage in the TER of acupuncture for thoracic surgery (1 trials, P = 0.13), thoracic/abdominal surgery related PGS (2 trials, n = 115; RR = 1.18; 95%CI 0.89 to 1.57; I^2 =67 %, P = 0.24), and similar results were obtained for the RR of thoracic/abdominal surgery related PGS (2 trials, n = 115; RR = 1.40; 95%CI 0.97 to 2.01; I^2 =0 %, P = 0.07). There was only one study reported acupuncture-related adverse event of mild local subcutaneous hemorrhage and pain that recovered spontaneously.

Conclusion Based on this meta-analysis, acupuncture has a significant advantage in the treatment of PGS associated with abdominal surgery, while it hasn't in PGS associated with thoracic surgery.

PROSPERO registration number CRD42022299189

Keywords: COMPLEMENTARY MEDICINE, Motility disorders < GASTROENTEROLOGY, STATISTICS & RESEARCH METHODS, SURGERY

STRENGTHS AND LIMITATIONS OF THIS STUDY

1. This systematic review and meta-analysis was performed according to the Preferred Reporting Items for Systematic Reviews and Meta-Analyses (PRISMA) statement to ensure reproducibility of the study.

2. A comprehensive search strategy was used to search through a large number of databases and registration websites, and gray literatures were included in the study.

3. The quality of evidence for each outcome were assessed with GRADE, and publication bias assessed with Stata 16 software.

4.Outcomes were analyzed by subgroup according to surgical site, acupuncture stimulation methods and intervention courses.

5. The included outcomes were immediate post-intervention time-point data, leading to a lack of analysis of the long-term effects of the intervention in this study.

INTRODUCTION

PGS is a gastric motility disease defined by delayed gastric emptying after eating without pyloric stenosis and other mechanical obstruction within 3 months postoperatively, with symptoms including nausea, vomiting, early satiety, postprandial fullness, and bloating[1-3]. PGS is the second most common gastroparesis after Diabetes Gastroparesis and it often occurs after abdominal surgery,[2] with incidence rate of 0.4%~5.0% in gastric surgery.[4 5] In addition, it has been reported to occur after thoracic surgery such esophageal surgery,[6 7] lung surgery[8 9] and heart transplantation,[10 11] which can negatively impact postoperative patients' quality of life.

Treatment of PGS consists of dietary modification, gastrointestinal decompression, fluid and electrolyte acid-base balance, nutritional support, psychological therapies, prokinetic and antiemetic medication, with limited benefit.[1] Additional therapeutic options such as pyloric botulinum toxin injections, gastric electrical stimulation (GES), per-oral pyloromyotomy (POP), and gastric peroral endoscopic myotomy (G-POEM) are considered for PGS when medication fails, all of which are invasive and have limited efficacy.[12 13] The gastrointestinal total symptom score (TSS) was reduced by 48% in European patients treated with GES, while it was reduced by 38% in the United States, according to the study.[14] It shows that the effect of modern medical treatment on PGS is unsatisfied. Acupuncture, or a combination of acupuncture and drugs, has been reported to have some advantages in the treatment of PGS in recent clinical trials.[15 16]

Invasive acupuncture, which involves piercing one or more needles into specific acupoints on the body surface, is a traditional Chinese medicine treatment method that is distinct from other local stimulation therapies such as transcutaneous electrical nerve stimulation.[17] Several studies have documented the mechanism of acupuncture for PGS as follows: ①Regulating the secretion of gastrointestinal hormones. Yu et al.[18] found that acupuncturing at Zhongwan (CV12) and Zusanli (ST36) can promote the levels of motilin, gastrin, ghrelin, and growth factor 1 in the serum of rats to treat gastroparesis; ②Stimulating the vagus nerve. The pathogenesis of PGS may be related to vagus nerve injury or compression and impaired gastric muscle regulation.[1] Yang et al.[19] and Eitaro[20] found that electro-acupuncturing on the ST36 on the hind limb stimulates the vagus nerve, which attenuates motility dysfunction in intestinal muscularis and modulate the gastrointestinal motility. ③Reducing damage to interstitial cells of Cajal (ICC). ICC cells are considered pacemaker cells in the

For peer review only

gastrointestinal tract, transmitting electrical signals for gastric emptying.[1] Song et al.[21] found the improvement of gastroparesis by acupuncture may be related to reducing the damage of the ICCs.

Several previous studies have observed therapeutic efficacies of acupuncture on PGS such as improving symptoms of nausea, vomiting, early satiety and bloating, reducing the time of resume diet for the first time, promoting gastric emptying, downgrading gastric juice volume, upgrading motilin and gastrin in serum.[16 22-24]

The lack of convincing evidence for the efficacy of acupuncture in the treatment of PGS due to small sample size and different trial protocols has hampered its clinical application. This study reviews RCTs of acupuncture for PGS systematically by meta-analysis to provide an evidence-based medical data for the clinical application of acupuncture.

METHODS

Study registration

The protocol for this systematic review and meta-analysis was registered in PROSPERO (CRD42022299189) and performed according to the Preferred Reporting Items for Systematic Reviews and Meta-Analyses (PRISMA) statement as shown in online supplemental table 1.[25]

Data sources and search strategy study registration

We searched, with no language restrictions, the following 12 databases: PubMed, Embase, Cochrane Library Cochrane Central Register of Controlled Trials (CENTRAL), Medline(Ovid)(from 1946), Web of Science, EBSCO, Scopus, OpenGrey, China National Knowledge Infrastructure (CNKI), Wanfang Database, Chinese Scientific Journals Database (VIP), China Biology Medicine disc (CBM), and 3 registration websites: World Health Organization International Clinical Trials Registry Platform(ICTRP), ClinicalTrials.gov, ChiCTR since creation to September 2022 for systematic reviews of RCTs. Search strategies were detailed in online supplemental table 2. We adapted search strategy appropriately for each database. In addition, we screened citations of included articles manually to avoid search omissions.

Study selection

We screened titles and abstracts of articles by Endnote X7 software, and the full-texts were screened according to the selection criteria of the study.

Inclusion criteria: ①Participants of trails diagnosed with PGS.[4] There is no standardized criteria for PGS diagnosis, this study adopted the diagnosis formulated by Professor Qin's team at Zhongshan Hospital, Fudan University, Shanghai, China as the criteria after researching the relevant documents.[1 3 26-28] ②The control group of the trial was treated with prokinetic drugs in addition to the routine treatment. ③The experiment group of the study was treated with acupuncture on the basis of the control group. ④Acupuncture is the penetration of a filiform needle invasive into the skin with electrical or moxibustion stimulation or not. ⑤The trial was an RCT, with no blind restrictions.

Exclusion criteria: ①Duplicated publications. ②The experiment group of the study used other Chinese medical treatments in addition to acupuncture, including but not limited to oral herb, non-invasive external therapies such as moxibustion without acupuncture, acupressure, acupoint

For peer review only

application and herbal enema. ③Missing or uncleared data, and inability to contact the author for detail. ④Animal or cell experiments. ⑤Reviews.

Two reviewers screened and selected articles independently. It was resolved by discussion with a third reviewer when disagreements arisen between reviewers.

Outcome assessment

The primary outcomes of the study were total effective rate and recovery rate. The secondary outcomes were serum motilin, serum gastrin, gastric juice volume, days of gastrointestinal motility recovery, days of gastrointestinal decompression, days of resume diet for the first time, and clinical symptom score.

Data extraction and management

We extracted and sorted relevant data of included studies by Microsoft Excel 2019 and displayed in a table. The data included: the first author, year of publication, trial duration, number and age of patients in each group, surgical site, treatment methods and courses of intervention and control groups, acupoint, and outcomes (dichotomous data were number of events and number of participants per group; continuous data were presented as mean, standard deviation (SD), and number of participants per group).

Data extraction and management completed by two reviewers independently and a third reviewer joined the discussion and made a decision when disagreements arisen between reviewers.

Assessment of risk of bias and quality of evidence

Two reviewers independently evaluated the risk bias of the included studies based on the Cochrane Handbook which contains seven items: 1) Random sequence generation (selection bias); 2) Allocation concealment (selection bias); 3) Blinding of participants and personnel (performance bias); 4) Blinding of outcome assessment (detection bias); 5) Incomplete outcome data (attrition bias); 6) Selective reporting (reporting bias); and 7) other biases.[29] Three levels (high risk, low risk, and unclear risk) were used during evaluating. Any disagreements during the assessment process were to be discussed and evaluated by a third senior professional. Review Manager 5.3 software was used for building the summary and graph of the risk of bias. Furthermore, this study assessed the quality of evidence for each outcome and expressed it in terms of high, medium, low or very low with GRADE pro 3.6.[30 31]

Statistical analysis

The systematic review and meta-analysis were completed with Review Manager 5.3 software. During data analysis, risk ratio (RR) with 95% confidence intervals (CIs) was used for categorical data, while mean difference (MD) with 95% confidence intervals (CIs) was used for continuous data. It was considered statistically significant when $p < 0.05$. Heterogeneity of review was assessed by the I^2 statistic. If there is significant heterogeneity among studies (I^2>50%), the random effects model will be used, otherwise the fixed effects model will be used when $I^2 \leq 50\%$. Subgroup studies were conducted according to surgical site, acupuncture stimulation methods and intervention courses for heterogeneity analysis. Sensitivity analysis was performed to determine the stability of the results by converting the fixed effect model and random effect model, and removing single study in turn to

For peer review only - http://bmjopen.bmj.com/site/about/guidelines.xhtml

observed its impact on the summary results. Publication bias assessed with Stata 16 software and the funnel diagram plotted with Review Manager 5.3 software.

Patient and public involvement statement

Patients or the public were not involved.

RESULTS

Study selection

The flowchart of article screening process in the meta-analysis is shown in figure 1, which was derived from the PRISMA statement.[25] A total of 2549 articles were searched, 2513 of which from databases and 36 from registers. 121 articles were selected for full text screening by reading the titles and abstracts. 14 articles[32-45] were finally included after 107 articles were excluded for reasons such as unclear diagnosis, inappropriate intervention method, study was not a RCT, or unclear data. Besides, we screened 262 articles cited in 14 included articles, and 1 of these met inclusion criteria.[46] As a result, a total of 15 studies were included in this meta-analysis.

Study characteristics

The basic characteristics of the included studies are presented in online supplemental table 3. The included studies were all completed in China and included 759 patients, with sample sizes ranging from 22 to 71 participants. One of the 15 studies was a multi-center trial, and the rest were single-center trials. The mean age of patients was over 50 years in 10 studies, under 50 years in 3 studies and it was not mentioned in 2 other studies. Participants of 3 studies were PGS after thoracic or abdominal surgery, 1 study was simple thoracic surgery, and the rest were simple abdominal surgery. The control group (Drug Group) was treated with prokinetic drugs in all included studies and the experimental group (Drug and Ac Group) was treated with filiform needle on the basis of the control group. The experimental group in 8 of the studies intervened with individual filiform needle named manual acupuncture (MAc) and 7 studies added electrical stimulation or moxibustion stimulation based on filiform needle which named stimulated manual acupuncture (SMAc). A number of studies intervened multiple acupoints except Huang 2020, which stimulated only a pair of acupoints. There were 11 studies have clearly established the treatment course, of which 8 studies had a treatment course of ≤ 14 days and 3 studies had a treatment course of >14 days. The frequency of acupuncturing was once daily in 11 studies, twice daily in 3, and between once and twice daily in the other 1. For primary outcomes, 12 studies reported total effective rates and 8 reported recovery rates. Four studies received financial support from provincial projects.

Risk of bias and study quality

All studies mentioned a random design, with ten (66.67%) reported random sequence generation processes clearly and only one (6.67%) described the blinding of participants. Four studies (26.67%) reported case shedding. The summary results of the risk of bias are detailed in figure 2. The result of the GRADE shows that the outcomes of total effective rate, recovery rate and gastrin are medium quality; motilin, gastric juice volume, days of gastrointestinal decompression, and days of resume diet for the first time are low quality, days of gastrointestinal motility recovery and clinical symptom score are very low quality (online supplemental table 4).

Main outcomes

Total effective rate (TER)

12 trials reported the TER of which the results were shown in figure 3A. There were 302 patients in Drug and Ac Group and 276 in Drug Group. Test for overall effect indicated that the TER of Drug and Ac Group was significantly higher than that of Drug Group (12 trials, n = 578; RR = 1.18; 95%CI 1.09 to 1.27; I^2 =23%, P < 0.0001). The sensitivity analysis showed that the study reported by Huang 2020 was the source of instability, which may be related to acupuncturing at only one pair of acupoints (online supplemental table 5).

The results of a subgroup analysis of surgical sites revealed that Drug and Ac Group has a statistically significant advantage in the TER of PGS after abdominal surgery (9 trials, n = 427; RR = 1.20; 95%CI 1.08 to 1.32; I^2 =28 %, P = 0.0004) while it has no significant advantage in the TER of PGS after thoracic surgery (1 trials, P = 0.13) and thoracic/abdominal surgery (2 trials, n = 115; RR = 1.18; 95%CI 0.89 to 1.57; I^2 =67 %, P = 0.24)(figure 3A). Furthermore, subgroup analysis revealed that overall effect of TER is not significantly related to the acupuncture course (online supplemental figure 1A) or the method of acupuncture stimulation (individual filiform needle, electrical stimulation or moxibustion stimulation) (online supplemental figure 1B).

Recovery rate (RR)

8 studies reported the RR of which the results were shown in figure 3B. There were 217 patients in Drug and Ac Group and 192 in Drug Group. Test for overall effect indicated that the RR of Drug and Ac Group was significantly higher than that of Drug Group (8 trials, n = 409; RR = 1.55; 95%CI 1.29 to 1.86; I^2 = 0%, P < 0.00001). Sensitivity analysis shows that the conclusion is stable (online supplemental table 5).

The results of a subgroup analysis of surgical sites revealed that Drug and Ac Group has a statistically significant advantage in the RR of PGS after abdominal surgery (6 trials, n = 294; RR = 1.61; 95%CI 1.30 to 1.98; I^2 =19 %, P <0.0001) while it has no significant advantage in the RR of PGS after thoracic/abdominal surgery (2 trials, n = 115; RR = 1.40; 95%CI 0.97 to 2.01; I^2 =0 %, P = 0.07) (figure 3B). The results of a subgroup analysis of acupuncture course revealed that Drug and Ac Group has a statistically significant advantage in the RR of PGS when course ≤14 days (5 trials, n = 264; RR = 1.68; 95%CI 1.31 to 2.16; I^2 =19%, P < 0.0001), but it has no significant advantage in the RR of PGS when course ＞14 days (2 trials, n = 83; RR = 1.29; 95%CI 0.95 to 1.74; I^2 =0 %, P = 0.10)(online supplemental figure 2A). The results of a subgroup analysis of the method of acupuncture stimulation revealed that Drug and Ac Group has a significant advantage in the RR of PGS when treated with SMAc (6 trials, n = 296; RR = 1.40; 95%CI 1.15 to 1.71; I^2 =0%, P = 0.0007), but it has no significant advantage when treated with MAc (2 trials, n =113; RR = 2.24; 95%CI 0.70 to 7.15; I^2 =79%, P = 0.17) (online supplemental figure 2B).

Secondary outcomes

Motilin (MTL)

5 studies reported the MTL of which the results were shown in figure 4A. There were 152 patients in Drug and Ac Group and 152 in Drug Group. Test for overall effect indicated that the MTL of Drug and Ac Group was significantly higher than that of Drug Group (5 trials, n = 304; MD = 60.37; 95%CI 37.26 to 83.47; I^2 = 89%, P < 0.00001). The sensitivity analysis showed that the study reported by Lin 2017 was the source of instability, which may be related to the high risk of bias (online supplemental table 5).

For peer review only - http://bmjopen.bmj.com/site/about/guidelines.xhtml

The results of a subgroup analysis of surgical sites revealed that Drug and Ac Group has a statistically significant advantage in the MTL of PGS after both abdominal surgery (3 trials, n = 198; MD = 44.28; 95%CI 28.84 to 59.71; I^2 = 66 %, $P < 0.00001$) and thoracic/abdominal surgery (2 trials, n = 106; MD = 89.21; 95%CI 8.21 to 170.22; I^2 = 96 %, $P = 0.03$), despite with high heterogeneity (figure 4A).

Gastrin (GAS)

4 studies reported the GAS of which the results were shown in figure 4B. There were 125 patients in Drug and Ac Group and 123 in Drug Group. Test for overall effect indicated that the GAS of Drug and Ac Group was significantly higher than that of Drug Group (4 trials, n = 248; MD = 47.90; 95%CI 32.15 to 63.64; I^2 = 76 %, $P < 0.00001$). Sensitivity analysis shows that the conclusion is stable despite with high heterogeneity (online supplemental table 5). However, the cause of heterogeneity has not been found, and higher quality trials may be needed for further study.

The results of a subgroup analysis of surgical sites revealed that Drug and Ac Group has a statistically significant advantage in the GAS of PGS after both abdominal surgery (3 trials, n = 198; MD = 40.97; 95%CI 29.07 to 52.88; I^2 = 56 %, $P < 0.00001$) and thoracic/abdominal surgery (1 trials, $P < 0.00001$) (figure 4B).

Gastric juice volume (GJV)

5 studies reported the GJV of which the results were shown in figure 4C. There were 147 patients in Drug and Ac Group and 125 in Drug Group. Test for overall effect indicated that the GJV of Drug and Ac Group was significantly lower than that of Drug Group (5 trials, n = 272; MD = -193.58; 95%CI -216.96 to -170.20; I^2 = 0 %, $P < 0.00001$). Sensitivity analysis shows that the conclusion is stable (online supplemental table 5).

The results of the subgroup analysis revealed that the advantage of Drug and Ac Group in regulating GJV was not affected by the surgical site (figure 4C) or the acupuncture method (online supplemental figure 3A).

Days of gastrointestinal motility recovery (GMR)

6 studies reported the days of GMR of which the results were shown in figure 5A. There were 186 patients in Drug and Ac Group and 181 in Drug Group. Test for overall effect indicated that the days of GMR of Drug and Ac Group was significantly fewer than that of Drug Group (6 trials, n = 367; MD = -4.87; 95%CI -7.80 to -1.94; I^2 = 97 %, $P = 0.001$). Sensitivity analysis shows that the conclusion is stable despite with high heterogeneity (online supplemental table 5).

The results of the subgroup analysis revealed that the advantage of Drug and Ac Group in regulating GMR was not affected by the surgical site (figure 5A).

Days of gastrointestinal decompression (GD)

4 studies reported the days of GD of which the results were shown in figure 5B. There were 112 patients in Drug and Ac Group and 110 in Drug Group. Test for overall effect indicated that the days of GD of Drug and Ac Group was significantly fewer than that of Drug Group (4 trials, n = 222; MD = -3.47; 95%CI -5.74 to -1.21; I^2 = 94 %, $P = 0.003$). The sensitivity analysis showed that the study reported by Ma 2014 was the source of instability (online supplemental table 5), which we are

For peer review only - http://bmjopen.bmj.com/site/about/guidelines.xhtml

unsure whether this is related to the treatment of sedatives in some of the participants in this trial, as sedatives can affect vagal activity.[47]

Days of resume diet for the first time (RDT)

2 studies reported the days of RDT of which the results were shown in figure 5C. There were 64 patients in Drug and Ac Group and 63 in Drug Group. Test for overall effect indicated that the days of RDT of Drug and Ac Group was significantly fewer than that of Drug Group (2 trials, n = 127; MD = -2.68; 95%CI -4.23 to -1.12; I^2 = 78 %, P = 0.0007). Sensitivity analysis shows that the conclusion is stable despite with high heterogeneity (online supplemental table 5). The reliability of this conclusion is low due to the lack of data.

Clinical symptom score (CSS)

5 studies reported the CSS of which the results were shown in figure 5D. There were 109 patients in Drug and Ac Group and 107 in Drug Group. Test for overall effect indicated that the CSS of Drug and Ac Group was lower than that of Drug Group but the difference was not statistically significant (5 trials, n = 216; MD = -1.07; 95%CI -2.24 to 0.11; I^2 =100%, P = 0.08).

The results of a subgroup analysis of surgical site revealed that Drug and Ac Group has no statistically significant advantage in the CSS of PGS after either abdominal surgery (3 trials, n = 110; MD = -0.18; 95%CI -0.43 to 0.06; I^2 = 72 %, P = 0.14) or thoracic/abdominal surgery (2 trials, n = 106; MD = -1.97; 95%CI -5.16 to 1.23; I^2 = 100 %, P = 0.23) (figure 5D). In a subgroup analysis based on the acupuncture frequency revealed that Drug and Ac Group had a statistically significant advantage in the CSS of PGS both for once-daily (4 trials, n = 166; MD = -0.24; 95%CI -0.40 to -0.07; I^2 = 73 %, P = 0.004) and twice-daily acupuncturing (1 trials, P< 0.00001) (online supplemental figure 3B).

Safety analysis

There were 2 studies described the safety of the trials. Dong 2017 assessed nutritional indicators such as albumin, hemoglobin and transferrin before and after the intervention, and the results showed that all three indicators were significantly higher in the experiment and control groups at the end of the intervention compared to the pre-intervention period, and the experiment group was significantly higher than the control group, with statistically significant differences (P<0.05). Zhao 2021 recorded adverse events in the study, with subcutaneous hemorrhage and pain associated with acupuncture in the experiment group, all of which were mild and recovered on their own without special treatment. There were no acupuncture-related serious adverse events in experimental group and control group were reported.

Publication bias

The funnel plot of the TER was symmetric (online supplemental figure 4) and Egger's test showed p = 0.399 ＞ 0.05 (online supplemental table 6), indicating that there was low risk of publication bias in the included studies.

Frequency analysis of acupoints

For peer review only - http://bmjopen.bmj.com/site/about/guidelines.xhtml

The 14 studies involved 26 acupoints with a frequency of 88. The acupoints that were applied ≥3 times are listed in table 1 according to the application frequency from high to low, with the top three are ST36, CV12 and PC6.

Table 1 Summary of frequency of applicated acupoints (Top 9)

No.	Acupoint	Alphabetic Code	Frequency
1	Zusanli	ST36	15
2	Zhongwan	CV12	12
3	Neiguan	PC6	11
4	Shangjuxu	ST37	8
5	Tianshu	ST25	6
6	Sanyinjiao	SP6	6
7	Gongsun	SP4	5
8	Qihai	CV6	4
9	Yinlingquan	SP9	3

DISCUSSION

This study aims to evaluate the efficacy and safety of acupuncture in the treatment of PGS. 15 studies containing 759 patients were included after systematic searching and screening. Meta-analysis suggests that, overall, the treatment of acupuncture combined with prokinetic drugs had a more positive efficacy on the TER, RR, MTL, GAS, GJV, days of GMR, days of GD and days of RDT in PGS compared to the treatment of prokinetic drugs alone, and although there was a trend towards a positive efficacy of acupuncture combined with drugs on CSS, the difference was not statistically significant. The results of a subgroup analysis of the main outcomes suggests that the advantage of the TER of acupuncture combined with prokinetic drugs for PGS did not appear to be influenced by the course of the acupuncture, or the method of acupuncture stimulation, however, the RR of acupuncture for PGS showed a statistically significant advantage only for short course. It is noteworthy that acupuncture combined with prokinetic drugs has no obvious advantage in the TER and RR of PGS after thoracic surgery and thoracic/abdominal surgery. Relatively, acupuncture combined with prokinetic drugs can significantly improve the TER and RR of PGS after abdominal surgery. In addition, the therapeutic advantage of secondary outcomes in acupuncture combined with prokinetic drugs group was not affected by acupuncture course, methods, frequency or surgical site, although there was heterogeneity. In terms of safety analysis, only one study reported mild adverse

For peer review only - http://bmjopen.bmj.com/site/about/guidelines.xhtml

events associated with acupuncture and one other reported the results of albumin, hemoglobin, transferrin in serum.

At present, prokinetic drugs are the most commonly used in PGS medication. Metoclopramide, a D2 receptor antagonist, with some 5-HT4 receptor agonists at the same time. As the only drug approved by FDA for gastroparesis， it can effectively alleviate PGS nausea, vomiting and other symptoms of digestive system.[48] 5-HT4 agonists such as cisapride are frequently and early adopted in PGS and mosapride has also been used in gastroparesis.[49-52] Prucalopride is a recently discovered effective drug that accelerates gastric emptying without cardiac side effects and has been initially reported to relieve symptoms in patients with idiopathic gastroparesis or diabetic gastroparesis,[53] but high-quality studies of PGS are lacking. Macrolide antibiotics such as erythromycin are agonists of gastrin receptors, which induce gastrin secretion in the proximal part of the digestive tract and accelerate gastric emptying. However, it faces problems such as drug resistance rapidly and its clinical benefit is controversial.[54,55]

The results of the analysis showed that acupuncture combined with prokinetic drugs for the PGS was able to significantly improve the efficacy of the treatment and digestive symptoms of patients. Meanwhile, acupuncture is less traumatic and no serious adverse events have been reported compared to endoscopic and surgical treatments such as pyloric botulinum toxin injections, GES, POP and G-POEM.[56]

Among the 15 studies included in this meta-analysis, the ST36 on the ST meridian, the CV12 on the CV meridian and the PC6 on the PC meridian are the most frequently adopted, and all three acupoints play an important role in regulating stomach function. It has been reported that acupuncturing at the ST36 can contract muscles,[57] regulate gastric rhythm and improve gastric emptying delay by stimulating the parasympathetic nerves to treat gastroparesis.[58] Further study indicated that electro-acupuncturing at ST36 can modulate gastric motility and improve gastrointestinal symptoms through M2/3 and β1/2 receptor-mediated vagal and sympathetic reflex.[59] Electro-acupuncturing at CV12 can regulate gastric emptying and demonstrate beneficial effects on nausea and vomiting which may be mediated through inhibition of 5-HT secretion in the duodenum and activity of the nucleus of the solitary tract.[60,61] Stimulating at PC6 has an effect on gastrointestinal symptoms and prevents postoperative nausea and vomiting.[62-64] Electro-acupuncturing at PC6 reduces the inhibition of efferent vagus nerve motor fibers by inhibiting GABA transmission to DMV, thus promoting efferent vagus nerve activity and increasing gastric motility.[65] In clinical, ST36, CV12 and PC6 are applied frequently in combination to improve digestive symptoms, and have been proved to be effective.[66]

In general, the meta-analysis of previous studies of acupuncture for PGS found that acupuncture combined with prokinetic drugs did improve the TER and RR of PGS. However, more evidences needed on the relationship between surgical site and the TER, RR, and the relationship between the RR and the acupuncture course, the method of acupuncture stimulation. In addition, acupuncture can up-regulate the MTL and GAS in serum, decrease GJV, accelerate GMR, improve CSS, shorten days of GD and RDT as soon as possible. It shows that acupuncture seems to be safety through the analysis, although more trails needed to verify it.

However, this meta-analysis has some limitations. Firstly, as mentioned, 759 participants were included in this study, which is a small sample analysis and exists risk of bias. Secondly, all included studies were completed in China and there may be racial and geographical variability in the meta-analysis. Thirdly, the types, doses and methods of prokinetic drugs used in the studies were

For peer review only - http://bmjopen.bmj.com/site/about/guidelines.xhtml

inconsistent. Fourthly, some of the outcome evaluation indicators are not uniform, such as the CSS, although it took nausea, vomiting, early satiety, abdominal distension and other PGS symptoms as the main evaluation indicators. Fifthly, the grades of PGS were not mentioned in included studies, which may be a key factor to influence the therapeutic effect of acupuncture on PGS. Sixthly, the thoracic surgeries involved in this meta-analysis were all esophagus cancer surgeries, which led to limited data, and perhaps further in-depth studies could demonstrate the efficacy of acupuncture for PGS in thoracic surgery reliably. Finally, only two included studies reported safety data and more trails demanded.

CONCLUSIONS

This meta-analysis demonstrated that compared with the use of prokinetic drugs alone, acupuncture with prokinetic drugs for PGS associated with abdominal surgery is more advantageous. It's worthy for clinical promoting because of the improvement in efficacy and clinical symptoms without serious adverse events. However, the efficacy of acupuncture for PGS associated with thoracic surgery is unclear. Due to the limitations of the included studies, high quality trials with rigorous design and large sample sizes are needed to further confirm the efficacy and safety of acupuncture for PGS.

Author affiliations

[1]Third Affiliated Hospital, Beijing University of Chinese Medicine, Beijing, China

[2]First Affiliated Hospital, Chongqing Medical University, Chongqing, China

[3]Oncology Department of Integrated Traditional Chinese and Western Medicine, China-Japan Friendship Hospital, Beijing, China

[4]Institute of Clinical Medical Sciences, China-Japan Friendship Hospital, Beijing, China

Contributors Huang J proposed the original concept and Fan Z designed the study. Qiu Y and Qi X screened the abstract and full text. Qi X and Xu J extracted the data. Wan Y and Xu J evaluated the risks of bias. Fan Z and Qiu Y carried out the outcomes and wrote the manuscript. Hao Y revised the manuscript and arbitrated disagreements. Niu W guided the meta-analysis.

Funding This work was supported by Key Research Project of Beijing University of Chinese Medicine (Grant No. 2020-JYB-ZDGG-143-1), National Natural Science Foundation of China (Grant No. 82074545), Hospital-level Cultivation Project of the Third Affiliated Hospital of Beijing University of Chinese Medicine (Grant No. BZYSY-2022-PYQN-05), Project of Famous Doctor Inheritance Workstation of Zhang Daizhao,"3+3" Project of Beijing Traditional Chinese Medicine Inheritance (Grant No. 2007-SZ-C-12).

Competing interests None declared.

Patient and public involvement Patients and/or the public were not involved in the design, or conduct, or reporting, or dissemination plans of this research.

Patient consent for publication Not applicable.

Ethics approval Not applicable.

Provenance and peer review: There are five preferred reviewers as listed for your consideration:

For peer review only - http://bmjopen.bmj.com/site/about/guidelines.xhtml

Jinhong Zhu, jinhongzhu625@gmail.com, Department of Clinical Laboratory, Harbin Medical University Cancer Hospital

Yue Qi, qiyue_bjcn@163.com, Department of Epidemiology, Beijing An Zhen Hospital

Jing He, hejing198374@gmail.com, Women and Children's Medical Center, Guangzhou Medical University

Xiaoqun Dong, Xiaoqun_Dong@Brown.edu, The Warren Alpert Medical School, Brown University

Jian Gu, jiangu@mdanderson.org, Department of Epidemiology, The University of Texas MD Anderson Cancer Center

Data availability statement All data relevant to the study are included in the article or uploaded as supplementary information.

Supplemental material This content has been supplied by the author(s). It has not been vetted by BMJ Publishing Group Limited (BMJ) and may not have been peer-reviewed. Any opinions or recommendations discussed are solely those of the author(s) and are not endorsed by BMJ. BMJ disclaims all liability and responsibility arising from any reliance placed on the content. Where the content includes any translated material, BMJ does not warrant the accuracy and reliability of the translations (including but not limited to local regulations, clinical guidelines, terminology, drug names and drug dosages), and is not responsible for any error and/or omissions arising from translation and adaptation or otherwise.

Open access This is an open access article distributed in accordance with the Creative Commons Attribution Non Commercial (CC BY-NC 4.0) license, which permits others to distribute, remix, adapt, build upon this work non-commercially, and license their derivative works on different terms, provided the original work is properly cited, appropriate credit is given, any changes made indicated, and the use is non-commercial. See: http://creativecommons.org/licenses/by-nc/4.0/.

REFERENCES

1 Camilleri M, Chedid V, Ford A, *et al*. Gastroparesis. *Nat Rev Dis Primers* 2018;4:41.

2 Ye Y, Yin Y, Huh S, *et al*. Epidemiology, Etiology, and Treatment of Gastroparesis: Real-World Evidence From a Large US National Claims Database. *Gastroenterology* 2022;162:109-21.e5.

3 Schol J, Wauters L, Dickman R, *et al*. United European Gastroenterology (UEG) and European Society for Neurogastroenterology and Motility (ESNM) consensus on gastroparesis. *United European Gastroenterol J* 2021;9:287-306.

4 Dong K, Yu X, Li B, *et al*. Advances in mechanisms of postsurgical gastroparesis syndrome and its diagnosis and treatment. *Chin J Dig Dis* 2006;7:76-82.

5 Lu D, Altieri M, Yang J, *et al*. Investigating rates of reoperation or postsurgical gastroparesis following fundoplication or paraesophageal hernia repair in New York State. *Surg Endosc* 2019;33:2886-94.

6 Stadlhuber RJ, Sherif AE, Mittal SK, *et al*. Mesh complications after prosthetic reinforcement of hiatal closure: a 28-case series. *Surg Endosc* 2009;23:1219-26.

For peer review only - http://bmjopen.bmj.com/site/about/guidelines.xhtml

7 Anderson MJ, Sippey M, Marks J. Gastric Per Oral Pyloromyotomy for Post-Vagotomy-Induced Gastroparesis Following Esophagectomy. *J Gastrointest Surg* 2020;24:715-19.

8 Hooft N, Smith M, Huang J, *et al*. Gastroparesis is common after lung transplantation and may be ameliorated by botulinum toxin-A injection of the pylorus. *J Heart Lung Transplant* 2014;33:1314-6.

9 Grass F, Schäfer M, Cristaudi A, *et al*. Incidence and Risk Factors of Abdominal Complications After Lung Transplantation. *World J Surg* 2015;39:2274-81.

10 Paul S, Escareno CE, Clancy K, *et al*. Gastrointestinal complications after lung transplantation. *J Heart Lung Transplant* 2009;28:475-9.

11 Chen F, Nakamoto Y, Kondo T, *et al*. Gastroparesis after living-donor lobar lung transplantation: report of five cases. *Surg Today* 2015;45:378-82.

12 Rodriguez J, Strong A, Haskins I, *et al*. Per-oral Pyloromyotomy (POP) for Medically Refractory Gastroparesis: Short Term Results From the First 100 Patients at a High Volume Center. *Ann Surg* 2018;268:421-30.

13 Xu J, Chen T, Elkholy S, *et al*. Gastric Peroral Endoscopic Myotomy (G-POEM) as a Treatment for Refractory Gastroparesis: Long-Term Outcomes. *Can J Gastroenterol Hepatol* 2018;2018:6409698.

14 Burlen J, Runnels M, Mehta M, *et al*. Efficacy of Gastric Electrical Stimulation for Gastroparesis: US/European Comparison. *Gastroenterology Res* 2018;11:349-54.

15 Sun H, Wang DH. Clinical observation on different acupuncture and moxibustion therapies for treatment of postsurgical gastroparesis syndrome. *Zhongguo Zhen Jiu* 2007;27:173-5.

16 Sun B, Luo M, Wu S, *et al*. Acupuncture versus metoclopramide in treatment of postoperative gastroparesis syndrome in abdominal surgical patients: a randomized controlled trial. *Zhong Xi Yi Jie He Xue Bao* 2010;8:641-4.

17 Ernst E. Acupuncture--a critical analysis. *J Intern Med* 2006;259:125-37.

18 Yu B, Sun M, Wang Z, *et al*. Effects of Stimulating Local and Distal Acupoints on Diabetic Gastroparesis: A New Insight in Revealing Acupuncture Therapeutics. *Am J Chin Med* 2021;49:1151-64.

19 Yang N, Yang J, Ye Y, *et al*. Electroacupuncture ameliorates intestinal inflammation by activating α7nAChR-mediated JAK2/STAT3 signaling pathway in postoperative ileus. *Theranostics* 2021;11:4078-89.

20 Noguchi E. Acupuncture regulates gut motility and secretion via nerve reflexes. *Auton Neurosci* 2010;156:15-8.

21 Song Y, Wang X, Li X, *et al*. Effect of Tiaoli Piwei needling technique on diabetic gastroparesis and transmembrane protein 16A. *Zhongguo Zhen Jiu* 2020;40:811-5.

22 Zhang C, Huang X, Luo Y, *et al*. Observation on therapeutic effects of acupoint injection of metoclopramide for postsurgical gastroparesis syndrome. *Zhen Ci Yan Jiu* 2014;39:406-9.

23 Chen K, Wu Z, Wang J, *et al*. Efficacy and safety of long-term transcutaneous electroacupuncture versus sham transcutaneous electroacupuncture for delayed gastric emptying after distal gastrectomy: study protocol for a randomized, patient-assessor blinded, controlled trial. *Trials* 2022;23:189.

For peer review only - http://bmjopen.bmj.com/site/about/guidelines.xhtml

24 Qiu Y, Zhang J, Niu S. Clinical Effect of Acupuncture of 'Tong Fu Chu Man Method' Combinng Xiao Cheng Qi Decoction on Gastric Palsy Syndrome after Abdominal Surgery and Its Effect on Gastrointestinal Dynamics (In Chinese). *Xinjiang Journal of Traditional Chinese Medicine* 2020;38:20-23.

25 Page M, McKenzie J, Bossuyt P, *et al*. The PRISMA 2020 statement: an updated guideline for reporting systematic reviews. *BMJ* 2021;372:n71.

26 Qin X, Liu F. Diagnosis and treatment of postoperative gastroparesis (In Chinese). *Chinese Journal of Digestion* 2005:441-42.

27 Camilleri M, Parkman H, Shafi M, *et al*. Clinical guideline: management of gastroparesis. *Am J Gastroenterol* 2013;108:18-37; quiz 38.

28 Grover M, Farrugia G, Stanghellini V. Gastroparesis: a turning point in understanding and treatment. *Gut* 2019;68:2238-50.

29 Cumpston M, Li T, Page M, *et al*. Updated guidance for trusted systematic reviews: a new edition of the Cochrane Handbook for Systematic Reviews of Interventions. *Cochrane Database Syst Rev* 2019;10:ED000142.

30 Guyatt GH, Oxman AD, Kunz R, *et al*. What is "quality of evidence" and why is it important to clinicians? *BMJ* 2008;336:995-8.

31 Guyatt G, Oxman AD, Akl EA, *et al*. GRADE guidelines: 1. Introduction-GRADE evidence profiles and summary of findings tables. *J Clin Epidemiol* 2011;64:383-94.

32 Chen Z, Xu F. Clinical study on the treatment of postoperative gastroparesis syndrome by acupuncture 'Three needle in stomach' (In Chinese). *Shandong Journal of Traditional Chinese Medicine* 2012;31:496-97.

33 Chen J, He P, Zhao C, *et al*. Therapeutic Observation of Acupuncture-moxibustion for Postsurgical Gastroparesis and Effect on Gastrin and Motilin (In Chinese). *Shanghai Journal of Acupuncture and Moxibustion* 2016;35:1083-86.

34 Dong W, Run Y, Zhao Z, *et al*. Clinical effect of warm acupuncture combined with enteral nutrition on postsurgical gastroparesis syndrome (In Chinese). *Global Traditional Chinese Medicine* 2017;10:1134-36.

35 Huang J, Chen C, Tan C, *et al*. Effect of Acupuncture at Zusanli (ST36) for Gastroparesis Syndrome after Radical Subtotal Gastrectomy. *Shanghai Journal of Acupuncture and Moxibustion* 2020;39:1429-33.

36 Li H, Shen H. Clinical Study on the Treatment of Postsurgical Gastroparesis Syndrome by Gastrointestinal Xia He Point (In Chinese). *Shanghai Journal of Acupuncture and Moxibustion* 2005:14-16.

37 Lin P. Clinical study on the treatment of Gastroparesis Syndrome after abdominal operation with acupuncture of "Tong Fu Chu Man" (In Chinese). *Fujian University of Traditional Chinese Medicine*, 2017.

38 Liu X. Clinical observation of electroacupuncture combined with Western medicine in the treatment of gastroparalysis (In Chinese). *Health for Everyone* 2017:75-76.

39 Ma L, Zhang C, Shen Y. Acupuncture and moxibustion combined with enteral nutrition in the treatment of postsurgical gastroparesis syndrome (In Chinese). *Zhejiang Journal of Traditional Chinese Medicine* 2014;49:672.

For peer review only

40 Meng Z, Dou L, Du G, *et al*. Therapeutic Observation on Electroacupuncture for Gastroparesis after Subtotal Gastrectomy (In Chinese). *Shanghai Journal of Acupuncture and Moxibustion* 2012;31:321-22.

41 Sun Y, Sun Y. Acupuncture with Mosapride for postoperative thoracic gastric emptying disorder in esophageal cancer (In Chinese). *Journal of Hebei Traditional Chinese Medicine and Pharmacology* 2010;25:42-43.

42 Yang Z, Hou Z, Gong D, *et al*. Clinical evaluation of electroacupuncture for functional gastric emptying disorder after abdominal surgery (In Chinese). *Journal of Hebei Traditional Chinese Medicine and Pharmacology* 2010;25:32-33.

43 Zhang L, Guan L, Zhang Y, *et al*. Clinical observation of Dongyuan acupuncture combined with mosapride citrate for treatment of gastroparesis after gynecological abdominal surgery (In Chinese). *Journal of Guangzhou University of Traditional Chinese Medicine* 2021;38:1645-50.

44 Zhang J, Zhao J, Jiao B, *et al*. Clinical observation on the treatment of postoperative gastroparesis of gastric cancer by acupuncture combined with conventional western medicine (In Chinese). *China's Naturopathy* 2022;30:64-66.

45 Zhao W, Jiang X, Huang J. Clinical observation on the effects of warm acupuncture and moxinbustion on gastroparesis after tumor operation (In Chinese). *China Journal of Traditional Chinese Medicine and Pharmacy* 2021;36:4379-82.

46 Wang Y, Yang H, Liu Y, *et al*. Acupuncture in the Treatment of Postsurgical Gastro-paresis Syndrome: Clinical Observation of 22 cases (In Chinese). *Journal of Clinical Acupuncture and Moxibustion* 2013;29:10-13.

47 Chen HY, Kuo TB, Shaw FZ, *et al*. Sleep-related vagotonic effect of zolpidem in rats. *Psychopharmacology* 2005;181:270-9.

48 Rao A, Camilleri M. Review article: metoclopramide and tardive dyskinesia. *Aliment Pharmacol Ther* 2010;31:11-9.

49 Cullen PT, Campbell FC. Cisapride in postoperative gastroparesis. *Lancet* 1987;1:47.

50 Berkowitz N, Schulman LL, McGregor C, *et al*. Gastroparesis after lung transplantation. Potential role in postoperative respiratory complications. *Chest* 1995;108:1602-7.

51 Kawamura E, Enomoto M, Kotani K, *et al*. Effect of mosapride citrate on gastric emptying in interferon-induced gastroparesis. *Dig Dis Sci* 2012;57:1510-6.

52 Ogasawara N, Yamaguchi Y, Takahama T, *et al*. Radiofrequency catheter ablation-induced gastroparesis successfully treated with administration of mosapride citrate: two case reports. *Clin J Gastroenterol* 2020;13:182-85.

53 Carbone F, Van den Houte K, Clevers E, *et al*. Prucalopride in Gastroparesis: A Randomized Placebo-Controlled Crossover Study. *Am J Gastroenterol* 2019;114:1265-74.

54 Richards RD, Davenport K, McCallum RW. The treatment of idiopathic and diabetic gastroparesis with acute intravenous and chronic oral erythromycin. *Am J Gastroenterol* 1993;88:203-7.

55 Talley NJ, Verlinden M, Snape W, *et al*. Failure of a motilin receptor agonist (ABT-229) to relieve the symptoms of functional dyspepsia in patients with and without delayed gastric

For peer review only

emptying: a randomized double-blind placebo-controlled trial. *Aliment Pharmacol Ther* 2000;14:1653-61.

56 Garg R, Mohan BP, Aggarwal M, *et al*. Peroral Pyloromytomy is Effective and Safe for Postsurgical Gastroparesis. *J Gastrointest Surg* 2020;24:1417-20.

57 Takahashi T. Acupuncture for functional gastrointestinal disorders. *J Gastroenterol* 2006;41:408-17.

58 Yin J, Chen J, Chen J. Ameliorating effects and mechanisms of electroacupuncture on gastric dysrhythmia, delayed emptying, and impaired accommodation in diabetic rats. *Am J Physiol Gastrointest Liver Physiol* 2010;298:G563-70.

59 Lu M, Yu Z, He Y, *et al*. Electroacupuncture at ST36 modulates gastric motility via vagovagal and sympathetic reflexes in rats. *World J Gastroenterol* 2019;25:2315-26.

60 Yue G, Yuan X, Ge X, *et al*. Effect of electroacupuncture at different acupoints in treating rapid gastric emptying: Zusanli (ST36) plus Zhongwan (CV12) versus Zhongwan (CV12) alone. *J Tradit Chin Med* 2021;41:326-30.

61 Cui Y, Wang L, Shi G, *et al*. Electroacupuncture alleviates cisplatin-induced nausea in rats. *Acupunct Med* 2016;34:120-6.

62 Garcia G, Ribeiro R, Faria Santos I, *et al*. Electrical Stimulation of PC 6 to Control Chemotherapy-Induced Nausea and Vomiting in Patients with Cancer: A Systematic Review and Meta-Analysis. *Med Acupunct* 2021;33:22-44.

63 Zhang Y, Zhang C, Yan M, *et al*. The effectiveness of PC6 acupuncture in the prevention of postoperative nausea and vomiting in children: A systematic review and meta-analysis. *Paediatr Anaesth* 2020;30:552-63.

64 Stott A. Examining the efficacy of stimulating the PC6 wrist acupuncture point for preventing postoperative nausea and vomiting: A Cochrane review summary. *Int J Nurs Stud* 2016;64:139-41.

65 Lu M, Chen C, Li W, *et al*. EA at PC6 Promotes Gastric Motility: Role of Brainstem Vagovagal Neurocircuits. *Evid Based Complement Alternat Med* 2019;2019:7457485.

66 Gao L, Chen B, Zhang Q, *et al*. Acupuncture with different acupoint combinations for chemotherapy-induced nausea and vomiting: study protocol for a randomized controlled trial. *BMC Complement Altern Med* 2016;16:441.

Legends:

Figure 1 PRISMA flow diagram of screening process.

Figure 2 Risk of bias summary and risk of bias graph.

Figure 3 Forest plot of total effective rate (A) and recovery rate (B).

Figure 4 Forest plot of serum motilin (A), serum gastrin (B) and gastric juice volume (C).

Figure 5 Forest plot of days of gastrointestinal motility recovery(A), days of gastrointestinal decompression(B), days of resume diet for the first time(C) and clinical symptom score(D).

For peer review only

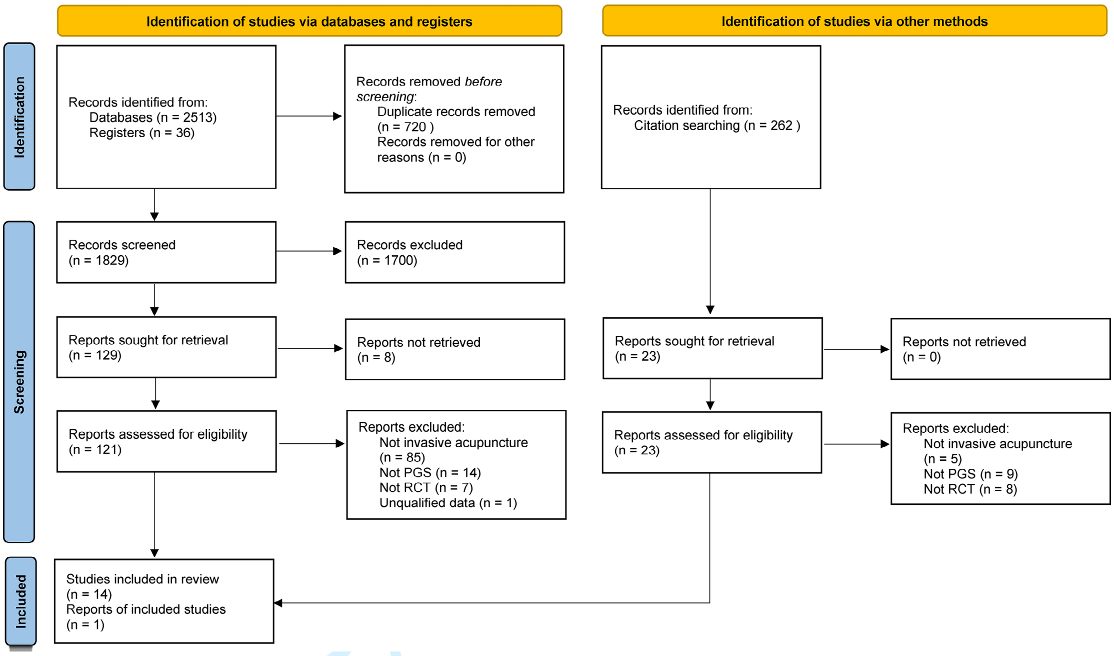

Figure 1 PRISMA flow diagram of screening process.

For peer review only

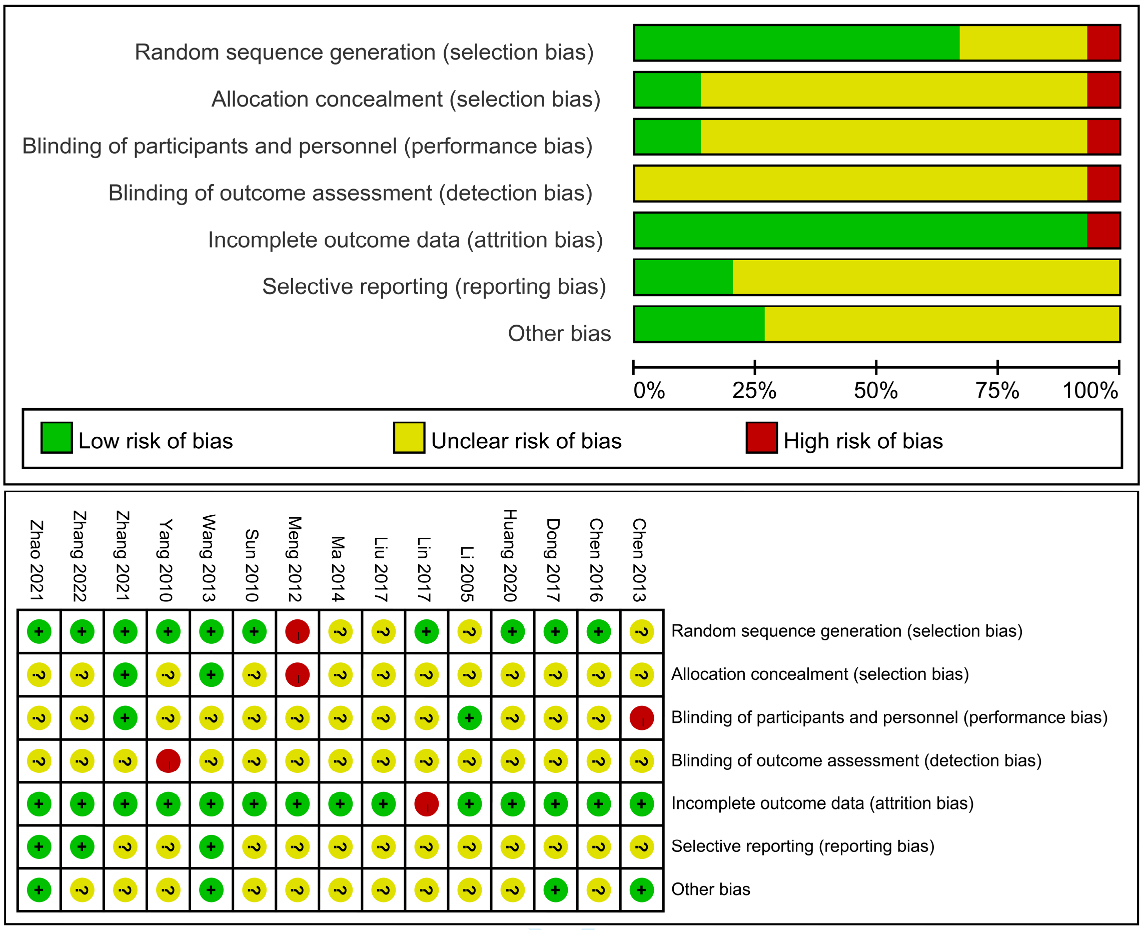

Figure 2 Risk of bias summary and risk of bias graph.

view only

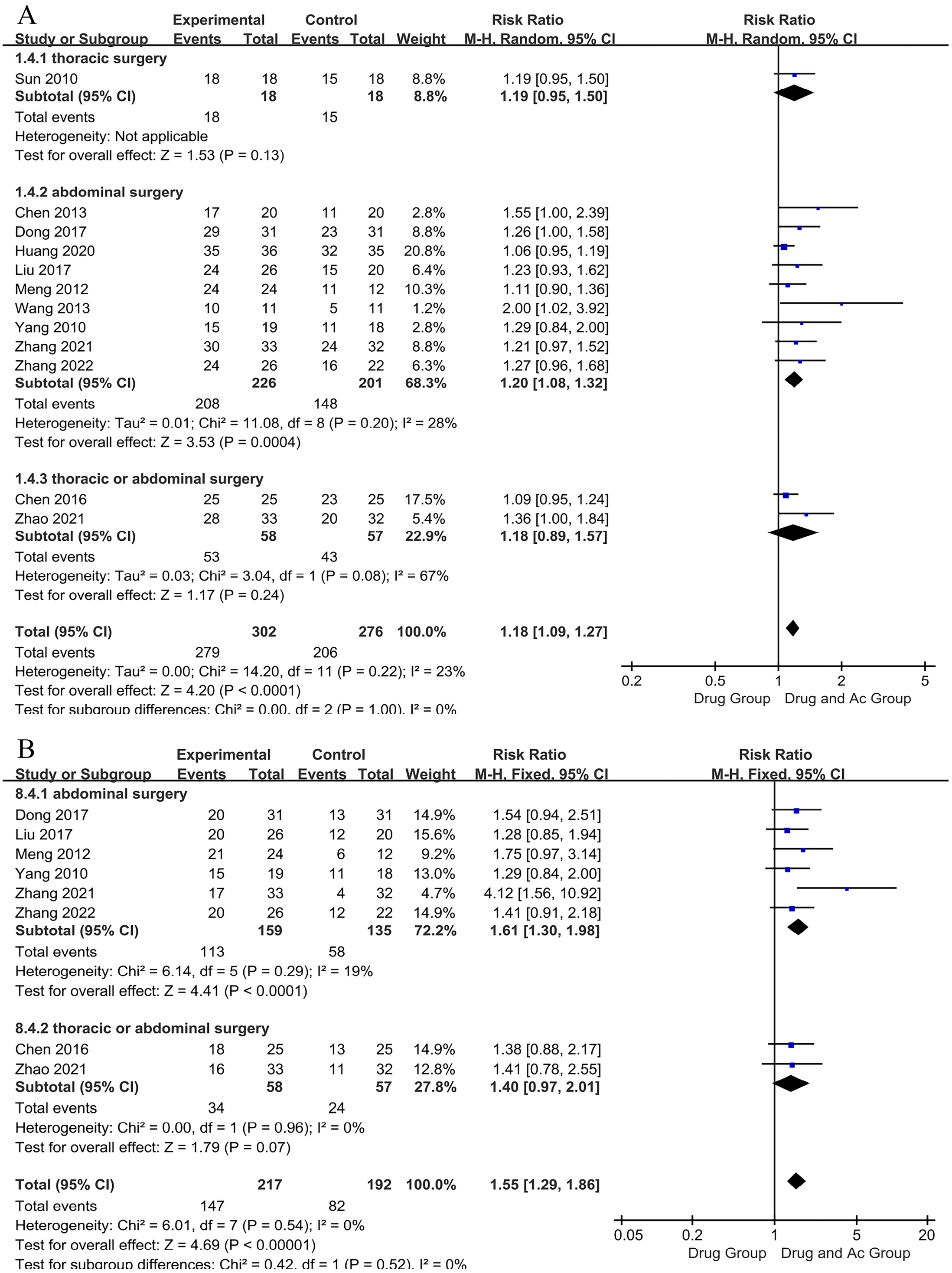

Figure 3 Forest plot of total effective rate (A) and recovery rate (B).

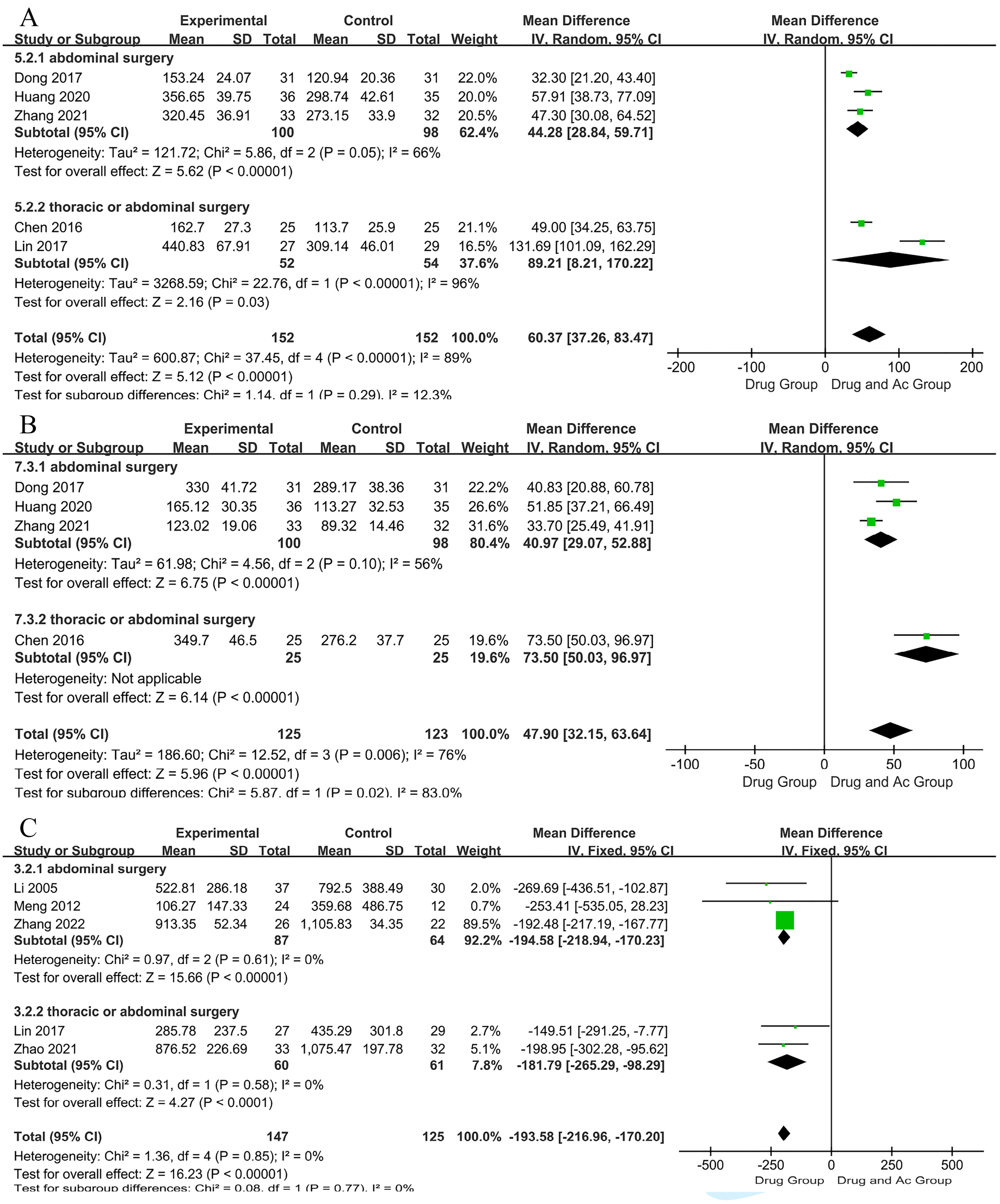

Figure 4 Forest plot of serum motilin (A), serum gastrin (B) and gastric juice volume (C).

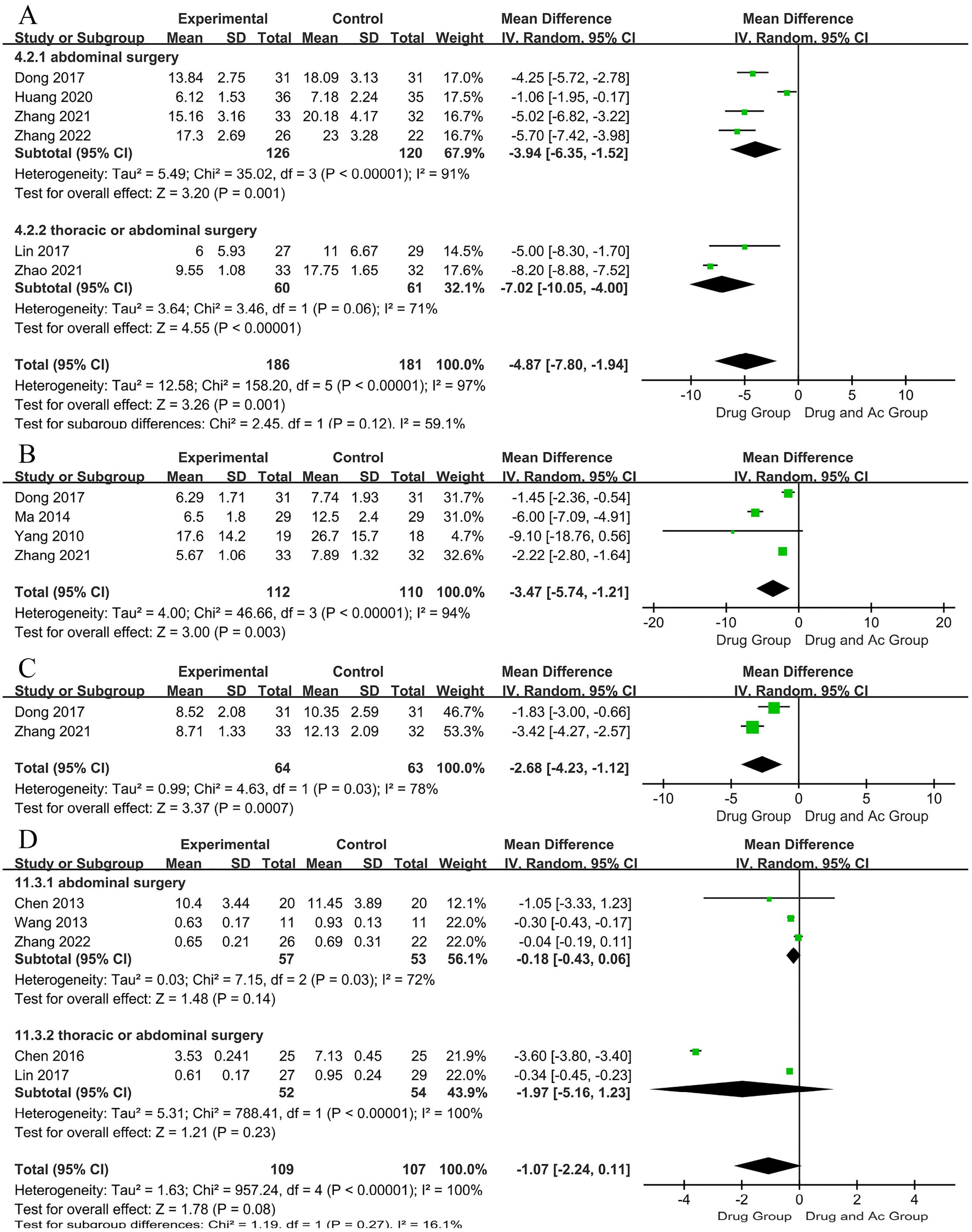

Figure 5 Forest plot of days of gastrointestinal motility recovery(A), days of gastrointestinal decompression(B), days of resume diet for the first time(C) and clinical symptom score(D).

Supplemental Figures

A

Study or Subgroup	Experimental Events	Experimental Total	Control Events	Control Total	Weight	Risk Ratio M-H, Fixed, 95% CI
**1.2.1 course≤14 days**						
Chen 2013	17	20	11	20	5.1%	1.55 [1.00, 2.39]
Chen 2016	25	25	23	25	10.9%	1.09 [0.95, 1.24]
Huang 2020	35	36	32	35	15.0%	1.06 [0.95, 1.19]
Meng 2012	24	24	11	12	7.0%	1.11 [0.90, 1.36]
Zhang 2021	30	33	24	32	11.3%	1.21 [0.97, 1.52]
Zhang 2022	24	26	16	22	8.0%	1.27 [0.96, 1.68]
Zhao 2021	28	33	20	32	9.4%	1.36 [1.00, 1.84]
**Subtotal (95% CI)**		**197**		**178**	**66.8%**	**1.20 [1.10, 1.31]**
Total events	183		137			
Heterogeneity: Chi² = 8.95, df = 6 (P = 0.18); I² = 33%						
Test for overall effect: Z = 4.07 (P < 0.0001)						
**1.2.2 course＞14 days**						
Liu 2017	24	26	15	20	7.9%	1.23 [0.93, 1.62]
Wang 2013	10	11	5	11	2.3%	2.00 [1.02, 3.92]
Yang 2010	15	19	11	18	5.2%	1.29 [0.84, 2.00]
**Subtotal (95% CI)**		**56**		**49**	**15.4%**	**1.37 [1.08, 1.72]**
Total events	49		31			
Heterogeneity: Chi² = 1.85, df = 2 (P = 0.40); I² = 0%						
Test for overall effect: Z = 2.65 (P = 0.008)						
**1.2.3 course unclear**						
Dong 2017	29	31	23	31	10.7%	1.26 [1.00, 1.58]
Sun 2010	18	18	15	18	7.2%	1.19 [0.95, 1.50]
**Subtotal (95% CI)**		**49**		**49**	**17.8%**	**1.23 [1.05, 1.45]**
Total events	47		38			
Heterogeneity: Chi² = 0.12, df = 1 (P = 0.73); I² = 0%						
Test for overall effect: Z = 2.51 (P = 0.01)						
**Total (95% CI)**		**302**		**276**	**100.0%**	**1.23 [1.14, 1.33]**
Total events	279		206			
Heterogeneity: Chi² = 14.20, df = 11 (P = 0.22); I² = 23%						
Test for overall effect: Z = 5.46 (P < 0.00001)						
Test for subgroup differences: Chi² = 1.09, df = 2 (P = 0.58), I² = 0%						

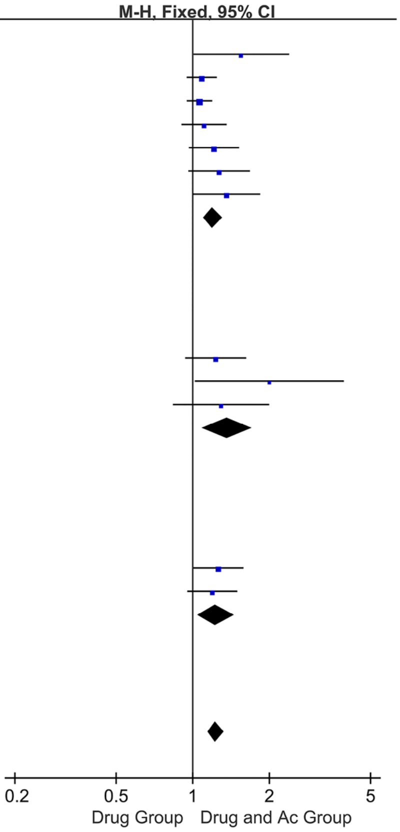

B

Study or Subgroup	Experimental Events	Experimental Total	Control Events	Control Total	Weight	Risk Ratio M-H, Fixed, 95% CI
**1.3.1 Drug and MAc**						
Chen 2013	17	20	11	20	5.1%	1.55 [1.00, 2.39]
Huang 2020	35	36	32	35	15.0%	1.06 [0.95, 1.19]
Sun 2010	18	18	15	18	7.2%	1.19 [0.95, 1.50]
Zhang 2021	30	33	24	32	11.3%	1.21 [0.97, 1.52]
Zhang 2022	24	26	16	22	8.0%	1.27 [0.96, 1.68]
**Subtotal (95% CI)**		**133**		**127**	**46.6%**	**1.21 [1.09, 1.34]**
Total events	124		98			
Heterogeneity: Chi² = 6.01, df = 4 (P = 0.20); I² = 33%						
Test for overall effect: Z = 3.56 (P = 0.0004)						
**1.3.2 Drug and SMAc**						
Chen 2016	25	25	23	25	10.9%	1.09 [0.95, 1.24]
Dong 2017	29	31	23	31	10.7%	1.26 [1.00, 1.58]
Liu 2017	24	26	15	20	7.9%	1.23 [0.93, 1.62]
Meng 2012	24	24	11	12	7.0%	1.11 [0.90, 1.36]
Wang 2013	10	11	5	11	2.3%	2.00 [1.02, 3.92]
Yang 2010	15	19	11	18	5.2%	1.29 [0.84, 2.00]
Zhao 2021	28	33	20	32	9.4%	1.36 [1.00, 1.84]
**Subtotal (95% CI)**		**169**		**149**	**53.4%**	**1.25 [1.13, 1.39]**
Total events	155		108			
Heterogeneity: Chi² = 7.80, df = 6 (P = 0.25); I² = 23%						
Test for overall effect: Z = 4.14 (P < 0.0001)						
**Total (95% CI)**		**302**		**276**	**100.0%**	**1.23 [1.14, 1.33]**
Total events	279		206			
Heterogeneity: Chi² = 14.20, df = 11 (P = 0.22); I² = 23%						
Test for overall effect: Z = 5.46 (P < 0.00001)						
Test for subgroup differences: Chi² = 0.23, df = 1 (P = 0.63), I² = 0%						

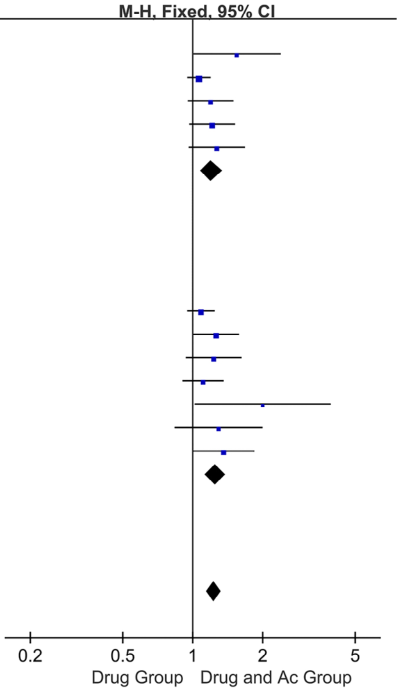

Supplemental Figure 1 Forest plots for subgroup analysis of acupuncture course(A) and acupuncture methods(B) for total effective rate.

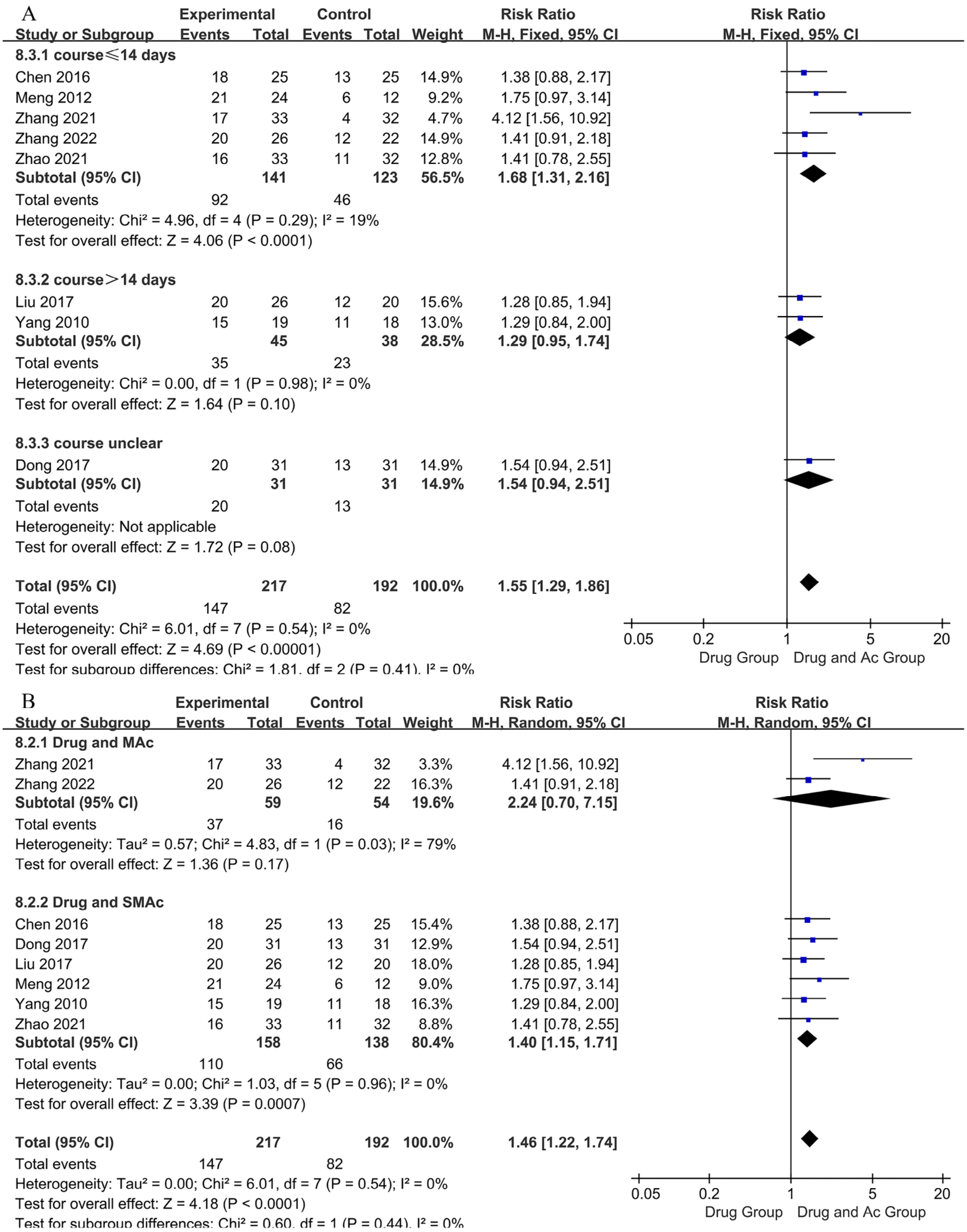

Supplemental Figure 2 Forest plots for subgroup analysis of acupuncture course(A) and acupuncture methods(B) for cure rate.

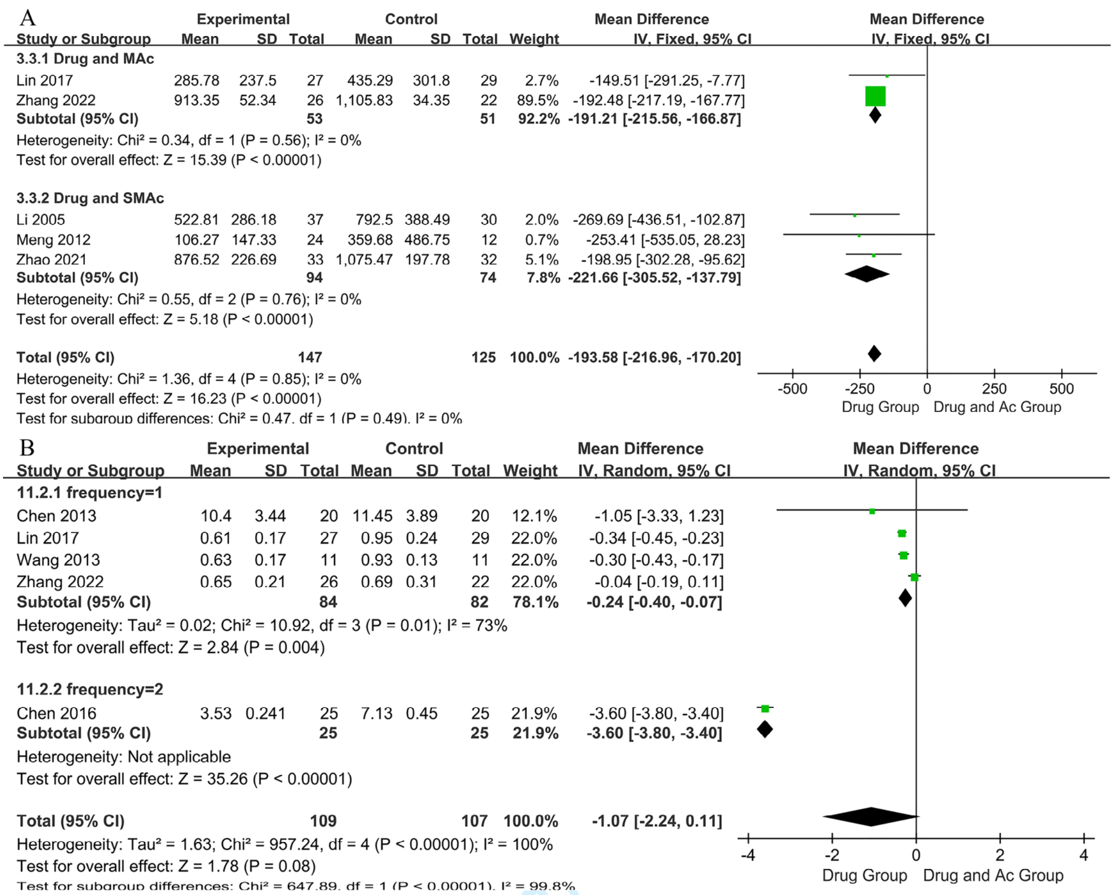

Supplemental Figure 3 Forest plot for subgroup analysis of acupuncture methods for gastric juice volume(A), and subgroup analysis of acupuncture frequency for clinical symptom score(B).

For peer review only - http://bmjopen.bmj.com/site/about/guidelines.xhtml

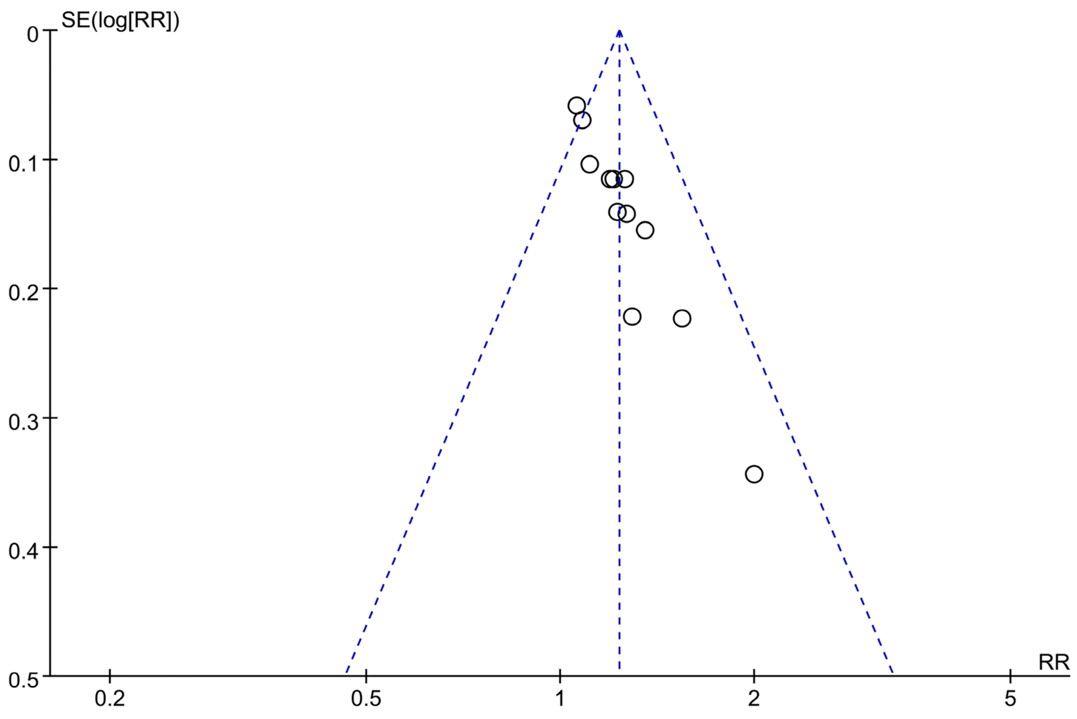

Supplemental Figure 4 Funnel plot of the total effective rate.

For peer review only - http://bmjopen.bmj.com/site/about/guidelines.xhtml

PRISMA 2020 Checklist

Section and Topic	Item #	Checklist item	Location where item is reported
**TITLE**			
Title	1	Identify the report as a systematic review.	1
**ABSTRACT**			
Abstract	2	See the PRISMA 2020 for Abstracts checklist.	1
**INTRODUCTION**			
Rationale	3	Describe the rationale for the review in the context of existing knowledge.	2
Objectives	4	Provide an explicit statement of the objective(s) or question(s) the review addresses.	3
**METHODS**			
Eligibility criteria	5	Specify the inclusion and exclusion criteria for the review and how studies were grouped for the syntheses.	3
Information sources	6	Specify all databases, registers, websites, organisations, reference lists and other sources searched or consulted to identify studies. Specify the date when each source was last searched or consulted.	3
Search strategy	7	Present the full search strategies for all databases, registers and websites, including any filters and limits used.	3
Selection process	8	Specify the methods used to decide whether a study met the inclusion criteria of the review, including how many reviewers screened each record and each report retrieved, whether they worked independently, and if applicable, details of automation tools used in the process.	3
Data collection process	9	Specify the methods used to collect data from reports, including how many reviewers collected data from each report, whether they worked independently, any processes for obtaining or confirming data from study investigators, and if applicable, details of automation tools used in the process.	4
Data items	10a	List and define all outcomes for which data were sought. Specify whether all results that were compatible with each outcome domain in each study were sought (e.g. for all measures, time points, analyses), and if not, the methods used to decide which results to collect.	4
	10b	List and define all other variables for which data were sought (e.g. participant and intervention characteristics, funding sources). Describe any assumptions made about any missing or unclear information.	4
Study risk of bias assessment	11	Specify the methods used to assess risk of bias in the included studies, including details of the tool(s) used, how many reviewers assessed each study and whether they worked independently, and if applicable, details of automation tools used in the process.	4
Effect measures	12	Specify for each outcome the effect measure(s) (e.g. risk ratio, mean difference) used in the synthesis or presentation of results.	4
Synthesis methods	13a	Describe the processes used to decide which studies were eligible for each synthesis (e.g. tabulating the study intervention characteristics and comparing against the planned groups for each synthesis (item #5)).	4
	13b	Describe any methods required to prepare the data for presentation or synthesis, such as handling of missing summary statistics, or data conversions.	4
	13c	Describe any methods used to tabulate or visually display results of individual studies and syntheses.	4
	13d	Describe any methods used to synthesize results and provide a rationale for the choice(s). If meta-analysis was performed, describe the model(s), method(s) to identify the presence and extent of statistical heterogeneity, and software package(s) used.	4
	13e	Describe any methods used to explore possible causes of heterogeneity among study results (e.g. subgroup analysis, meta-regression).	4
	13f	Describe any sensitivity analyses conducted to assess robustness of the synthesized results.	4
Reporting bias assessment	14	Describe any methods used to assess risk of bias due to missing results in a synthesis (arising from reporting biases).	4
Certainty assessment	15	Describe any methods used to assess certainty (or confidence) in the body of evidence for an outcome.	4

For peer review only

PRISMA 2020 Checklist

Section and Topic	Item #	Checklist item	Location where item is reported
**RESULTS**			
Study selection	16a	Describe the results of the search and selection process, from the number of records identified in the search to the number of studies included in the review, ideally using a flow diagram.	5
	16b	Cite studies that might appear to meet the inclusion criteria, but which were excluded, and explain why they were excluded.	5
Study characteristics	17	Cite each included study and present its characteristics.	5
Risk of bias in studies	18	Present assessments of risk of bias for each included study.	5
Results of individual studies	19	For all outcomes, present, for each study: (a) summary statistics for each group (where appropriate) and (b) an effect estimate and its precision (e.g. confidence/credible interval), ideally using structured tables or plots.	6-8
Results of syntheses	20a	For each synthesis, briefly summarise the characteristics and risk of bias among contributing studies.	6-8
	20b	Present results of all statistical syntheses conducted. If meta-analysis was done, present for each the summary estimate and its precision (e.g. confidence/credible interval) and measures of statistical heterogeneity. If comparing groups, describe the direction of the effect.	6-8
	20c	Present results of all investigations of possible causes of heterogeneity among study results.	6-8
	20d	Present results of all sensitivity analyses conducted to assess the robustness of the synthesized results.	6-8
Reporting biases	21	Present assessments of risk of bias due to missing results (arising from reporting biases) for each synthesis assessed.	8
Certainty of evidence	22	Present assessments of certainty (or confidence) in the body of evidence for each outcome assessed.	5
**DISCUSSION**			
Discussion	23a	Provide a general interpretation of the results in the context of other evidence.	9
	23b	Discuss any limitations of the evidence included in the review.	10-11
	23c	Discuss any limitations of the review processes used.	10-11
	23d	Discuss implications of the results for practice, policy, and future research.	11
**OTHER INFORMATION**			
Registration and protocol	24a	Provide registration information for the review, including register name and registration number, or state that the review was not registered.	3
	24b	Indicate where the review protocol can be accessed, or state that a protocol was not prepared.	3
	24c	Describe and explain any amendments to information provided at registration or in the protocol.	NA
Support	25	Describe sources of financial or non-financial support for the review, and the role of the funders or sponsors in the review.	11
Competing interests	26	Declare any competing interests of review authors.	11
Availability of data, code and other materials	27	Report which of the following are publicly available and where they can be found: template data collection forms; data extracted from included studies; data used for all analyses; analytic code; any other materials used in the review.	12

From: Page MJ, McKenzie JE, Bossuyt PM, Boutron I, Hoffmann TC, Mulrow CD, et al. The PRISMA 2020 statement: an updated guideline for reporting systematic reviews. BMJ 2021;372:n71. doi: 10.1136/bmj.n71

For peer review only - http://bmjopen.bmj.com/site/about/guidelines.xhtml

For more information, visit: http://www.prisma-statement.org/

For peer review only

Supplemental Table 2 Search strategy

Database	#	Search syntax
PubMed	1	(acupuncture[MeSH Terms]) OR (acupuncture therapy[MeSH Terms]) OR (acupoint[MeSH Terms]) OR (electroacupuncture[MeSH Terms]) OR (electric acupuncture[MeSH Terms]) OR (auriculotherapy[MeSH Terms]) OR (auricular acupuncture[MeSH Terms]) OR (elongated needle[MeSH Terms]) OR (scalp acupuncture[MeSH Terms]) OR (hand acupuncture[MeSH Terms]) OR (manual acupuncture[MeSH Terms]) OR (body acupuncture[MeSH Terms]) OR (warm* acupuncture[MeSH Terms]) OR (warm* needl*[MeSH Terms]) OR (fire needl*[MeSH Terms]) OR (trigger point[MeSH Terms]) OR (meridian[MeSH Terms]) OR (needl*[MeSH Terms]) OR (abdominal acupuncture[MeSH Terms]) OR (float* needl*[MeSH Terms]) OR (embedded acupuncture[MeSH Terms]) OR (intradermal acupuncture[MeSH Terms]) OR (needl*[MeSH Terms]) OR (acupuncture*[Title/Abstract]) OR (acupuncture therapy[Title/Abstract]) OR (acupoint*[Title/Abstract]) OR (electroacupuncture*[Title/Abstract]) OR (electric acupuncture*[Title/Abstract]) OR (auriculotherapy[Title/Abstract]) OR (auricular acupuncture*[Title/Abstract]) OR (elongated needl*[Title/Abstract]) OR (scalp acupuncture*[Title/Abstract]) OR (hand acupuncture*[Title/Abstract]) OR (manual acupuncture*[Title/Abstract]) OR (body acupuncture*[Title/Abstract]) OR (warm* acupuncture*[Title/Abstract]) OR (warm* needl*[Title/Abstract]) OR (fire needl*[Title/Abstract]) OR (trigger point*[Title/Abstract]) OR (meridian*[Title/Abstract]) OR (needl*[Title/Abstract]) OR (abdominal acupuncture*[Title/Abstract]) OR (float* needl*[Title/Abstract]) OR (embedded acupuncture*[Title/Abstract]) OR (intradermal acupuncture*[Title/Abstract]) OR (electro-acupuncture*[Title/Abstract]) OR (micro-puncture*[Title/Abstract]) OR (needl* embedding[Title/Abstract])
	2	(surgery[MeSH Terms]) OR (operation[MeSH Terms]) OR (postoperative[MeSH Terms]) OR (surger*[Title/Abstract]) OR (operation*[Title/Abstract]) OR (postoperati*[Title/Abstract]) OR (postsurgical[Title/Abstract]) OR (excision*[Title/Abstract]) OR (resecti*[Title/Abstract])
	3	(gastroparesis[MeSH Terms]) OR (gastropareses[MeSH Terms]) OR (gastric stasis[MeSH Terms]) OR (gastric stases[MeSH Terms]) OR (stasis, gastric[MeSH Terms]) OR (gastr* paralysis[MeSH Terms]) OR (delay* gastric emptying[MeSH Terms]) OR (stomach palsy[MeSH Terms]) OR (gastr* motility disorder[MeSH Terms]) OR (gastroparesis[Title/Abstract]) OR (stasis, gastric[Title/Abstract]) OR (gastr* paralysis[Title/Abstract]) OR (delay* gastric emptying[Title/Abstract]) OR (stomach palsy[Title/Abstract]) OR (gastr* motility disorder[Title/Abstract]) OR (gastric stasis[Title/Abstract]) OR (gastroparesis syndrome[Title/Abstract]) OR (impaired gastric accommodation[Title/Abstract]) OR (stomach paresis[Title/Abstract])
	4	(Randomized controlled trial) or ((controlled) and (study or design or trial)) or (random*) or (intermethod comparison) or (placebo) or (compar*) or ((singl* or doubl* or trebl* or tripl*) and (blind* or mask*)) or (cross over) or (trial[Title/Abstract])
	5	#1 AND #2 AND #3 AND #4
Embase	1	'acupuncture'/exp OR 'needle'/exp OR 'electroacupuncture'/exp OR 'auricular acupuncture'/exp OR 'acupuncture needle'/exp OR 'scalp acupuncture'/exp OR 'manual acupuncture'/exp OR 'body acupuncture'/exp OR 'warm acupuncture'/exp OR 'meridian'/exp OR 'abdominal acupuncture'/exp OR 'trigger point'/exp OR 'dry needling'/exp OR 'acupuncture*':ti,ab,kw OR 'needl*':ti,ab,kw OR 'electroacupuncture*':ti,ab,kw OR 'auricular acupuncture*':ti,ab,kw OR 'acupuncture* needl*':ti,ab,kw OR 'scalp acupuncture*':ti,ab,kw OR 'manual acupuncture*':ti,ab,kw OR 'body acupuncture*':ti,ab,kw OR 'warm* acupuncture*':ti,ab,kw OR 'meridian*':ti,ab,kw OR 'abdominal acupuncture*':ti,ab,kw OR 'trigger point*':ti,ab,kw OR 'dry needl*':ti,ab,kw OR 'acupuncture* therap*':ti,ab,kw OR 'acupoint*':ti,ab,kw OR 'electric

For peer review only

		acupuncture*':ti,ab,kw OR 'electro-acupuncture*':ti,ab,kw OR 'auricular-acupuncture*':ti,ab,kw OR 'auriculotherap*':ti,ab,kw OR 'elongated needl*':ti,ab,kw OR 'micro-puncture*':ti,ab,kw OR 'hand acupuncture*':ti,ab,kw OR 'warm* needl*':ti,ab,kw OR 'fire needl*':ti,ab,kw OR 'float* needl*':ti,ab,kw OR 'embedd* acupuncture*':ti,ab,kw OR 'needl* embedd*':ti,ab,kw OR 'intradermal acupuncture*':ti,ab,kw
	2	'surgery'/exp OR 'excision'/exp OR 'surger*':ti,ab,kw OR 'excision*':ti,ab,kw OR 'operation*':ti,ab,kw OR 'postoperation*':ti,ab,kw OR 'postsurgical*':ti,ab,kw OR 'postoperative*':ti,ab,kw OR 'resection*':ti,ab,kw
	3	'stomach paresis'/exp OR 'gastric stasis'/exp OR 'stomach emptying'/exp OR 'gastric dysmotility'/exp OR 'stomach paresis':ti,ab,kw OR 'gastric stasis':ti,ab,kw OR 'stomach emptying':ti,ab,kw OR 'gastric dysmotility':ti,ab,kw OR 'gastroparesis':ti,ab,kw OR 'gastropareses':ti,ab,kw OR 'stasis, gastric':ti,ab,kw OR 'gastr* paralysis':ti,ab,kw OR 'gastroparesis syndrome':ti,ab,kw OR 'delay* gastr* empty*':ti,ab,kw OR 'disturbance of gastric emptying':ti,ab,kw OR 'gastr* motility disorder':ti,ab,kw OR 'impaired gastric accommodation':ti,ab,kw OR 'stomach paralysis':ti,ab,kw
	4	'Randomized controlled trial' or 'controlled adj7 (study or design or trial)' or 'random*' or 'intermethod comparison' or 'placebo' or 'compar*' or '(singl* or doubl* or trebl* or tripl*) adj (blind* or mask*)' or '(cross-over or crossover or cross over)' or 'trial':ti,kw,ab
	5	#1 AND #2 AND #3 AND #4
CENTRAL	1	(MeSH descriptor:[Acupuncture OR 'Acupuncture Therapy' OR 'Acupuncture Points' OR 'Needles' OR 'Auriculotherapy' OR 'Acupuncture, Ear' OR 'Electroacupuncture' OR 'Trigger Points' OR 'Meridians' OR 'Dry Needling'] explode all trees) OR (acupuncture* OR needl* OR electroacupuncture* OR 'auricular acupuncture*' OR 'acupuncture* needl*' OR 'scalp acupuncture*' OR 'manual acupuncture*' OR 'body acupuncture*' OR 'warm* acupuncture*' OR meridian* OR 'abdominal acupuncture*' OR 'trigger point*' OR 'dry needl*' OR 'acupuncture* therap*' OR acupoint* OR 'electric acupuncture*' OR electro-acupuncture* OR auricular-acupuncture* OR auriculotherap* OR 'elongated needl*' OR 'micro-puncture*' OR 'hand acupuncture*' OR 'warm* needl*' OR 'fire needl*' OR 'float* needl*' OR 'embedd* acupuncture*' OR 'needl* embedd*' OR 'intradermal acupuncture*'):ti,ab,kw
	2	MeSH descriptor:[General Surgery] explode all trees OR (surger* OR excision* OR operation* OR postoperation* OR postsurgical* OR postoperative* OR resection*):ti,ab,kw
	3	MeSH descriptor:[Gastroparesis] explode all trees OR (gastroparesis OR gastropareses OR gastric stasis OR gastric stases OR 'gastr* paralysis' OR 'delay* gastric emptying' OR 'disturbance of gastric emptying' OR 'weakness of gastric emptying' OR 'weakness of stomach' OR 'stomach palsy' OR 'gastr* motility disorder' OR 'gastroparesis syndrome' OR 'impaired gastric accommodation' OR 'stomach paresis'):ti,ab,kw
	4	#1 AND #2 AND #3
Medline (Ovid) (1946-)	1	exp Acupuncture/ or exp Acupuncture Therapy/ or exp Acupuncture Points/ or exp Needles/ or exp electroacupuncture/ or exp auriculotherapy/ or exp Acupuncture, Ear/ or exp Trigger Points/ or exp Meridians/ or exp dry needling/ OR (acupuncture* OR acupuncture therapy OR acupoint* OR electroacupuncture* OR electric acupuncture* OR auriculotherapy OR auricular acupuncture* OR elongated needl* OR scalp acupuncture* OR hand acupuncture* OR manual acupuncture* OR body acupuncture* OR warm* acupuncture* OR warm* needl* OR fire needl* OR trigger point* OR meridian* OR needl* OR abdominal acupuncture* OR float* needl* OR embedded acupuncture* OR intradermal acupuncture* OR needl* OR electro-acupuncture* OR micro-puncture* OR needl* embedding).ab,kw,ti.
	2	exp General Surgery/ or exp Postoperative Complications/ or exp "Postoperative Nausea and Vomiting"/ or exp Postoperative Period/ or (surger* or operation* or postoperati* or postsurgical

For peer review only

		or postoperati* or excision* or resecti*).ab,kw,ti.
	3	exp Gastroparesis/ or exp Gastrointestinal Motility/ or exp Gastric Emptying/ or (gastroparesis or stasis, gastric or delay* gastric emptying or disturbance of gastric emptying or weakness of gastric emptying or weakness of stomach or stomach palsy or gastr* motility disorder or gastric stasis or gastroparesis syndrome or impaired gastric accommodation or stomach paresis).ab,kw,ti.
	4	((randomised controlled trial or controlled clinical trial or pragmatic clinical trial).pt. or (random* or placebo or trial or groups).ti,ab. or drug therapy.fs.) not (animals not (humans and animals)).sh.
	5	#1 AND #2 AND #3 AND #4
Web of Science	1	TS=(acupuncture* OR acupuncture therapy OR acupoint* OR electroacupuncture* OR electric acupuncture* OR auriculotherapy OR auricular acupuncture* OR elongated needl* OR scalp acupuncture* OR hand acupuncture* OR manual acupuncture* OR body acupuncture* OR warm* acupuncture* OR warm* needl* OR fire needl* OR trigger point* OR meridian* OR needl* OR abdominal acupuncture* OR float* needl* OR embedded acupuncture* OR intradermal acupuncture* OR electro-acupuncture* OR micro-puncture* OR needl* embedding OR dry needl*)
	2	TS=(surger* OR operation* OR postoperati* OR postsurgical OR excision* OR resecti*)
	3	TS=(gastroparesis OR gastroparesis OR gastric stasis OR gastric stases OR stasis, gastric OR stases, gastric OR gastr* paralysis OR delay* gastric emptying OR disturbance of gastric emptying OR weakness of gastric emptying OR weakness of stomach OR stomach palsy OR gastr* motility disorder OR gastroparesis syndrome OR impaired gastric accommodation OR stomach paresis)
	4	ALL=((Randomized controlled trial) or ((controlled) "near/7" (study or design or trial)) or (random*) or (intermethod comparison) or (placebo) or (compar*) or ((singl* or doubl* or trebl* or tripl*) "near" (blind* or mask*)) or (cross over)) or TS=(trial)
	5	#1 AND #2 AND #3 AND #4
EBSCO	1	(SU (acupuncture* OR “acupuncture therapy” OR acupoint* OR electroacupuncture* OR “electric acupuncture*” OR auriculotherapy OR “auricular acupuncture*” OR “elongated needl*” OR “scalp acupuncture*” OR “hand acupuncture*” OR “manual acupuncture*” OR “body acupuncture*” OR “warm* acupuncture*” OR “warm* needl*” OR “fire needl*” OR “trigger point*” OR meridian* OR needl* OR “abdominal acupuncture*” OR “float* needl*” OR “embedded acupuncture*” OR “intradermal acupuncture*” OR electro-acupuncture* OR micro-puncture* OR “needl* embedding” OR “dry needl*”)) OR (TI (acupuncture* OR “acupuncture therapy” OR acupoint* OR electroacupuncture* OR “electric acupuncture*” OR auriculotherapy OR “auricular acupuncture*” OR “elongated needl*” OR “scalp acupuncture*” OR “hand acupuncture*” OR “manual acupuncture*” OR “body acupuncture*” OR “warm* acupuncture*” OR “warm* needl*” OR “fire needl*” OR “trigger point*” OR meridian* OR needl* OR “abdominal acupuncture*” OR “float* needl*” OR “embedded acupuncture*” OR “intradermal acupuncture*” OR electro-acupuncture* OR micro-puncture* OR “needl* embedding” OR “dry needl*”)) OR (AB (acupuncture* OR “acupuncture therapy” OR acupoint* OR electroacupuncture* OR “electric acupuncture*” OR auriculotherapy OR “auricular acupuncture*” OR “elongated needl*” OR “scalp acupuncture*” OR “hand acupuncture*” OR “manual acupuncture*” OR “body acupuncture*” OR “warm* acupuncture*” OR “warm* needl*” OR “fire needl*” OR “trigger point*” OR meridian* OR needl* OR “abdominal acupuncture*” OR “float* needl*” OR “embedded acupuncture*” OR “intradermal acupuncture*” OR electro-acupuncture* OR micro-puncture* OR “needl* embedding” OR “dry needl*”))

For peer review only

	2	(SU (surger* OR operation* OR postoperati* OR postsurgical OR excision* OR resecti*) OR (TI (surger* OR operation* OR postoperati* OR postsurgical OR excision* OR resecti*)) OR (AB (surger* OR operation* OR postoperati* OR postsurgical OR excision* OR resecti*))
	3	(SU (gastroparesis OR gastropareses OR gastric stasis OR gastric stases OR “gastr* paralysis” OR “delay* gastric emptying” OR “disturbance of gastric emptying” OR “weakness of gastric emptying” OR “weakness of stomach” OR “stomach palsy” OR “gastr* motility disorder” OR “gastroparesis syndrome” OR “impaired gastric accommodation” OR “stomach paresis”)) OR (TI (gastroparesis OR gastropareses OR gastric stasis OR gastric stases OR “gastr* paralysis” OR “delay* gastric emptying” OR “disturbance of gastric emptying” OR “weakness of gastric emptying” OR “weakness of stomach” OR “stomach palsy” OR “gastr* motility disorder” OR “gastroparesis syndrome” OR “impaired gastric accommodation” OR “stomach paresis”)) OR (AB (gastroparesis OR gastropareses OR gastric stasis OR gastric stases OR “gastr* paralysis” OR “delay* gastric emptying” OR “disturbance of gastric emptying” OR “weakness of gastric emptying” OR “weakness of stomach” OR “stomach palsy” OR “gastr* motility disorder” OR “gastroparesis syndrome” OR “impaired gastric accommodation” OR “stomach paresis”))
	4	(TX “Randomized controlled trial” OR “controlled N7 “study OR design or trial”” OR “random*” OR “intermethod comparison” OR “placebo” OR “compar*” OR ““singl* OR doubl* OR trebl* OR tripl*” N “blind* OR mask*”” OR “cross over”) OR TI trial OR AB trial
	5	#1 AND #2 AND #3 AND #4
Scopus	1	TITLE-ABS-KEY (acupuncture* OR "acupuncture therapy" OR acupoint* OR electroacupuncture* OR "electric acupuncture*" OR musicotherapy OR "auricular acupuncture*" OR "elongated needl*" OR "scalp acupuncture*" OR "hand acupuncture*" OR "manual acupuncture*" OR "body acupuncture*" OR "warm* acupuncture*" OR "warm* needl*" OR "fire needl*" OR "trigger point*" OR meridian* OR needl* OR "abdominal acupuncture*" OR "float* needl*" OR "embedded acupuncture*" OR "intradermal acupuncture*" OR electro-acupuncture* OR micro-puncture* OR "needl* embedding" OR "dry needl*")
	2	TITLE-ABS-KEY (surger* OR operation* OR postoperati* OR postsurgical OR excision* OR resecti*)
	3	TITLE-ABS-KEY (gastroparesis OR gastroparesis OR gastric AND stasis OR gastric AND stases OR "gastr* paralysis" OR "delay* gastric emptying" OR "disturbance off gastric emptying" OR "weakness off gastric emptying" OR "weakness off stomach" OR "stomach palsy" OR "gastr* motility disorder" OR "gastroparesis syndrome" OR "impaired gastric accommodation" OR "stomach paresis")
	4	ALL ("Randomized controlled trial" OR "controlled W/7 " study OR design OR trial "" OR "random*" OR "intermeshed comparison" OR "placebo" OR "compar*" OR "" singl* OR doubl* OR trebl* OR tripl* " W " blind* OR mask* "" OR "cross over") OR TITLE-ABS-KEY (trial)
	5	#1 AND #2 AND #3 AND #4
OpenGrey	1	acupuncture* OR “acupuncture therapy” OR acupoint* OR electroacupuncture* OR “electric acupuncture*” OR auriculotherapy OR “auricular acupuncture*” OR “elongated needl*” OR “scalp acupuncture*” OR “hand acupuncture*” OR “manual acupuncture*” OR “body acupuncture*” OR “warm* acupuncture*” OR “warm* needl*” OR “fire needl*” OR “trigger point*” OR meridian* OR needl* OR “abdominal acupuncture*” OR “float* needl*” OR “embedded acupuncture*” OR “intradermal acupuncture*” OR electro-acupuncture* OR micro-puncture* OR “needl* embedding” OR “dry needl*”
	2	gastroparesis OR gastropareses OR gastric stasis OR gastric stases OR “gastr* paralysis” OR “delay* gastric emptying” OR “disturbance of gastric emptying” OR “weakness of gastric

		emptying" OR "weakness of stomach" OR "stomach palsy" OR"gastr* motility disorder" OR "gastroparesis syndrome" OR "impaired gastric accommodation" OR "stomach paresis"
	3	#1 AND #2
CNKI	1	术后 + 手术 + 切除
	2	胃瘫综合征 + 胃瘫 + 胃轻瘫 + 胃排空延迟 + 胃排空障碍 + 胃排空无力 + 胃无力 + 胃麻痹 + 胃动力障碍
	3	针灸 + 针刺 + 针法 + 刺法 + 穴位 + 温针灸 + 温针 + 电针 + 腹针 + 浮针 + 揿针 + 皮内针 + 埋针 + 火针 + 毫针 + 热针 + 耳针 + 头皮针 + 头针
	4	#1 AND #2 AND #3
Wanfang Database	1	术后 or 手术 or 切除
	2	胃瘫综合征 or 胃瘫 or 胃轻瘫 or 胃排空延迟 or 胃排空障碍 or 胃排空无力 or 胃无力 or 胃麻痹 or 胃动力障碍
	3	针灸 or 针刺 or 针法 or 刺法 or 穴位 or 温针灸 or 温针 or 电针 or 腹针 or 浮针 or 揿针 or 皮内针 or 埋针 or 火针 or 毫针 or 热针 or 耳针 or 头皮针 or 头针
	4	#1 AND #2 AND #3
VIP	1	术后 or 手术 or 切除
	2	胃瘫综合征 or 胃瘫 or 胃轻瘫 or 胃排空延迟 or 胃排空障碍 or 胃排空无力 or 胃无力 or 胃麻痹 or 胃动力障碍
	3	针灸 or 针刺 or 针法 or 刺法 or 穴位 or 温针灸 or 温针 or 电针 or 腹针 or 浮针 or 揿针 or 皮内针 or 埋针 or 火针 or 毫针 or 热针 or 耳针 or 头皮针 or 头针
	4	#1 AND #2 AND #3
CBM	1	术后 or 手术 or 切除
	2	胃瘫综合征 or 胃瘫 or 胃轻瘫 or 胃排空延迟 or 胃排空障碍 or 胃排空无力 or 胃无力 or 胃麻痹 or 胃动力障碍
	3	主题词："针刺 + 针刺疗法"[不加权:扩展] + 自由词 19 个
	4	#1 AND #2 AND #3
ICTRP	1	acupuncture* OR (acupuncture therapy) OR acupoint* OR electroacupuncture* OR (electric acupuncture*) OR auriculotherapy OR (auricular acupuncture*) OR (elongated needl*) OR (scalp acupuncture*) OR (hand acupuncture*) OR (manual acupuncture*) OR (body acupuncture*) OR (warm* acupuncture*) OR (warm* needl*) OR (fire needl*) OR (trigger point*) OR meridian* OR needl* OR (abdominal acupuncture*) OR (float* needl*) OR (embedded acupuncture*) OR (intradermal acupuncture*) OR electro-acupuncture* OR micro-puncture* OR (needl* embedding) OR (dry needl*)
	2	gastroparesis OR gastropareses OR gastric stasis OR gastric stases OR (gastr* paralysis) OR (delay* gastric emptying) OR (disturbance of gastric emptying) OR (weakness of gastric emptying) OR (weakness of stomach) OR (stomach palsy) OR (gastr* motility disorder) OR (gastroparesis syndrome) OR (impaired gastric accommodation) OR (stomach paresis)
	3	#1 AND #2
ClinicalTrials.gov	1	acupuncture* OR "acupuncture therapy" OR acupoint* OR electroacupuncture* OR "electric acupuncture*" OR auriculotherapy OR "auricular acupuncture*" OR "elongated needl*" OR "scalp acupuncture*" OR "hand acupuncture*" OR "manual acupuncture*"
	2	gastroparesis OR gastropareses OR gastric stasis OR gastric stases OR "gastr* paralysis" OR "delay* gastric emptying" OR "disturbance of gastric emptying" OR "weakness of gastric emptying" OR "weakness of stomach" OR "stomach palsy"
	3	#1 AND #2
ChiCTR	1	胃瘫综合征 or 胃瘫 or 胃轻瘫 or 胃排空延迟 or 胃排空障碍 or 胃排空无力 or 胃无力 or 胃麻痹 or 胃动力障碍

For peer review only

Supplemental Table 3 Basic characteristics of the included studies

Study ID	Study period	Age (years)	Sample size (M/F)	Surgical site (Ab/Th)	Intervention			Outcomes	Multicenter trial	Funding
					Control (mg)	Experiment	Course (days)			
Chen 2013	2009-2011	E:48-76<br>C:45-78	E:14/6<br>C:12/8	E:20/0<br>C:20/0	Mosa (15)	C; FN 30min qd	14	1)9)	No	Provincial
Chen 2016	2010-2015	E:60<br>C:58	E:15/10<br>C:14/11	E:22/3<br>C:22/3	Prokinetic	C; FN, EA, WNM 30min bid	14	1)2)3)4)9)	No	NA
Dong 2017	2010-2017	E:59.12<br>C:60.03	E:13/18<br>C:15/16	E:31/0<br>C:31/0	Meto (20)	C; FN, EA, WNM 30min bid	N/A	1)2)3)4)6)7)8)11)12)13)	No	Provincial
Huang 2020	2018-2019	E:56<br>C:56	E:36<br>C:35	E:36/0<br>C:35/0	Meto (20)	C; FN 1min bid	7	1)3)4)6)	No	NA
Li 2005	N/A	E:54<br>C:50	E:37<br>C:30	E:37/0<br>C:30/0	Cisa (30)	C; FN, EA 20min qd	7	5)	No	NA

For peer review only

Lin 2017	2016	E:60.50<br>C:61.00	E:27<br>C:29	E:30/0<br>C:28/2	Meto (10)	C; FN 30min qd	Until cured	3)5)6)9)	No	NA
Liu 2017	2008-2012	E:37.2<br>C:38.1	E:15/11<br>C:13/7	E:26/0<br>C:20/0	Cisa (30), EM (500)	C; FN 25min qd	20	1)2)	No	NA
Ma 2014	2007-2012	35-80	E:29<br>C:29	E:29/0<br>C:29/0	Meto (20), Mosa (30-60), EM, Seda	C; FN 30min qd	N/A	7)	No	NA
Meng 2012	2006-2011	E:56<br>C:55	E:14/10<br>C:8/4	E:24/0<br>C:12/0	Meto, Cisa, EM	C; FN, EA 20min qd	14	1)2)5)	Yes	NA
Sun 2010	2007-	E:61.3<br>C:59.9	E:11/7<br>C:12/6	E:0/18<br>C:0/18	Mosa (15)	C; FN 20min qd	N/A	1)	No	NA
Wang 2013	2010-2012	44.37	E:11<br>C:11	E:11/0<br>C:11/0	Prokinetic	C; FN, EA, WNM 30min qd	21	1)9)	No	NA

For peer review only

Yang 2010	2007-2009	E:60.66<br>C:63.7	E:11/8<br>C:11/7	E:19/0<br>C:18/0	Meto (40)	C; FN, EA<br>20min qd-bid	28	1)2)7)	No	NA
Zhang 2021	2019-2020	E:46.78<br>C:46.25	E:0/33<br>C:0/32	E:34/0<br>C:34/0	Mosa (15)	C; FN qd	7	1)2)3)4)6)7)8)	No	Provincial
Zhang 2022	2018-2020	58.58	E:14/12<br>C:13/9	E:26/0<br>C:22/0	Mosa (15), Dom (30)	C; FN 30min qd	14	1)2)5)6)9)	No	NA
Zhao 2021	2017-2019	E:62.6<br>C:62.5	E:15/18<br>C:17/15	E:28/5<br>C:25/7	Cisa (30)	C; FN, WNM 30min qd	14	1)2)5)6)10)	No	Provincial

E, experiment group; C, control group; M, male; F, female; Ab, abdominal surgery; Th, thoracic surgery; Cisa: Cisapride; EM, Erythromycin; Meto, Metoclopramide; Mosa, Mosapride; Seda, Sedative; Dom, Domperidone; FN, filiform needle; EA, electro-acupuncture; WNM, warming needle moxibustion; 1), total effective rate; 2), recovery rate; 3), motilin; 4), gastrin; 5), gastric juice volume; 6), days of gastrointestinal motility recovery; 7), days of gastrointestinal decompression; 8), days of resume diet for the first time; 9), clinical symptom score; 10), adverse events; 11), albumin; 12), hemoglobin; 13), transferrin.

For peer review only

Supplemental Table 4 Evidence quality of each study (GRADE)

Outcome	Quality assessment						Relative effect (95% CI)	Absolute effect	Quality
	No. of studies	Design	Risk of bias	Inconsistency	Indirectness	Imprecision	Other considerations			
Total effective rate	12	randomised trials	serious	no serious inconsistency	no serious indirectness	no serious imprecision	none	RR 1.23 (1.14 to 1.33)	-	⊕⊕⊕O MODERATE
Recovery rate	8	randomised trials	serious	no serious inconsistency	no serious indirectness	no serious imprecision	none	RR 1.55 (1.29 to 1.86)	-	⊕⊕⊕O MODERATE
MTL	5	randomised trials	serious	serious	no serious indirectness	no serious imprecision	none	-	MD 60.37 higher (37.26 to 83.47 higher)	⊕⊕OO LOW
GAS	4	randomised trials	serious	no serious inconsistency	no serious indirectness	no serious imprecision	none	-	MD 47.9 higher (32.15 to 63.64 higher)	⊕⊕⊕O MODERATE
Gastric juice volume	5	randomised trials	very serious	no serious inconsistency	no serious indirectness	no serious imprecision	none	-	MD 202.95 lower (216.96 to 170.2 lower)	⊕⊕OO LOW
Days of gastrointestinal motility recovery	6	randomised trials	very serious	serious	no serious indirectness	no serious indirectness	none	-	MD 4.87 lower (7 to 1.94 lower)	⊕OOO VERY LOW

For peer review only

Days of gastrointestinal decompression	4	randomised trials	serious	serious	no serious indirectness	no serious indirectness	none	-	MD 3.47 lower (5.74 to 1.21 lower)	⊕⊕OO LOW
Days of resume diet for the first time	2	randomised trials	no serious indirectness	serious	no serious indirectness	serious	none	-	MD 2.68 lower (4.23 to 1.12 lower)	⊕⊕OO LOW
Clinical symptom score	5	randomised trials	very serious	serious	no serious indirectness	serious	none	-	MD 1.07 lower (2.24 lower to 0.11 higher)	⊕OOO VERY LOW

For peer review only

Supplemental Table 5 Sensitivity analysis

	Total effective rate	Recovery rate	Motilin	Gastrin	Gastric juice volume	Days of gastrointestinal motility recovery	Days of gastrointestinal decompression	Days of resume diet for the first time	Clinical symptom score
Random	1.18 (95% CI 1.09, 1.27)	1.46 (95% CI 1.22, 1.74)	60.37 (95% CI 37.26, 83.47)	47.90 (95% CI 32.15, 63.64)	-193.58 (95% CI -216.96, -170.20)	-4.87 (95% CI -7.80, -1.94)	-3.47 (95% CI -5.74, -1.21)	-2.68 (95% CI -4.23, -1.12)	-1.07 (95% CI -2.24, 0.11)
Fixed	1.23 (95% CI 1.14, 1.33)	1.55 (95% CI 1.29, 1.86)	47.55 (95% CI 40.46, 54.64)	41.04 (95% CI 34.56, 47.51)	-193.58 (95% CI -216.96, -170.20)	-5.41 (95% CI -5.88, -4.94)	-2.68 (95% CI -3.13, -2.23)	-2.87 (95% CI -3.56, -2.18)	-0.65 (95% CI -0.71, -0.58)

For peer review only

Supplemental Table 6 Egger's test

	Std_Eff	Coef.	Std. Err.	t	P>|t|	[95% Conf. Interval]	
total effective rate	slope	0.8623299	0.978937	0.88	0.399	-1.318878	3.043537
	bias	0.6521827	0.886963	0.74	0.479	-1.324094	2.628459

For peer review only

BMJ Open

Invasive acupuncture for gastroparesis after thoracic or abdominal surgery: a systematic review and meta-analysis

Journal:	*BMJ Open*
Manuscript ID	bmjopen-2022-068559.R1
Article Type:	Original research
Date Submitted by the Author:	20-Mar-2023
Complete List of Authors:	Fan, Zhenjia; Beijing University of Chinese Medicine, Third Affiliated Hospital<br>Qiu, Yuqin; The First Affiliated Hospital of Chongqing Medical University<br>Qi, Xuewei; Beijing University of Chinese Medicine, Third Affiliated Hospital<br>Xu, Jingnan; Beijing University of Chinese Medicine, Third Affiliated Hospital<br>Wan, Yuxiang; Beijing University of Chinese Medicine, Third Affiliated Hospital<br>Hao, Yingxu; China-Japan Friendship Hospital, Oncology Department of Integrated Traditional Chinese and Western Medicine<br>Niu, Wenquan; Capital Institute of Pediatrics, Center for Evidence-Based Medicine<br>Huang, Jinchang; Beijing University of Chinese Medicine, Third Affiliated Hospital
**Primary Subject Heading**:	Complementary medicine
Secondary Subject Heading:	Evidence based practice, Surgery
Keywords:	COMPLEMENTARY MEDICINE, Motility disorders < GASTROENTEROLOGY, STATISTICS & RESEARCH METHODS, SURGERY

SCHOLARONE™
Manuscripts

I, the Submitting Author has the right to grant and does grant on behalf of all authors of the Work (as defined in the below author licence), an exclusive licence and/or a non-exclusive licence for contributions from authors who are: i) UK Crown employees; ii) where BMJ has agreed a CC-BY licence shall apply, and/or iii) in accordance with the terms applicable for US Federal Government officers or employees acting as part of their official duties; on a worldwide, perpetual, irrevocable, royalty-free basis to BMJ Publishing Group Ltd ("BMJ") its licensees and where the relevant Journal is co-owned by BMJ to the co-owners of the Journal, to publish the Work in this journal and any other BMJ products and to exploit all rights, as set out in our licence.

The Submitting Author accepts and understands that any supply made under these terms is made by BMJ to the Submitting Author unless you are acting as an employee on behalf of your employer or a postgraduate student of an affiliated institution which is paying any applicable article publishing charge ("APC") for Open Access articles. Where the Submitting Author wishes to make the Work available on an Open Access basis (and intends to pay the relevant APC), the terms of reuse of such Open Access shall be governed by a Creative Commons licence – details of these licences and which Creative Commons licence will apply to this Work are set out in our licence referred to above.

Other than as permitted in any relevant BMJ Author's Self Archiving Policies, I confirm this Work has not been accepted for publication elsewhere, is not being considered for publication elsewhere and does not duplicate material already published. I confirm all authors consent to publication of this Work and authorise the granting of this licence.

For peer review only

Invasive acupuncture for gastroparesis after thoracic or abdominal surgery: a systematic review and meta-analysis

Zhenjia Fan[1], Yuqin Qiu[2], Xuewei Qi[1], Jingnan Xu[1], Yuxiang Wan[1], Yingxu Hao[3], Wenquan Niu[4*], Jinchang Huang[1*]

*** Correspondence:**
Jinchang Huang, Postal Address: 51 Xiaoguan Street, Andingmenwai, Chaoyang District, Beijing, China; E-mail: zryhhuang@163.com; Telephone and Fax Number: 86-10-52075236

Wenquan Niu, Postal Address: 2 Yinghuayuan East Street, Chaoyang District, Beijing, China; E-mail: niuwenquan_shcn@163.com, Telephone and Fax Number: 86-10-84206414

Author affiliations:

[1]Third Affiliated Hospital, Beijing University of Chinese Medicine, Beijing, China

[2]First Affiliated Hospital, Chongqing Medical University, Chongqing, China

[3]Oncology Department of Integrated Traditional Chinese and Western Medicine, China-Japan Friendship Hospital, Beijing, China

[4]Center for Evidence-Based Medicine, Capital Institute of Pediatrics, Beijing, China

Keywords: Acupuncture Therapy, Gastroparesis, Postoperative Complications, Complementary Therapies

Number of References: 79

Abstract Count: 390 words.

Word count: 5655 words.

For peer review only - http://bmjopen.bmj.com/site/about/guidelines.xhtml

ABSTRACT

Objectives This meta-analysis aimed to systematically evaluate the efficacy of acupuncture in treating postsurgical gastroparesis syndrome (PGS) after thoracic or abdominal surgery.

Design Systematic review and meta-analysis.

Data sources Twelve databases [PubMed, Embase, Cochrane Library Cochrane Central Register of Controlled Trials (CENTRAL), Medline(Ovid)(from 1946), Web of Science, EBSCO, Scopus, Open Grey, China National Knowledge Infrastructure (CNKI), Wanfang Database, Chinese Scientific Journals Database (VIP), and China Biology Medicine disc (CBM)] and three registration websites [World Health Organization International Clinical Trials Registry Platform (ICTRP), ClinicalTrials.gov, and Chinese Clinical Trial Registry (ChiCTR)] were searched from the inception to September 2022, and citations of the included literature were screened.

Eligibility criteria All randomized controlled trials addressing invasive acupuncture for PGS.

Data extraction and synthesis Key information on the included studies was extracted by two reviewers independently. Risk ratio (RR) with 95% CI was used for categorical data, and mean difference (MD) with 95% CI for continuous data. The quality of evidence was assessed using Grading of Recommendations Assessment, Development and Evaluation (GRADE). Outcomes were conducted with trial sequential analysis (TSA).

Results Fifteen studies with 759 patients met the inclusion criteria. Subgroup analyses revealed that compared with the drug group, the drug and acupuncture group had a greater positive effect on the total effective rate (TER) (9 trials, n = 427; RR = 1.20; 95% CI 1.08 to 1.32; P-heterogeneity=0.20, I^2 =28 %, P = 0.0004) and the recovery rate (RCR) (6 trials, n = 294; RR = 1.61; 95% CI 1.30 to 1.98; P-heterogeneity=0.29, I^2 =19 %, P < 0.0001) of PGS after abdominal surgery. However, acupuncture showed no significant advantages in terms of the TER after thoracic surgery (1 trial, P = 0.13) or thoracic/abdominal surgery related PGS (2 trials, n = 115; RR = 1.18; 95% CI 0.89 to 1.57; P-heterogeneity=0.08, I^2 =67 %, P = 0.24) and the RCR after thoracic/abdominal surgery (2 trials, n = 115; RR = 1.40; 95% CI 0.97 to 2.01; P-heterogeneity=0.96, I^2 =0 %, P = 0.07). The quality of evidence for TER and RCR was moderate-certainty. Only one study reported an acupuncture-related adverse event, in the form of mild local subcutaneous hemorrhage and pain that recovered spontaneously. TSA indicated that outcomes reached a necessary effect size except for clinical symptom score.

Conclusion Based on subgroup analysis, compared with the drug treatment, acupuncture combined drug have significant advantages in the treatment of PGS associated with abdominal surgery, but not with thoracic surgery.

PROSPERO registration number CRD42022299189

STRENGTHS AND LIMITATIONS OF THIS STUDY

1. This systematic review and meta-analysis was performed according to the Preferred Reporting Items for Systematic Reviews and Meta-Analyses (PRISMA) statement to ensure the reproducibility of the study.

For peer review only

2. A comprehensive search strategy was used to search through a large number of databases and registration websites, and gray literatures were searched in the study.

3. The quality of evidence for each outcome was evaluated using Grading of Recommendations Assessment, Development and Evaluation (GRADE), the robustness of conclusions was examined through trial sequential analysis (TSA), and publication bias was assessed with Egger's test using Stata 16 software.

4.Outcomes were analyzed by subgroups according to surgical site, acupuncture stimulation method, and intervention course.

5. The included outcomes were immediate post-intervention time-point data, leading to a lack of analysis of the long-term effects of the intervention in this study.

INTRODUCTION

Postsurgical gastroparesis syndrome (PGS) is a gastric motility disease defined by delayed gastric emptying after eating without pyloric stenosis or another mechanical obstruction within 3 months postoperatively, with symptoms including nausea, vomiting, early satiety, postprandial fullness, and bloating [1-3]. PGS is the second most common type of gastroparesis after diabetes gastroparesis, and it often occurs after abdominal surgery [2], with an incidence rate of 0.4%-5.0% after gastric surgery [4, 5]. In addition, PGS has been reported to occur after thoracic surgery, such as esophageal surgery [6, 7], lung surgery [8, 9], and heart transplantation [10, 11], which can negatively affect patients' postoperative quality of life.

Treatment of PGS consists of dietary modification, gastrointestinal decompression (GD), fluid and electrolyte acid-base balance, nutritional support, psychological therapies, and prokinetic and antiemetic medication, but with limited benefit [1]. Additional therapeutic options such as pyloric botulinum toxin injections, gastric electrical stimulation (GES), peroral pyloromyotomy (POP), and gastric peroral endoscopic myotomy (G-POEM) are considered for PGS when medication fails, but all these approaches are invasive and have limited efficacy [12, 13]. The gastrointestinal total symptom score in patients treated with GES was reduced by 48% in Europe and 38% in the United States [14]. The effect of modern medical treatment on PGS is unsatisfying. Acupuncture is a traditional medical treatment that has been applied in many therapeutic areas and is getting more attention [15]. Acupuncture, or a combination of acupuncture and drugs, has been reported to have some advantages in the treatment of PGS in recent clinical trials [16, 17].

Invasive acupuncture, which involves piercing one or more needles into specific acupoints on the body surface, is a traditional Chinese medicine treatment method that is distinct from other local stimulation therapies such as transcutaneous electrical nerve stimulation [18]. Several studies have documented the mechanism of acupuncture for PGS as follows: (i)Regulating the secretion of gastrointestinal hormones. In an animal study, Yu et al. [19] found that acupuncturing at Zhongwan (CV12) and Zusanli (ST36) promoted the levels of motilin (MTL), gastrin (GAS), ghrelin, and growth factor 1 in serum of rats to treat gastroparesis. In a clinical trial, Xing et al. [20] found that electro-acupuncture improved gastrointestinal function and upregulated serum MTL and GAS levels in patients with acute gastrointestinal injury after 7 days of intervention. In another clinical trial, Chen et al. [21] found that acupuncture combined with domperidone more significantly upregulated the contents of MTL and GAS in plasma of patients with gastroparesis compared with domperidone alone. In a randomized controlled trial (RCT), Güçel F et al. [22] found that after several weeks of acupuncture treatment, patients' plasma ghrelin was upregulated and gastrointestinal function

For peer review only - http://bmjopen.bmj.com/site/about/guidelines.xhtml

improved. (ii)Stimulating the vagus nerve. The pathogenesis of PGS may be related to vagus nerve injury or compression and impaired gastric muscle regulation [1]. In animal studies, Yang et al. [23] and Noguchi E [24] found that electro-acupuncturing at ST36 on the hind limb stimulated the vagus nerve, which attenuated motility dysfunction in intestinal muscularis and modulated gastrointestinal motility. In a clinical trial, Liu et al. [25] found that the improvements in volunteers' fullness, satiety, bloating, nausea, vomiting and gastric slow waves were closely related to the stimulation of the vagus nerve by acupuncture. (iii)Reducing damage to interstitial cells of Cajal (ICCs). ICCs are considered pacemaker cells in the gastrointestinal tract that transmit electrical signals for gastric emptying [1]. In an animal study, Tian et al. [26] found that electro-acupuncturing at ST36 protected ICCs by regulating macrophages to improve gastric motility. In a clinical trial, Song et al. [27] found that the improvement of gastroparesis by acupuncture could be related to reducing the damage to ICCs.

Several previous clinical trials have observed therapeutic effects of acupuncture on PGS, such as improving symptoms of nausea, vomiting, early satiety, and bloating; reducing the time to resumption of diet for the first time (RDT); promoting gastric emptying; downgrading gastric juice volume (GJV); and upgrading MTL and GAS in serum [17, 28, 29]. Further clinical trials are in progress [30, 31].

The lack of convincing evidence for the efficacy of acupuncture in the treatment of PGS due to the small sample size and different trial protocols has hampered its clinical application. This study systematically reviewed RCTs of acupuncture for PGS by meta-analysis to provide evidence for the clinical application of acupuncture.

METHODS

Study registration

The protocol for this systematic review and meta-analysis was registered in PROSPERO (CRD42022299189), and the study was performed according to the Preferred Reporting Items for Systematic Reviews and Meta-Analyses (PRISMA) statement as shown in online supplemental table 1 [32].

Data sources and search strategy for study registration

We searched, with no language restrictions, 12 databases [PubMed, Embase, Cochrane Library Cochrane Central Register of Controlled Trials (CENTRAL), Medline(Ovid)(from 1946), Web of Science, EBSCO, Scopus, Open Grey, China National Knowledge Infrastructure (CNKI), Wanfang Database, Chinese Scientific Journals Database (VIP), and China Biology Medicine disc (CBM)] and 3 registration websites [World Health Organization International Clinical Trials Registry Platform(ICTRP), ClinicalTrials.gov, and Chinese Clinical Trial Registry (ChiCTR)] from their creation to September 2022 for RCTs. Search strategies are detailed in online supplemental table 2. We adapted search strategy appropriately for each database. In addition, we screened citations of the included articles manually to avoid search omissions.

Study selection

We screened titles and abstracts of articles using Endnote X7 software, and the full-texts were screened according to the selection criteria of the study.

For peer review only - http://bmjopen.bmj.com/site/about/guidelines.xhtml

Inclusion criteria: (i)Participants of the trails were diagnosed with PGS [4]. There are no standardized criteria for PGS diagnosis, so we adopted the diagnostic criteria formulated by Professor Qin's team at Zhongshan Hospital, Fudan University, Shanghai, China, after researching the relevant documents [1, 3, 33-35]. (ii)The control group of the trial was treated with prokinetic drugs in addition to the routine treatment. (iii)The experimental group of the study was treated with acupuncture on the basis of the control group. (iv)Acupuncture was defined as the penetration of a filiform needle invasively into the skin with or without electrical or moxibustion stimulation. (v)The trial was an RCT with no blinding restrictions.

Exclusion criteria: (i)Duplicated publications. (ii)The experimental group of the study used other Chinese medical treatments in addition to acupuncture, including but not limited to oral herbs, non-invasive external therapies such as moxibustion without acupuncture, acupressure, acupoint application, and herbal enema. (iii)Missing or uncleared data and an inability to contact the author for further details. (iv)Animal or cell experiments. (v)Reviews.

Two reviewers screened and selected articles independently. Disagreements between the reviewers were resolved by discussion with a third reviewer.

Outcome assessment

The primary outcomes of the study were the total effective rate (TER) and the recovery rate (RCR). In the trials included in this meta-analysis, the evaluation criteria for TER and RCR of PGS were centered on these proposed by the China Association of Chinese Medicine (CACM), which are the combination of objective indicators (gastric juice volume, gastrointestinal radiography) and subjective indicators (whether nausea, vomiting, and bloating disappear) [36]. TER refers to the proportion of recovered and effective people in all participants. RCR refers to the proportion of recovered people in all participants. The secondary outcomes were serum MTL, serum GAS, GJV, days to gastrointestinal motility recovery (GMR), days to GD, days to RDT, and clinical symptom score (CSS).

Data extraction and management

We extracted and sorted relevant data of the included studies using Microsoft Excel 2019 and displayed them in a table. The data included the first author, year of publication, trial duration, number and age of patients in each group, surgical site, treatment methods and courses of intervention and control groups, acupoint, and outcomes. Dichotomous data were the number of events and the number of participants per group; continuous data were presented as mean, standard deviation (SD), and number of participants per group.

Data extraction and management were completed by two reviewers independently and a third reviewer joined the discussion and made a decision when disagreements arose between reviewers.

Assessment of risk of bias and quality of evidence

Two reviewers independently evaluated the risk bias of the included studies based on the Cochrane Handbook which contains seven items: 1) random sequence generation (selection bias); 2) allocation concealment (selection bias); 3) blinding of participants and personnel (performance bias); 4) Blinding of outcome assessment (detection bias); 5) incomplete outcome data (attrition bias); 6) selective reporting (reporting bias); and 7) other biases [37]. Three levels (high risk, low risk, and unclear risk) were used during the evaluation. Any disagreements during the assessment process were

For peer review only - http://bmjopen.bmj.com/site/about/guidelines.xhtml

discussed and evaluated by a third senior professional. Review Manager 5.3 software was used for building the summary and graph of the risk of bias. Furthermore, this study assessed the quality of evidence for each outcome and expressed it as high, medium, low, or very low using GRADE pro 3.6 [38, 39].

Statistical analysis

The systematic review and meta-analysis were completed with Review Manager 5.3 software. During data analysis, the risk ratio (RR) with 95% confidence interval (CI) was used for categorical data, while the mean difference (MD) with 95% confidence interval (CI) was used for continuous data. The results were considered statistically significant when $p < 0.05$. Heterogeneity was assessed by the Cochran's Chi-squared test and quantified by the I^2 statistic test. If there was significant heterogeneity among studies ($p<0.10$ or $I^2>50\%$), the random effects model was used; otherwise, the fixed effects model was used when $p\geq0.10$ or $I^2\leq50\%$. Subgroup analyses were conducted according to surgical site, acupuncture stimulation method, and intervention course for heterogeneity analysis. Sensitivity analysis was performed to determine the stability of the results by converting the fixed effect model and random effect model, and removing single study in turn to observe its impact on the summary results. Publication bias was assessed with Egger's test by Stata 16 software and the funnel diagram plotted with Review Manager 5.3 software. Trial sequential analysis (TSA) was conducted using TSA version 0.9.5.10 Beta (Copenhagen Trial Unit) software.

Patient and public involvement statement

Neither patients nor the public were involved in the design, conduct, reporting, or dissemination plans of this research.

RESULTS

Study selection

The flowchart of the article screening process in the meta-analysis is shown in figure 1, which was derived from the PRISMA statement [32]. A total of 2549 articles were searched, 2513 of which were from databases and 36 from registers. A total of 121 articles were selected for full text screening by reading the titles and abstracts. 14 articles [40-53] were finally included after 107 articles had been excluded for reasons such as unclear diagnosis, inappropriate intervention method, study type other than RCT, or unclear data. In addition, we screened 262 articles cited in the 14 included articles, and 1 of these met the inclusion criteria [54]. As a result, a total of 15 studies were included in this meta-analysis.

Study characteristics

The basic characteristics of the included studies are presented in online supplemental table 3. The included studies were all completed in China and included 759 patients, with sample sizes ranging from 22 to 71 participants. One of the 15 studies was a multi-center trial, and the rest were single-center trials. The mean age of patients was over 50 years in 10 studies, under 50 years in 3 studies, and not mentioned in 2 studies. 3 studies included PGS after thoracic or abdominal surgery, 1 study included simple thoracic surgery, and the rest included simple abdominal surgery. The control group (Drug Group) was treated with prokinetic drugs in all of the included studies, and the experimental group (Drug and Ac Group) was treated with a filiform needle on the basis of the control group. In 8 studies, the experimental group was treated with an individual filiform needle, which was labeled

For peer review only - http://bmjopen.bmj.com/site/about/guidelines.xhtml

manual acupuncture (MAc), while in 7 studies, electrical stimulation or moxibustion stimulation was added based on a filiform needle, which was labeled stimulated manual acupuncture (SMAc). A number of studies used multiple acupoints, except for Huang 2020 who stimulated only a pair of acupoints. A total of 11 studies clearly described the treatment course, of which 8 studies had a treatment course of ≤ 14 days and 3 studies had a treatment course of >14 days. The frequency of acupuncturing was once daily in 11 studies, twice daily in 3 studies, and between once and twice per day in 1 study. For primary outcomes, 12 studies reported the TER and 8 reported the RCR. Four studies received financial support from provincial projects.

Risk of bias and study quality

All of the studies mentioned a random design, with 10 studies (66.67%) clearly reporting random sequence generation processes and only 1 study (6.67%) describing the blinding of participants. 4 studies (26.67%) reported case shedding. The summary results of the risk of bias are detailed in figure 2. According to the GRADE results, the outcomes of the TER, the RCR and GSA showed moderate-certainty evidence; the MTL, GJV, GD, and RDT showed low-certainty evidence; and the GMR and CSS showed very low-certainty evidence (online supplemental table 4).

Main outcomes

TER

12 trials reported the TER (figure 3A). There were 302 patients in the Drug and Ac Group and 276 patients in the Drug Group. Test for the overall effect indicated that the TER of the Drug and Ac Group was significantly higher than that of the Drug Group (12 trials, n = 578; RR = 1.18; 95% CI 1.09 to 1.27; *P*-heterogeneity=0.22, I^2 =23%, $P < 0.0001$). The sensitivity analysis showed that the study reported by Huang 2020 was the source of instability, which may be related to acupuncturing at only one pair of acupoints (online supplemental table 5). We downgraded evidence by one level for the lack of allocation concealment and the lack of blinding. We judged the evidence to be of moderate-certainty.

The results of the subgroup analysis of surgical sites revealed that the Drug and Ac Group had a statistically significant advantage in terms of the TER of PGS after abdominal surgery (9 trials, n = 427; RR = 1.20; 95% CI 1.08 to 1.32; *P*-heterogeneity=0.20, I^2 =28 %, P = 0.0004), but not after thoracic surgery (1 trial, P = 0.13) and thoracic/abdominal surgery (2 trials, n = 115; RR = 1.18; 95% CI 0.89 to 1.57; *P*-heterogeneity=0.08, I^2 =67 %, P = 0.24)(figure 3A). Furthermore, subgroup analysis revealed that the overall effect of the TER was not significantly related to the acupuncture course (online supplemental figure 1A) or the method of acupuncture stimulation (individual filiform needle, electrical stimulation or moxibustion stimulation) (online supplemental figure 1B).

RCR

8 studies reported the RCR (figure 3B). There were 217 patients in the Drug and Ac Group and 192 patients in the Drug Group. Testing for the overall effect indicated that the RCR of the Drug and Ac Group was significantly higher than that of the Drug Group (8 trials, n = 409; RR = 1.55; 95% CI 1.29 to 1.86; *P*-heterogeneity=0.54, I^2 = 0%, $P < 0.00001$). Sensitivity analysis showed that the conclusion was stable (online supplemental table 5). We downgraded evidence by one level for the lack of allocation concealment and the lack of blinding. We judged the studies as moderate-certainty evidence.

For peer review only - http://bmjopen.bmj.com/site/about/guidelines.xhtml

The results of the subgroup analysis of surgical sites revealed that the Drug and Ac Group had a statistically significant advantage in terms of the RCR of PGS after abdominal surgery (6 trials, n = 294; RR = 1.61; 95% CI 1.30 to 1.98; *P*-heterogeneity=0.29, I^2 =19 %, P <0.0001), but not after thoracic/abdominal surgery (2 trials, n = 115; RR = 1.40; 95% CI 0.97 to 2.01; *P*-heterogeneity=0.96, I^2 =0 %, *P* = 0.07) (figure 3B). The results of the subgroup analysis of acupuncture course revealed that the Drug and Ac Group had a statistically significant advantage in terms of the RCR of PGS when the course was ≤14 days (5 trials, n = 264; RR = 1.68; 95% CI 1.31 to 2.16; *P*-heterogeneity=0.29, I^2 =19%, P < 0.0001), but when the course was ＞14 days (2 trials, n = 83; RR = 1.29; 95% CI 0.95 to 1.74; *P*-heterogeneity=0.98, I^2 =0 %, P = 0.10)(online supplemental figure 2A). The results of the subgroup analysis of the method of acupuncture stimulation revealed that the Drug and Ac Group had a significant advantage in terms of the RCR of PGS when treated with SMAc (6 trials, n = 296; RR = 1.40; 95% CI 1.15 to 1.71; *P*-heterogeneity=0.96, I^2 =0%, P = 0.0007), but not when treated with MAc (2 trials, n =113; RR = 2.24; 95% CI 0.70 to 7.15; *P*-heterogeneity=0.03, I^2 =79%, P = 0.17) (online supplemental figure 2B).

Secondary outcomes

MTL

MTL, which has a positive effect on PGS, is a key gastrointestinal hormone that can facilitate gastrointestinal motility by raising the secretion of pepsin and acid in the stomach and causing contraction of the stomach [55]. Serum MTL levels in healthy individuals are around 499.02 pg/mL [56]. 5 studies reported the MTL levels (figure 4A). There were 152 patients in the Drug and Ac Group and 152 patients in the Drug Group. Testing for the overall effect indicated that the MTL level of the Drug and Ac Group was significantly higher than that of the Drug Group (5 trials, n = 304; MD = 68.15; 95% CI 42.89 to 93.40; *P*-heterogeneity < 0.0001, I^2 = 86%, *P* < 0.00001). The sensitivity analysis showed that the study reported by Lin 2017 was the source of instability, which may be related to the high risk of bias (online supplemental table 5). We downgraded evidence by two levels for the lack of allocation concealment, the lack of blinding, and unexplained heterogeneity. We judged the studies as low-certainty evidence.

The results of the subgroup analysis of surgical sites revealed that the Drug and Ac Group had a statistically significant advantage in terms of the MTL levels of PGS after both abdominal surgery (3 trials, n = 198; MD = 48.76; 95% CI 37.98 to 59.54; *P*-heterogeneity=0.47, I^2 = 0 %, *P* < 0.00001) and thoracic/abdominal surgery (2 trials, n = 106; MD = 101.73; 95% CI 44.73 to 158.73; *P*-heterogeneity = 0.003, I^2 = 89 %, *P* = 0.0005), but with high heterogeneity (figure 4A).

GAS

GAS is another gastrointestinal hormone that enhances gastric motility, and has a positive effect on PGS [57]. Serum MTL levels in healthy individuals are around 92.95 pg/mL [56]. 4 studies reported the GAS levels (figure 4B). There were 125 patients in the Drug and Ac Group and 123 patients in the Drug Group. Testing for the overall effect indicated that the GAS levels of the Drug and Ac Group was significantly higher than that of the Drug Group (4 trials, n = 248; MD = 40.28; 95% CI 30.86 to 49.70; *P*-heterogeneity=0.05, I^2 = 61 %, *P* < 0.00001). Sensitivity analysis showed that the conclusion was stable despite high heterogeneity (online supplemental table 5). However, the cause of heterogeneity was not found, and higher quality trials may be needed for further study. We downgraded evidence by one level for the lack of allocation concealment and the lack of blinding. We judged the studies as moderate-certainty evidence.

For peer review only - http://bmjopen.bmj.com/site/about/guidelines.xhtml

The results of the subgroup analysis of surgical sites revealed that the Drug and Ac Group had a statistically significant advantage in terms of the GAS levels of PGS after both abdominal surgery (3 trials, n = 198; MD = 37.95; 95% CI 27.63 to 48.27; P-heterogeneity=0.07, I^2 = 62 %, $P < 0.00001$) and thoracic/abdominal surgery (1 trials, $P < 0.00001$) (figure 4B).

GJV

The volume of gastric juice secreted by normal adults is 1.5~2.5 L/day [58]. Gastric juice accumulates in the stomach with gastric hypomotility. Higher levels of GJV indicate lower gastric motility. Patients' GJV levels in individuals without surgeries are around 45.2 mL/day [59]. 5 studies reported the GJV (figure 4C). There were 147 patients in the Drug and Ac Group and 125 patients in the Drug Group. Test for the overall effect indicated that the GJV of the Drug and Ac Group was significantly lower than that of the Drug Group (5 trials, n = 272; MD = -193.58; 95% CI -216.96 to -170.20; P-heterogeneity=0.85, I^2 = 0 %, $P < 0.00001$). Sensitivity analysis showed that the conclusion was stable (online supplemental table 5). We downgraded evidence by two levels for the lack of allocation concealment, the lack of blinding, and incomplete accounting of patients and outcome events. We judged the studies as low-certainty evidence.

The results of the subgroup analysis revealed that the advantage of the Drug and Ac Group in regulating GJV was not affected by the surgical site (figure 4C) or acupuncture method (online supplemental figure 3).

Days to GMR

GMR is an indicator of the effectiveness of PGS treatment, with smaller values indicating better efficacy. 6 studies reported the days to GMR (figure 5A). There were 186 patients in the Drug and Ac Group and 181 patients in the Drug Group. Testing for the overall effect indicated that there were significantly fewer days to GMR in the Drug and Ac Group than in the Drug Group (6 trials, n = 367; MD = -4.87; 95% CI -7.80 to -1.94; P-heterogeneity < 0.00001, I^2 = 97 %, $P = 0.001$). Sensitivity analysis showed that the conclusion was stable despite high heterogeneity (online supplemental table 5). We downgraded evidence by three levels for the lack of allocation concealment, the lack of blinding, incomplete accounting of patients and outcome events, and unexplained heterogeneity. We judged the studies as very low-certainty evidence.

The results of the subgroup analysis revealed that the advantage of the Drug and Ac Group in regulating GMR was not affected by surgical site (figure 5A).

Days to GD

GD is an indicator of the effectiveness of PGS treatment, with smaller values indicating better efficacy. 4 studies reported the days to GD (figure 5B). There were 112 patients in the Drug and Ac Group and 110 patients in the Drug Group. Testing for the overall effect indicated that there were significantly fewer days toGD in the Drug and Ac Group compared with the Drug Group (4 trials, n = 222; MD = -3.47; 95% CI -5.74 to -1.21; P-heterogeneity < 0.00001, I^2 = 94 %, $P = 0.003$). Sensitivity analysis showed that the study reported by Ma 2014 was the source of instability (online supplemental table 5), this might be related to the fact that some of the participants in this trial were treated with sedatives, as sedatives can affect vagal activity [60]. We downgraded evidence by two levels for the lack of allocation concealment, the lack of blinding, and unexplained heterogeneity. We judged the studies as low-certainty evidence.

For peer review only - http://bmjopen.bmj.com/site/about/guidelines.xhtml

Days to RDT

RDT is an indicator of the effectiveness of PGS treatment, with smaller values indicating better efficacy. 2 studies reported the days to RDT (figure 5C). There were 64 patients in the Drug and Ac Group and 63 patients in the Drug Group. Testing for the overall effect indicated that there were significantly fewer days to RDT in the Drug and Ac Group than in the Drug Group (2 trials, n = 127; MD = -2.68; 95% CI -4.23 to -1.12; *P*-heterogeneity= 0.03, I^2 = 78 %, *P* = 0.0007). Sensitivity analysis showed that the conclusion was stable despite high heterogeneity (online supplemental table 5). The reliability of this conclusion was low due to the lack of data. We downgraded evidence by two levels for unexplained heterogeneity and the small sample size. We judged the studies as low-certainty evidence.

CSS

CSS is an indicator of the effectiveness of PGS treatment, with smaller scores indicating better efficacy.3 studies reported the CSS, and they were scored with the same criteria (figure 5D). There were 62 patients in the Drug and Ac Group and 58 patients in the Drug Group. Testing for the overall effect indicated that the CSS of the Drug and Ac Group was lower than that of the Drug Group, but the difference was not statistically significant (3 trials, n = 120; MD = -1.31; 95% CI -3.24 to 0.61; *P*-heterogeneity < 0.00001, I^2 =100%, *P* = 0.18). We downgraded evidence by four levels for the lack of allocation concealment, the lack of blinding, and incomplete accounting of patients and outcome events, unexplained heterogeneity, and the small sample size. We judged the studies as very low-certainty evidence.

The results of the subgroup analysis of surgical site revealed that the Drug and Ac Group had no statistically significant advantage in terms of the CSS of PGS after either abdominal surgery (2 trials, n = 70; MD = -0.17; 95% CI -0.43 to 0.08; *P*-heterogeneity=0.01, I^2 = 85 %, *P* = 0.18) or thoracic/abdominal surgery (1 trial, < 0.0000195% CI) (figure 5D). 95% CI

In addition, 2 studies reported the CSS with two different criteria, and their conclusions were different (Chen 2013: n = 40; MD = -1.05; 95% CI -3.33 to 1.23; *P* =0.37; Lin 2017: n = 56; MD = -0.34; 95% CI -0.45 to 0.23; *P* < 0.00001).

Safety analysis

Two studies described the safety of the trials. Dong 2017 assessed nutritional indicators such as albumin, hemoglobin, and transferrin before and after the intervention. The results showed that all three indicators were significantly higher in the experimental and control groups at the end of the intervention compared with the pre-intervention period, and the three indicators in the experimental group were significantly higher than those in the control group, with statistically significant differences (P<0.05). Zhao 2021 recorded adverse events that included subcutaneous hemorrhage and pain associated with acupuncture in the experimental group, but they were all mild and recovered without special treatment. There were no acupuncture-related serious adverse events in the experimental and control groups.

Publication bias

The funnel plot of the TER was asymmetric (online supplemental figure 4), although Egger's test showed p = 0.399 > 0.05 (online supplemental table 6), indicating that there was risk of publication bias in the included studies.

For peer review only - http://bmjopen.bmj.com/site/about/guidelines.xhtml

387 **TSA**

388 TSA showed that the cumulative Z-curve of the TER and the RCR surpassed the trial sequential
389 monitoring boundary and the conventional test boundary. The cumulative Z-curve of the MTL, GAS,
390 GJV, GMR, GD, RDT surpassed the trial sequential monitoring boundary, the conventional test
391 boundary, and the required information size (RIS). The results suggested that these conclusions were
392 robust and no further trials are necessary to verify these conclusions. However, the cumulative Z-
393 curve of the CSS failed to surpassed the trial sequential monitoring boundary or the RIS, suggesting
394 that the conclusion was not robust and further trials are necessary to verify it (online supplemental
395 figure 5).

396 **Frequency analysis of acupoints**

397 The 15 studies involved 26 acupoints with a frequency of 88. The acupoints that were applied ≥3
398 times are listed in table 1 according to the application frequency from high to low, with the top three
399 being ST36, CV12 and PC6.

400 **Table 1** Summary of frequencies of the applicated acupoints (Top 9)

No.	Acupoint	Alphabetic Code	Frequency
1	Zusanli	ST36	15
2	Zhongwan	CV12	12
3	Neiguan	PC6	11
4	Shangjuxu	ST37	8
5	Tianshu	ST25	6
6	Sanyinjiao	SP6	6
7	Gongsun	SP4	5
8	Qihai	CV6	4
9	Yinlingquan	SP9	3

401 **DISCUSSION**

402 This study aimed to evaluate the efficacy of acupuncture in the treatment of PGS. A total of 15
403 studies containing 759 patients were included after systematic searching and screening of the
404 literature. Our meta-analysis suggested that, overall, acupuncture combined with prokinetic drugs had
405 a more positive efficacy on the TER, RCR, MTL, GAS, GJV, days to GMR, days to GD and days to

For peer review only - http://bmjopen.bmj.com/site/about/guidelines.xhtml

RDT in PGS compared with prokinetic drugs alone. Although there was a trend towards the positive efficacy of acupuncture combined with drugs on CSS, the difference was not statistically significant. The heterogeneity of the included CSS-related trials was significant, that may be because the CSS is a subjective indicator and there were possibilities of bias. At present, there are too few relevant trials to analyze the reasons of the heterogeneity. The results of the subgroup analysis of the main outcomes suggested that the advantage of the TER of acupuncture combined with prokinetic drugs for PGS was not influenced by the course of acupuncture, or the method of acupuncture stimulation; however, the RCR of acupuncture for PGS showed a statistically significant advantage only for the short course. It is noteworthy that acupuncture combined with prokinetic drugs had no obvious advantage in terms of the TER and RCR of PGS after thoracic surgery and thoracic/abdominal surgery, but significantly improved the TER and RCR of PGS after abdominal surgery. In addition, the therapeutic advantage in terms of secondary outcomes in the group treated with acupuncture combined with prokinetic drugs was not affected by acupuncture course, method, frequency, or surgical site, although there was heterogeneity. As for safety analysis, only one study reported mild adverse events associated with acupuncture, and another study reported the levels of albumin, hemoglobin, and transferrin in serum. Evidence was generally insufficient, and more research is needed to assess the safety of acupuncture in patients with PGS. The quality of evidence was deemed to be low due to the limitations such as the lack of allocation concealment, lack of blinding, incomplete accounting of patients and outcome events, unexplained heterogeneity, and the small sample size. TSA suggested that the conclusions, except for CSS, were robust and no further trials are necessary to verify these conclusions.

At present, prokinetic drugs are the most commonly used in PGS medication. Metoclopramide, a D2 receptor antagonist, is used with some 5-HT4 receptor agonists at the same time. As the only drug approved by FDA for gastroparesis, metoclopramide can effectively alleviate PGS-related nausea, vomiting and other symptoms of the digestive system [61]. 5-HT4 agonists such as cisapride are frequently and early adopted in PGS, and mosapride has also been used in gastroparesis [62-65]. Prucalopride is a recently discovered effective drug that accelerates gastric emptying without cardiac side effects and has been initially reported to relieve symptoms in patients with idiopathic gastroparesis or diabetic gastroparesis [66], but high-quality studies in PGS are lacking. Macrolide antibiotics such as erythromycin are agonists of GAS receptors, which induce GAS secretion in the proximal part of the digestive tract and accelerate gastric emptying. However, they face problems such as rapid drug resistance, and their clinical benefit is controversial [67, 68].

The results showed that acupuncture combined with prokinetic drugs for PGS was able to significantly improve the efficacy of the treatment and digestive symptoms of patients. Meanwhile, acupuncture is less traumatic compared with endoscopic and surgical treatments, such as pyloric botulinum toxin injections, GES, POP and G-POEM [69].

Among the 15 studies included in this meta-analysis, ST36 on the ST meridian, CV12 on the CV meridian, and PC6 on the PC meridian were the most frequently adopted acupoints, and all three acupoints play an important role in regulating stomach function. It has been reported that acupuncturing at ST36 can contract muscles [70], regulate gastric rhythm and improve gastric emptying delay by stimulating the parasympathetic nerves to treat gastroparesis [71]. Further study indicated that electro-acupuncturing at ST36 could modulate gastric motility and improve gastrointestinal symptoms through M2/3 and β1/2 receptor-mediated vagal and sympathetic reflex [72]. Electro-acupuncturing at CV12 can regulate gastric emptying and demonstrate beneficial effects on nausea and vomiting which may be mediated through the inhibition of 5-HT secretion in the duodenum and activity of the nucleus of the solitary tract [73, 74]. Stimulating at PC6 has an effect

For peer review only - http://bmjopen.bmj.com/site/about/guidelines.xhtml

on gastrointestinal symptoms and prevents postoperative nausea and vomiting [75-77]. Electro-acupuncturing at PC6 reduces the inhibition of efferent vagus nerve motor fibers by inhibiting GABA transmission to dorsal motor nucleus of the vagus, thereby promoting efferent vagus nerve activity and increasing gastric motility [78]. In clinical practice, ST36, CV12 and PC6 are often stimulated in combination to improve digestive symptoms, and have been proven to be effective [79].

In general, the meta-analysis of previous studies of acupuncture for PGS showed that acupuncture combined with prokinetic drugs improved the TER and RCR of PGS. However, more evidences were needed on the relationship between surgical site and the TER and the RCR, and the relationship between the RCR and the acupuncture course or the method of acupuncture stimulation. In addition, acupuncture can up-regulate the levels of MTL and GAS in serum, decrease GJV, accelerate GMR, and shorten days to GD and RDT.

Clinical trials on acupuncture for PGS are generally single-center studies based on the small sample size, so the efficacy remains unclear. This meta-analysis provided an extensive search and analysis for RCTs about PGS to comprehensively evaluate the therapeutic effect of acupuncture. We tried to provide an approach for clinicians and researchers to treat patients with PGS. In addition, we counted the acupoints involved in the included trials to provide reference to select acupoints and formulate treatment plans.

There has not been any meta-analysis of acupuncture for PGS after thoracic or abdominal surgery. We conducted subgroup analysis according to surgical sites and found differences in efficacy. Besides, we excluded literatures with other Chinese medical treatments such as oral herbs, acupressure, and herbal enema, and focused on acupuncture to make the interventions in different trials as consistent as possible. Furthermore, we assessed the quality of evidence using GRADE and evaluated the robustness of conclusions with TSA to make the methodology more rigorous.

However, this meta-analysis has some limitations. Firstly, as mentioned, 759 participants were included in this study, which is a small sample size and there is a risk of bias. Secondly, all of the included studies were completed in China, and there may be racial and geographical variability in the meta-analysis. Thirdly, the types, doses, and methods of prokinetic drugs used in the studies were inconsistent. Fourthly, in the analysis of CSS, the difference between experimental group and control group was not statistically significant and the heterogeneity was very large, more high-quality studies are needed. Fifthly, there was a risk of publication bias in this study. Sixthly, the grades of PGS were not mentioned in the included studies, which may be a key factor influencing the therapeutic effect of acupuncture on PGS. Seventhly, the thoracic surgeries included in this meta-analysis were all esophageal cancer surgeries, which led to limited data, and further in-depth studies should demonstrate the efficacy of acupuncture for PGS after other types of thoracic surgery reliably. Eighthly, only two of the included studies reported safety data, and more trials are demanded. Finally, although TSA indicated that the conclusions were robust, bias risk existed in the included studies and more high-quality studies are needed to substantiate them.

CONCLUSIONS

This meta-analysis demonstrated that compared with the use of prokinetic drugs alone, acupuncture with prokinetic drugs for PGS associated with abdominal surgery is more advantageous. It's worthy of clinical promotion because of the improved efficacy. However, the efficacy of acupuncture for PGS associated with thoracic surgery is unclear. Due to the limitations of the included studies, high

For peer review only - http://bmjopen.bmj.com/site/about/guidelines.xhtml

quality trials with rigorous design and large sample sizes are needed to further confirm the efficacy and safety of acupuncture for PGS.

Contributors Huang J proposed the original concept and Fan Z designed the study. Qiu Y and Qi X screened the abstract and full text. Qi X and Xu J extracted the data. Wan Y and Xu J evaluated the risks of bias. Fan Z and Qiu Y carried out the outcomes and wrote the manuscript. Hao Y revised the manuscript and arbitrated disagreements. Niu W guided the meta-analysis.

Funding This work was supported by Key Research Project of Beijing University of Chinese Medicine (Grant No. 2020-JYB-ZDGG-143-1), National Natural Science Foundation of China (Grant No. 82074545), Hospital-level Cultivation Project of the Third Affiliated Hospital of Beijing University of Chinese Medicine (Grant No. BZYSY-2022-PYQN-05), Project of Famous Doctor Inheritance Workstation of Zhang Daizhao,"3+3" Project of Beijing Traditional Chinese Medicine Inheritance (Grant No. 2007-SZ-C-12).

Competing interests None declared.

Patient consent for publication Not applicable.

Ethics approval Not applicable.

Data sharing statement All data relevant to the study are included in the article or uploaded as supplementary information.

Supplemental material This content has been supplied by the author(s). It has not been vetted by BMJ Publishing Group Limited (BMJ) and may not have been peer-reviewed. Any opinions or recommendations discussed are solely those of the author(s) and are not endorsed by BMJ. BMJ disclaims all liability and responsibility arising from any reliance placed on the content. Where the content includes any translated material, BMJ does not warrant the accuracy and reliability of the translations (including but not limited to local regulations, clinical guidelines, terminology, drug names and drug dosages), and is not responsible for any error and/or omissions arising from translation and adaptation or otherwise.

Open access This is an open access article distributed in accordance with the Creative Commons Attribution Non Commercial (CC BY-NC 4.0) license, which permits others to distribute, remix, adapt, build upon this work non-commercially, and license their derivative works on different terms, provided the original work is properly cited, appropriate credit is given, any changes made indicated, and the use is non-commercial. See: http://creativecommons.org/licenses/by-nc/4.0/.

REFERENCES

[1] Camilleri M, Chedid V, Ford A, *et al*. Gastroparesis. *Nat Rev Dis Primers* 2018;4:41.

[2] Ye Y, Yin Y, Huh S, *et al*. Epidemiology, Etiology, and Treatment of Gastroparesis: Real-World Evidence From a Large US National Claims Database. *Gastroenterology* 2022;162:109-21.e5.

[3] Schol J, Wauters L, Dickman R, *et al*. United European Gastroenterology (UEG) and European Society for Neurogastroenterology and Motility (ESNM) consensus on gastroparesis. *United European Gastroenterol J* 2021;9:287-306.

For peer review only

[4] Dong K, Yu X, Li B, *et al*. Advances in mechanisms of postsurgical gastroparesis syndrome and its diagnosis and treatment. *Chin J Dig Dis* 2006;7:76-82.

[5] Lu D, Altieri M, Yang J, *et al*. Investigating rates of reoperation or postsurgical gastroparesis following fundoplication or paraesophageal hernia repair in New York State. *Surg Endosc* 2019;33:2886-94.

[6] Stadlhuber RJ, Sherif AE, Mittal SK, *et al*. Mesh complications after prosthetic reinforcement of hiatal closure: a 28-case series. *Surg Endosc* 2009;23:1219-26.

[7] Anderson MJ, Sippey M, Marks J. Gastric Per Oral Pyloromyotomy for Post-Vagotomy-Induced Gastroparesis Following Esophagectomy. *J Gastrointest Surg* 2020;24:715-19.

[8] Hooft N, Smith M, Huang J, *et al*. Gastroparesis is common after lung transplantation and may be ameliorated by botulinum toxin-A injection of the pylorus. *J Heart Lung Transplant* 2014;33:1314-6.

[9] Grass F, Schäfer M, Cristaudi A, *et al*. Incidence and Risk Factors of Abdominal Complications After Lung Transplantation. *World J Surg* 2015;39:2274-81.

[10] Paul S, Escareno CE, Clancy K, *et al*. Gastrointestinal complications after lung transplantation. *J Heart Lung Transplant* 2009;28:475-9.

[11] Chen F, Nakamoto Y, Kondo T, *et al*. Gastroparesis after living-donor lobar lung transplantation: report of five cases. *Surg Today* 2015;45:378-82.

[12] Rodriguez J, Strong A, Haskins I, *et al*. Per-oral Pyloromyotomy (POP) for Medically Refractory Gastroparesis: Short Term Results From the First 100 Patients at a High Volume Center. *Ann Surg* 2018;268:421-30.

[13] Xu J, Chen T, Elkholy S, *et al*. Gastric Peroral Endoscopic Myotomy (G-POEM) as a Treatment for Refractory Gastroparesis: Long-Term Outcomes. *Can J Gastroenterol Hepatol* 2018;2018:6409698.

[14] Burlen J, Runnels M, Mehta M, *et al*. Efficacy of Gastric Electrical Stimulation for Gastroparesis: US/European Comparison. *Gastroenterology Res* 2018;11:349-54.

[15] Lu L, Zhang Y, Ge S, *et al*.. Evidence mapping and overview of systematic reviews of the effects of acupuncture therapies. *BMJ Open*. 2022 Jun 6;12:e056803.

[16] Sun H, Wang DH. Clinical observation on different acupuncture and moxibustion therapies for treatment of postsurgical gastroparesis syndrome. *Zhongguo Zhen Jiu* 2007;27:173-5.

[17] Sun B, Luo M, Wu S, *et al*. Acupuncture versus metoclopramide in treatment of postoperative gastroparesis syndrome in abdominal surgical patients: a randomized controlled trial. *Zhong Xi Yi Jie He Xue Bao* 2010;8:641-4.

[18] Ernst E. Acupuncture--a critical analysis. *J Intern Med* 2006;259:125-37.

[19] Yu B, Sun M, Wang Z, *et al*. Effects of Stimulating Local and Distal Acupoints on Diabetic Gastroparesis: A New Insight in Revealing Acupuncture Therapeutics. *Am J Chin Med* 2021;49:1151-64.

[20] Xing X, Jiang RL, Lei S, *et al*.. Electroacupuncture in Treatment of Acute Gastrointestinal Injury in Patients with Severe Traumatic Brain Injury: A Multicenter Randomized Controlled Trial. *Chin J Integr Med.* 2022

For peer review only

For peer review only - http://bmjopen.bmj.com/site/about/guidelines.xhtml

[21] Chen J, Liang FX, Wu S, *et al*.. Clinical research of acupuncture therapy combined with domperidone in treatment of diabetic gastroparesis of liver stagnation and spleen deficiency. *Zhen Ci Yan Jiu*. 2023;48:88-94.

[22] Güçel F, Bahar B, Demirtas C, *et al*.. Influence of acupuncture on leptin, ghrelin, insulin and cholecystokinin in obese women: a randomised, sham-controlled preliminary trial. *Acupunct Med*. 2012;30:203-7.

[23] Yang N, Yang J, Ye Y, *et al*. Electroacupuncture ameliorates intestinal inflammation by activating α7nAChR-mediated JAK2/STAT3 signaling pathway in postoperative ileus. *Theranostics* 2021;11:4078-89.

[24] Noguchi E. Acupuncture regulates gut motility and secretion via nerve reflexes. *Auton Neurosci* 2010;156:15-8.

[25] Liu J, Huang H, Xu X, *et al*.. Effects and possible mechanisms of acupuncture at ST36 on upper and lower abdominal symptoms induced by rectal distension in healthy volunteers. *Am J Physiol Regul Integr Comp Physiol*. 2012;303:R209-17.

[26] Tian L, Song S, Zhu B, *et al*.. Electroacupuncture at ST-36 Protects Interstitial Cells of Cajal via Sustaining Heme Oxygenase-1 Positive M2 Macrophages in the Stomach of Diabetic Mice. *Oxid Med Cell Longev*. 2018;2018:3987134.

[27] Song Y, Wang X, Li X, *et al*. Effect of Tiaoli Piwei needling technique on diabetic gastroparesis and transmembrane protein 16A. *Zhongguo Zhen Jiu* 2020;40:811-5.

[28] Zhang C, Huang X, Luo Y, *et al*. Observation on therapeutic effects of acupoint injection of metoclopramide for postsurgical gastroparesis syndrome. *Zhen Ci Yan Jiu* 2014;39:406-9.

[29] Qiu Y, Zhang J, Niu S. Clinical Effect of Acupuncture of 'Tong Fu Chu Man Method' Combinng Xiao Cheng Qi Decoction on Gastric Palsy Syndrome after Abdominal Surgery and Its Effect on Gastrointestinal Dynamics (In Chinese). *Xinjiang Journal of Traditional Chinese Medicine* 2020;38:20-23.

[30] Chen K, Wu Z, Wang J, *et al*. Efficacy and safety of long-term transcutaneous electroacupuncture versus sham transcutaneous electroacupuncture for delayed gastric emptying after distal gastrectomy: study protocol for a randomized, patient-assessor blinded, controlled trial. *Trials* 2022;23:189.

[31] Li H, Wen Q, Lu L, *et al*.. Transcutaneous electrical acupoint stimulation combined with electroacupuncture for rapid recovery of patients after laparotomy for gastrointestinal surgery: a study protocol for a randomised controlled trial. *BMJ Open*. 2021;11:e053309.

[32] Page M, McKenzie J, Bossuyt P, *et al*. The PRISMA 2020 statement: an updated guideline for reporting systematic reviews. *BMJ* 2021;372:n71.

[33] Qin X, Liu F. Diagnosis and treatment of postoperative gastroparesis (In Chinese). *Chinese Journal of Digestion* 2005:441-42.

[34] Camilleri M, Parkman H, Shafi M, *et al*. Clinical guideline: management of gastroparesis. *Am J Gastroenterol* 2013;108:18-37; quiz 38.

[35] Grover M, Farrugia G, Stanghellini V. Gastroparesis: a turning point in understanding and treatment. *Gut* 2019;68:2238-50.

For peer review only

[36] China Association of Chinese Medicine. Guidelines for the diagnosis and treatment of common diseases in internal medicine of TCM: symptoms. Yizhuang District, PK: *China Traditional Chinese Medicine Publishing House*:2008:179-182.

[37] Cumpston M, Li T, Page M, *et al*. Updated guidance for trusted systematic reviews: a new edition of the Cochrane Handbook for Systematic Reviews of Interventions. *Cochrane Database Syst Rev* 2019;10:ED000142.

[38] Guyatt GH, Oxman AD, Kunz R, *et al*. What is "quality of evidence" and why is it important to clinicians? *BMJ* 2008;336:995-8.

[39] Guyatt G, Oxman AD, Akl EA, *et al*. GRADE guidelines: 1. Introduction-GRADE evidence profiles and summary of findings tables. *J Clin Epidemiol* 2011;64:383-94.

[40] Chen Z, Xu F. Clinical study on the treatment of postoperative gastroparesis syndrome by acupuncture 'Three needle in stomach' (In Chinese). *Shandong Journal of Traditional Chinese Medicine* 2012;31:496-97.

[41] Chen J, He P, Zhao C, *et al*. Therapeutic Observation of Acupuncture-moxibustion for Postsurgical Gastroparesis and Effect on Gastrin and Motilin (In Chinese). *Shanghai Journal of Acupuncture and Moxibustion* 2016;35:1083-86.

[42] Dong W, Run Y, Zhao Z, *et al*. Clinical effect of warm acupuncture combined with enteral nutrition on postsurgical gastroparesis syndrome (In Chinese). *Global Traditional Chinese Medicine* 2017;10:1134-36.

[43] Huang J, Chen C, Tan C, *et al*. Effect of Acupuncture at Zusanli (ST36) for Gastroparesis Syndrome after Radical Subtotal Gastrectomy. *Shanghai Journal of Acupuncture and Moxibustion* 2020;39:1429-33.

[44] Li H, Shen H. Clinical Study on the Treatment of Postsurgical Gastroparesis Syndrome by Gastrointestinal Xia He Point (In Chinese). *Shanghai Journal of Acupuncture and Moxibustion* 2005:14-16.

[45] Lin P. Clinical study on the treatment of Gastroparesis Syndrome after abdominal operation with acupuncture of "Tong Fu Chu Man" (In Chinese). *Fujian University of Traditional Chinese Medicine*, 2017.

[46] Liu X. Clinical observation of electroacupuncture combined with Western medicine in the treatment of gastroparalysis (In Chinese). *Health for Everyone* 2017:75-76.

[47] Ma L, Zhang C, Shen Y. Acupuncture and moxibustion combined with enteral nutrition in the treatment of postsurgical gastroparesis syndrome (In Chinese). *Zhejiang Journal of Traditional Chinese Medicine* 2014;49:672.

[48] Meng Z, Dou L, Du G, *et al*. Therapeutic Observation on Electroacupuncture for Gastroparesis after Subtotal Gastrectomy (In Chinese). *Shanghai Journal of Acupuncture and Moxibustion* 2012;31:321-22.

[49] Sun Y, Sun Y. Acupuncture with Mosapride for postoperative thoracic gastric emptying disorder in esophageal cancer (In Chinese). *Journal of Hebei Traditional Chinese Medicine and Pharmacology* 2010;25:42-43.

[50] Yang Z, Hou Z, Gong D, *et al*. Clinical evaluation of electroacupuncture for functional gastric emptying disorder after abdominal surgery (In Chinese). *Journal of Hebei Traditional Chinese Medicine and Pharmacology* 2010;25:32-33.

For peer review only - http://bmjopen.bmj.com/site/about/guidelines.xhtml

[51] Zhang L, Guan L, Zhang Y, *et al*. Clinical observation of Dongyuan acupuncture combined with mosapride citrate for treatment of gastroparesis after gynecological abdominal surgery (In Chinese). *Journal of Guangzhou University of Traditional Chinese Medicine* 2021;38:1645-50.

[52] Zhang J, Zhao J, Jiao B, *et al*. Clinical observation on the treatment of postoperative gastroparesis of gastric cancer by acupuncture combined with conventional western medicine (In Chinese). *China's Naturopathy* 2022;30:64-66.

[53] Zhao W, Jiang X, Huang J. Clinical observation on the effects of warm acupuncture and moxinbustion on gastroparesis after tumor operation (In Chinese). *China Journal of Traditional Chinese Medicine and Pharmacy* 2021;36:4379-82.

[54] Wang Y, Yang H, Liu Y, *et al*. Acupuncture in the Treatment of Postsurgical Gastro-paresis Syndrome: Clinical Observation of 22 cases (In Chinese). *Journal of Clinical Acupuncture and Moxibustion* 2013;29:10-13.

[55] Al-Missri MZ, Jialal I. StatPearls [Internet]: Physiology, Motilin. Treasure Island, FL: *StatPearls Publishing* 2022

[56] Zhang Z, Wu S, Wang B, *et al*.. Sphincter of Oddi hypomotility and its relationship with duodenal-biliary reflux, plasma motilin and serum gastrin. *World J Gastroenterol*. 2008;14:4077-81.

[57] Prosapio JG, Sankar P, Jialal I. StatPearls [Internet]： Physiology, Gastrin. Treasure Island, FL: *StatPearls Publishing* 2022

[58] Luo G, Cao Y, Zhang L, *et al*.. Diagnostic value and influencing factors of gastric juice volume in gastroparesis syndrome after abdominal surgery (with 19 case reports) (In Chinese). *Chin J Mod Med* 2012;22:69-71.

[59] Sun H. Effects of different gastrointestinal decompression methods on functional recovery of patients with radical gastric cancer surgery (In Chinese). *The Practical Journal of Cancer* 2015;30:747-749.

[60] Chen HY, Kuo TB, Shaw FZ, *et al*. Sleep-related vagotonic effect of zolpidem in rats. *Psychopharmacology* 2005;181:270-9.

[61] Rao A, Camilleri M. Review article: metoclopramide and tardive dyskinesia. *Aliment Pharmacol Ther* 2010;31:11-9.

[62] Cullen PT, Campbell FC. Cisapride in postoperative gastroparesis. *Lancet* 1987;1:47.

[63] Berkowitz N, Schulman LL, McGregor C, *et al*. Gastroparesis after lung transplantation. Potential role in postoperative respiratory complications. *Chest* 1995;108:1602-7.

[64] Kawamura E, Enomoto M, Kotani K, *et al*. Effect of mosapride citrate on gastric emptying in interferon-induced gastroparesis. *Dig Dis Sci* 2012;57:1510-6.

[65] Ogasawara N, Yamaguchi Y, Takahama T, *et al*. Radiofrequency catheter ablation-induced gastroparesis successfully treated with administration of mosapride citrate: two case reports. *Clin J Gastroenterol* 2020;13:182-85.

[66] Carbone F, Van den Houte K, Clevers E, *et al*. Prucalopride in Gastroparesis: A Randomized Placebo-Controlled Crossover Study. *Am J Gastroenterol* 2019;114:1265-74.

For peer review only - http://bmjopen.bmj.com/site/about/guidelines.xhtml

[67] Richards RD, Davenport K, McCallum RW. The treatment of idiopathic and diabetic gastroparesis with acute intravenous and chronic oral erythromycin. *Am J Gastroenterol* 1993;88:203-7.

[68] Talley NJ, Verlinden M, Snape W, *et al*. Failure of a motilin receptor agonist (ABT-229) to relieve the symptoms of functional dyspepsia in patients with and without delayed gastric emptying: a randomized double-blind placebo-controlled trial. *Aliment Pharmacol Ther* 2000;14:1653-61.

[69] Garg R, Mohan BP, Aggarwal M, *et al*. Peroral Pyloromytomy is Effective and Safe for Postsurgical Gastroparesis. *J Gastrointest Surg* 2020;24:1417-20.

[70] Takahashi T. Acupuncture for functional gastrointestinal disorders. *J Gastroenterol* 2006;41:408-17.

[71] Yin J, Chen J, Chen J. Ameliorating effects and mechanisms of electroacupuncture on gastric dysrhythmia, delayed emptying, and impaired accommodation in diabetic rats. *Am J Physiol Gastrointest Liver Physiol* 2010;298:G563-70.

[72] Lu M, Yu Z, He Y, *et al*. Electroacupuncture at ST36 modulates gastric motility via vagovagal and sympathetic reflexes in rats. *World J Gastroenterol* 2019;25:2315-26.

[73] Yue G, Yuan X, Ge X, *et al*. Effect of electroacupuncture at different acupoints in treating rapid gastric emptying: Zusanli (ST36) plus Zhongwan (CV12) versus Zhongwan (CV12) alone. *J Tradit Chin Med* 2021;41:326-30.

[74] Cui Y, Wang L, Shi G, *et al*. Electroacupuncture alleviates cisplatin-induced nausea in rats. *Acupunct Med* 2016;34:120-6.

[75] Garcia G, Ribeiro R, Faria Santos I, *et al*. Electrical Stimulation of PC 6 to Control Chemotherapy-Induced Nausea and Vomiting in Patients with Cancer: A Systematic Review and Meta-Analysis. *Med Acupunct* 2021;33:22-44.

[76] Zhang Y, Zhang C, Yan M, *et al*. The effectiveness of PC6 acupuncture in the prevention of postoperative nausea and vomiting in children: A systematic review and meta-analysis. *Paediatr Anaesth* 2020;30:552-63.

[77] Stott A. Examining the efficacy of stimulating the PC6 wrist acupuncture point for preventing postoperative nausea and vomiting: A Cochrane review summary. *Int J Nurs Stud* 2016;64:139-41.

[78] Lu M, Chen C, Li W, *et al*. EA at PC6 Promotes Gastric Motility: Role of Brainstem Vagovagal Neurocircuits. *Evid Based Complement Alternat Med* 2019;2019:7457485.

[79] Gao L, Chen B, Zhang Q, *et al*. Acupuncture with different acupoint combinations for chemotherapy-induced nausea and vomiting: study protocol for a randomized controlled trial. *BMC Complement Altern Med* 2016;16:441.

Legends:

Figure 1 PRISMA flow diagram of screening process.

Figure 2 Risk of bias summary and risk of bias graph.

Figure 3 Forest plot of the total effective rate (A) and the recovery rate (B).

For peer review only - http://bmjopen.bmj.com/site/about/guidelines.xhtml

Figure 4 Forest plot of the serum motilin (A), the serum gastrin (B) and the gastric juice volume (C).

Figure 5 Forest plot of days of gastrointestinal motility recovery(A), days of gastrointestinal decompression(B), days to resumption of diet for the first time(C) and clinical symptom score(D).

For peer review only

For peer review only - http://bmjopen.bmj.com/site/about/guidelines.xhtml

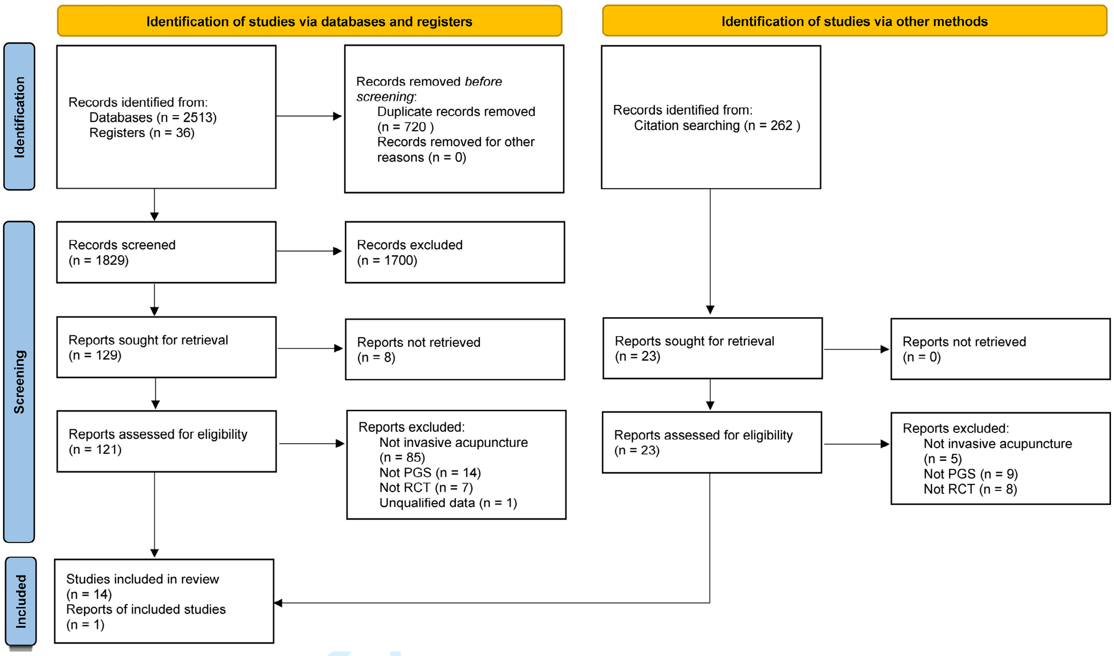

Figure 1 PRISMA flow diagram of screening process.

For peer review only

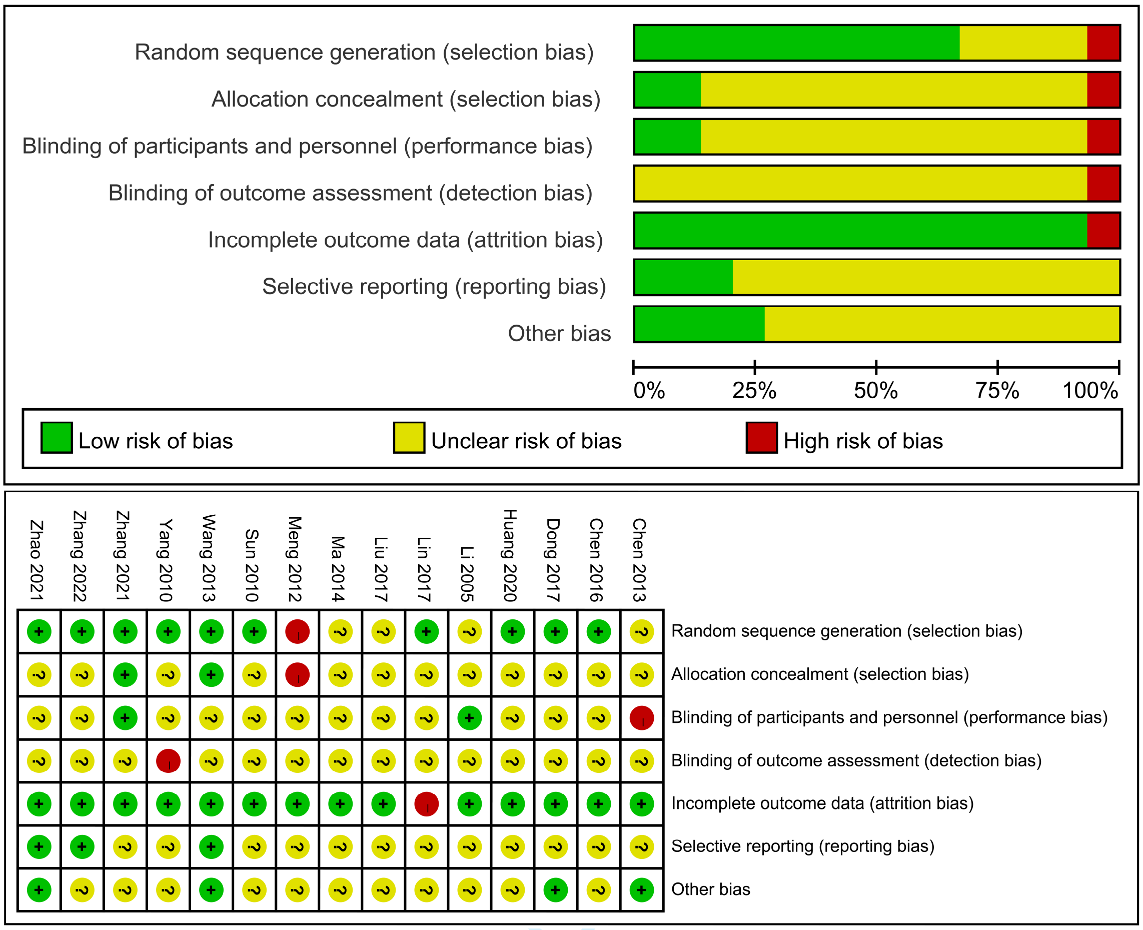

Figure 2 Risk of bias summary and risk of bias graph.

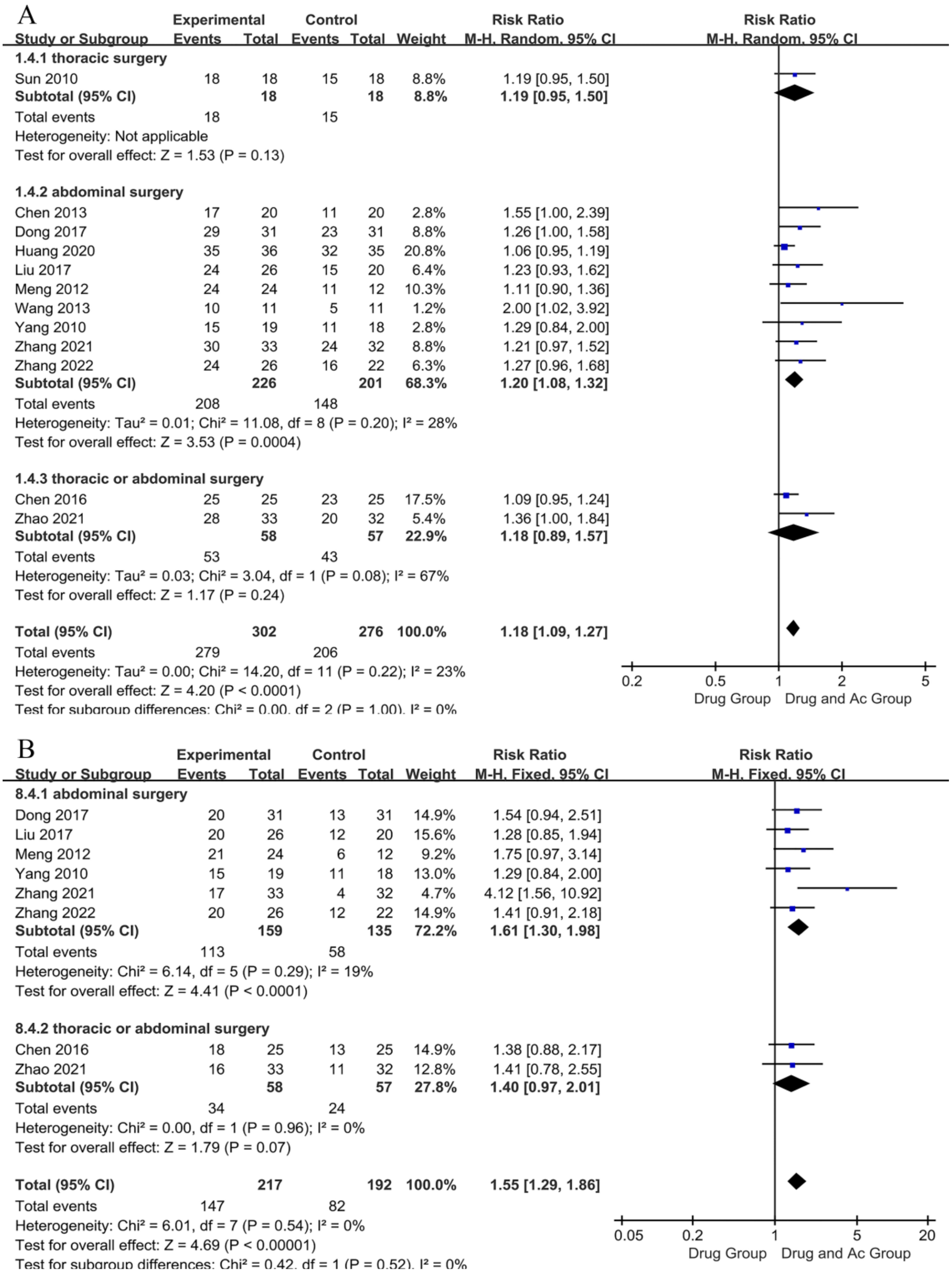

Figure 3 Forest plot of the total effective rate (A) and the recovery rate (B).

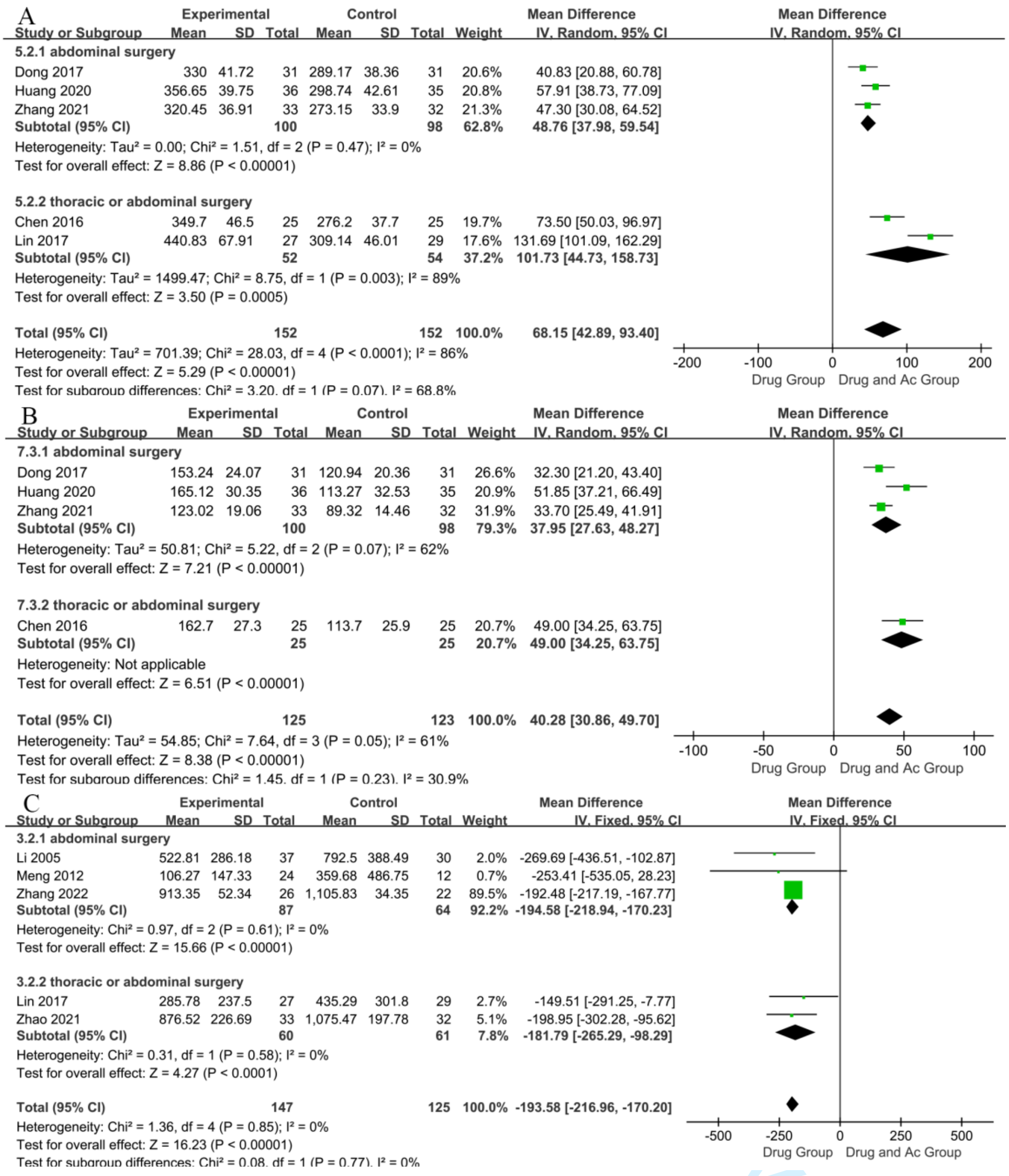

Figure 4 Forest plot of the serum motilin (A), the serum gastrin (B) and the gastric juice volume (C).

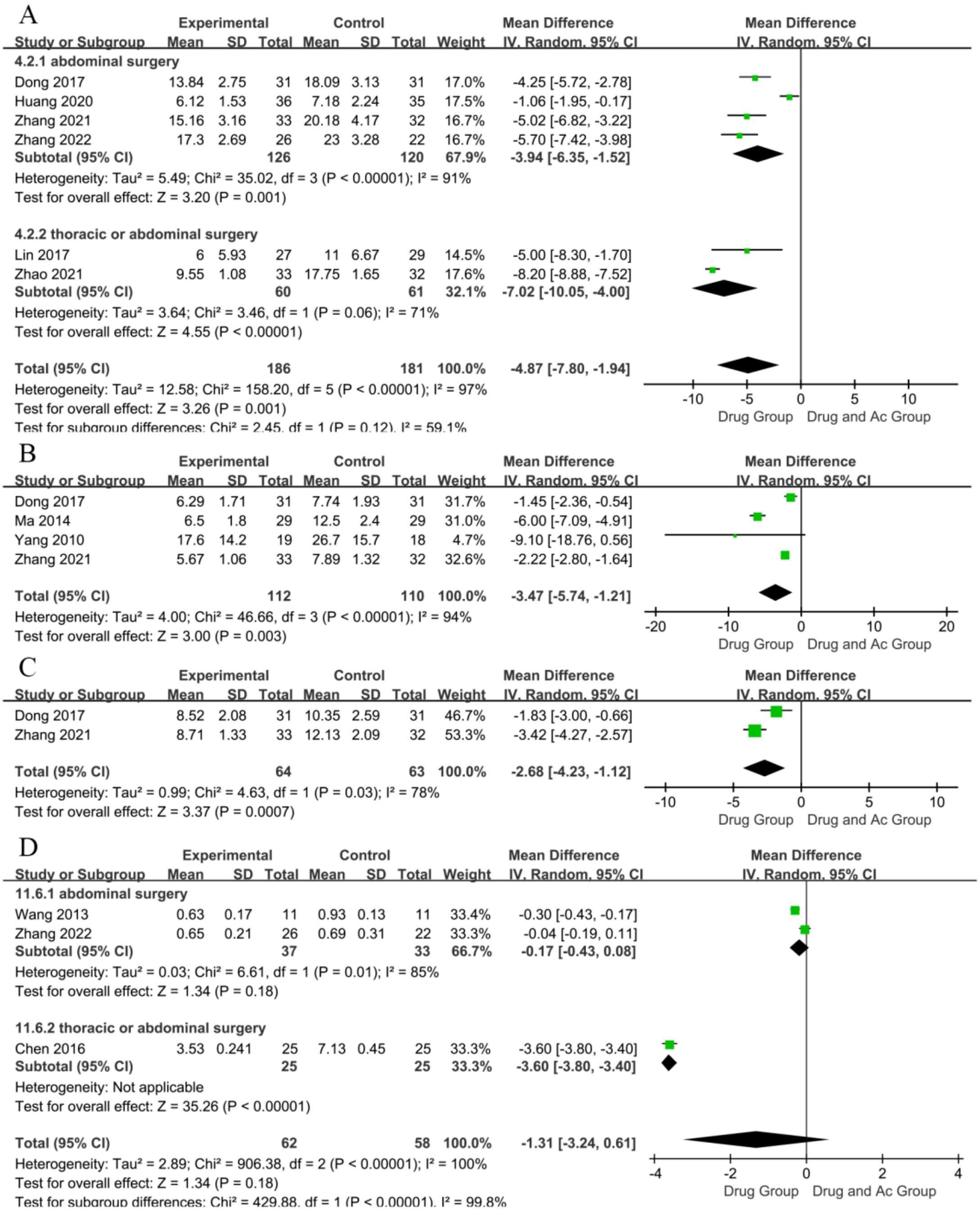

Figure 5 Forest plot of days to gastrointestinal motility recovery(A), days to gastrointestinal decompression(B), days to resumption of diet for the first time(C) and clinical symptom score(D).

PRISMA 2020 Checklist

Section and Topic	Item #	Checklist item	Location where item is reported
**TITLE**			
Title	1	Identify the report as a systematic review.	1
**ABSTRACT**			
Abstract	2	See the PRISMA 2020 for Abstracts checklist.	2
**INTRODUCTION**			
Rationale	3	Describe the rationale for the review in the context of existing knowledge.	3
Objectives	4	Provide an explicit statement of the objective(s) or question(s) the review addresses.	4
**METHODS**			
Eligibility criteria	5	Specify the inclusion and exclusion criteria for the review and how studies were grouped for the syntheses.	4, 5
Information sources	6	Specify all databases, registers, websites, organisations, reference lists and other sources searched or consulted to identify studies. Specify the date when each source was last searched or consulted.	4
Search strategy	7	Present the full search strategies for all databases, registers and websites, including any filters and limits used.	4
Selection process	8	Specify the methods used to decide whether a study met the inclusion criteria of the review, including how many reviewers screened each record and each report retrieved, whether they worked independently, and if applicable, details of automation tools used in the process.	5
Data collection process	9	Specify the methods used to collect data from reports, including how many reviewers collected data from each report, whether they worked independently, any processes for obtaining or confirming data from study investigators, and if applicable, details of automation tools used in the process.	5
Data items	10a	List and define all outcomes for which data were sought. Specify whether all results that were compatible with each outcome domain in each study were sought (e.g. for all measures, time points, analyses), and if not, the methods used to decide which results to collect.	5
	10b	List and define all other variables for which data were sought (e.g. participant and intervention characteristics, funding sources). Describe any assumptions made about any missing or unclear information.	5
Study risk of bias assessment	11	Specify the methods used to assess risk of bias in the included studies, including details of the tool(s) used, how many reviewers assessed each study and whether they worked independently, and if applicable, details of automation tools used in the process.	5
Effect measures	12	Specify for each outcome the effect measure(s) (e.g. risk ratio, mean difference) used in the synthesis or presentation of results.	6
Synthesis methods	13a	Describe the processes used to decide which studies were eligible for each synthesis (e.g. tabulating the study intervention characteristics and comparing against the planned groups for each synthesis (item #5)).	6
	13b	Describe any methods required to prepare the data for presentation or synthesis, such as handling of missing summary statistics, or data conversions.	6
	13c	Describe any methods used to tabulate or visually display results of individual studies and syntheses.	5
	13d	Describe any methods used to synthesize results and provide a rationale for the choice(s). If meta-analysis was performed, describe the model(s), method(s) to identify the presence and extent of statistical heterogeneity, and software package(s) used.	5, 6
	13e	Describe any methods used to explore possible causes of heterogeneity among study results (e.g. subgroup analysis, meta-regression).	6
	13f	Describe any sensitivity analyses conducted to assess robustness of the synthesized results.	6
Reporting bias assessment	14	Describe any methods used to assess risk of bias due to missing results in a synthesis (arising from reporting biases).	5
Certainty assessment	15	Describe any methods used to assess certainty (or confidence) in the body of evidence for an outcome.	6

For peer review only - http://bmjopen.bmj.com/site/about/guidelines.xhtml

PRISMA 2020 Checklist

Section and Topic	Item #	Checklist item	Location where item is reported
**RESULTS**			
Study selection	16a	Describe the results of the search and selection process, from the number of records identified in the search to the number of studies included in the review, ideally using a flow diagram.	6
	16b	Cite studies that might appear to meet the inclusion criteria, but which were excluded, and explain why they were excluded.	6
Study characteristics	17	Cite each included study and present its characteristics.	6
Risk of bias in studies	18	Present assessments of risk of bias for each included study.	7
Results of individual studies	19	For all outcomes, present, for each study: (a) summary statistics for each group (where appropriate) and (b) an effect estimate and its precision (e.g. confidence/credible interval), ideally using structured tables or plots.	7-10
Results of syntheses	20a	For each synthesis, briefly summarise the characteristics and risk of bias among contributing studies.	7-10
	20b	Present results of all statistical syntheses conducted. If meta-analysis was done, present for each the summary estimate and its precision (e.g. confidence/credible interval) and measures of statistical heterogeneity. If comparing groups, describe the direction of the effect.	7-10
	20c	Present results of all investigations of possible causes of heterogeneity among study results.	7-10
	20d	Present results of all sensitivity analyses conducted to assess the robustness of the synthesized results.	7-10
Reporting biases	21	Present assessments of risk of bias due to missing results (arising from reporting biases) for each synthesis assessed.	10
Certainty of evidence	22	Present assessments of certainty (or confidence) in the body of evidence for each outcome assessed.	7
**DISCUSSION**			
Discussion	23a	Provide a general interpretation of the results in the context of other evidence.	11-12
	23b	Discuss any limitations of the evidence included in the review.	13
	23c	Discuss any limitations of the review processes used.	13
	23d	Discuss implications of the results for practice, policy, and future research.	13
**OTHER INFORMATION**			
Registration and protocol	24a	Provide registration information for the review, including register name and registration number, or state that the review was not registered.	2
	24b	Indicate where the review protocol can be accessed, or state that a protocol was not prepared.	2
	24c	Describe and explain any amendments to information provided at registration or in the protocol.	NA
Support	25	Describe sources of financial or non-financial support for the review, and the role of the funders or sponsors in the review.	14
Competing interests	26	Declare any competing interests of review authors.	14
Availability of data, code and other materials	27	Report which of the following are publicly available and where they can be found: template data collection forms; data extracted from included studies; data used for all analyses; analytic code; any other materials used in the review.	14

From: Page MJ, McKenzie JE, Bossuyt PM, Boutron I, Hoffmann TC, Mulrow CD, et al. The PRISMA 2020 statement: an updated guideline for reporting systematic reviews. BMJ 2021;372:n71. doi: 10.1136/bmj.n71

For peer review only - http://bmjopen.bmj.com/site/about/guidelines.xhtml

For more information, visit: http://www.prisma-statement.org/

Supplemental Table 2 Search strategy

Database	#	Search syntax
PubMed	1	(acupuncture[MeSH Terms]) OR (acupuncture therapy[MeSH Terms]) OR (acupoint[MeSH Terms]) OR (electroacupuncture[MeSH Terms]) OR (electric acupuncture[MeSH Terms]) OR (auriculotherapy[MeSH Terms]) OR (auricular acupuncture[MeSH Terms]) OR (elongated needle[MeSH Terms]) OR (scalp acupuncture[MeSH Terms]) OR (hand acupuncture[MeSH Terms]) OR (manual acupuncture[MeSH Terms]) OR (body acupuncture[MeSH Terms]) OR (warm* acupuncture[MeSH Terms]) OR (warm* needl*[MeSH Terms]) OR (fire needl*[MeSH Terms]) OR (trigger point[MeSH Terms]) OR (meridian[MeSH Terms]) OR (needl*[MeSH Terms]) OR (abdominal acupuncture[MeSH Terms]) OR (float* needl*[MeSH Terms]) OR (embedded acupuncture[MeSH Terms]) OR (intradermal acupuncture[MeSH Terms]) OR (needl*[MeSH Terms]) OR (acupuncture*[Title/Abstract]) OR (acupuncture therapy[Title/Abstract]) OR (acupoint*[Title/Abstract]) OR (electroacupuncture*[Title/Abstract]) OR (electric acupuncture*[Title/Abstract]) OR (auriculotherapy[Title/Abstract]) OR (auricular acupuncture*[Title/Abstract]) OR (elongated needl*[Title/Abstract]) OR (scalp acupuncture*[Title/Abstract]) OR (hand acupuncture*[Title/Abstract]) OR (manual acupuncture*[Title/Abstract]) OR (body acupuncture*[Title/Abstract]) OR (warm* acupuncture*[Title/Abstract]) OR (warm* needl*[Title/Abstract]) OR (fire needl*[Title/Abstract]) OR (trigger point*[Title/Abstract]) OR (meridian*[Title/Abstract]) OR (needl*[Title/Abstract]) OR (abdominal acupuncture*[Title/Abstract]) OR (float* needl*[Title/Abstract]) OR (embedded acupuncture*[Title/Abstract]) OR (intradermal acupuncture*[Title/Abstract]) OR (electro-acupuncture*[Title/Abstract]) OR (micro-puncture*[Title/Abstract]) OR (needl* embedding[Title/Abstract])
	2	(surgery[MeSH Terms]) OR (operation[MeSH Terms]) OR (postoperative[MeSH Terms]) OR (surger*[Title/Abstract]) OR (operation*[Title/Abstract]) OR (postoperati*[Title/Abstract]) OR (postsurgical[Title/Abstract]) OR (excision*[Title/Abstract]) OR (resecti*[Title/Abstract])
	3	(gastroparesis[MeSH Terms]) OR (gastropareses[MeSH Terms]) OR (gastric stasis[MeSH Terms]) OR (gastric stases[MeSH Terms]) OR (stasis, gastric[MeSH Terms]) OR (gastr* paralysis[MeSH Terms]) OR (delay* gastric emptying[MeSH Terms]) OR (stomach palsy[MeSH Terms]) OR (gastr* motility disorder[MeSH Terms]) OR (gastroparesis[Title/Abstract]) OR (stasis, gastric[Title/Abstract]) OR (gastr* paralysis[Title/Abstract]) OR (delay* gastric emptying[Title/Abstract]) OR (stomach palsy[Title/Abstract]) OR (gastr* motility disorder[Title/Abstract]) OR (gastric stasis[Title/Abstract]) OR (gastroparesis syndrome[Title/Abstract]) OR (impaired gastric accommodation[Title/Abstract]) OR (stomach paresis[Title/Abstract])
	4	(Randomized controlled trial) or ((controlled) and (study or design or trial)) or (random*) or (intermethod comparison) or (placebo) or (compar*) or ((singl* or doubl* or trebl* or tripl*) and (blind* or mask*)) or (cross over) or (trial[Title/Abstract])
	5	#1 AND #2 AND #3 AND #4
Embase	1	'acupuncture'/exp OR 'needle'/exp OR 'electroacupuncture'/exp OR 'auricular acupuncture'/exp OR 'acupuncture needle'/exp OR 'scalp acupuncture'/exp OR 'manual acupuncture'/exp OR 'body acupuncture'/exp OR 'warm acupuncture'/exp OR 'meridian'/exp OR 'abdominal acupuncture'/exp OR 'trigger point'/exp OR 'dry needling'/exp OR 'acupuncture*':ti,ab,kw OR 'needl*':ti,ab,kw OR 'electroacupuncture*':ti,ab,kw OR 'auricular acupuncture*':ti,ab,kw OR 'acupuncture* needl*':ti,ab,kw OR 'scalp acupuncture*':ti,ab,kw OR 'manual acupuncture*':ti,ab,kw OR 'body acupuncture*':ti,ab,kw OR 'warm* acupuncture*':ti,ab,kw OR 'meridian*':ti,ab,kw OR 'abdominal acupuncture*':ti,ab,kw OR 'trigger point*':ti,ab,kw OR 'dry needl*':ti,ab,kw OR 'acupuncture* therap*':ti,ab,kw OR 'acupoint*':ti,ab,kw OR 'electric

For peer review only

		acupuncture*':ti,ab,kw OR 'electro-acupuncture*':ti,ab,kw OR 'auricular-acupuncture*':ti,ab,kw OR 'auriculotherap*':ti,ab,kw OR 'elongated needl*':ti,ab,kw OR 'micro-puncture*':ti,ab,kw OR 'hand acupuncture*':ti,ab,kw OR 'warm* needl*':ti,ab,kw OR 'fire needl*':ti,ab,kw OR 'float* needl*':ti,ab,kw OR 'embedd* acupuncture*':ti,ab,kw OR 'needl* embedd*':ti,ab,kw OR 'intradermal acupuncture*':ti,ab,kw
	2	'surgery'/exp OR 'excision'/exp OR 'surger*':ti,ab,kw OR 'excision*':ti,ab,kw OR 'operation*':ti,ab,kw OR 'postoperation*':ti,ab,kw OR 'postsurgical*':ti,ab,kw OR 'postoperative*':ti,ab,kw OR 'resection*':ti,ab,kw
	3	'stomach paresis'/exp OR 'gastric stasis'/exp OR 'stomach emptying'/exp OR 'gastric dysmotility'/exp OR 'stomach paresis':ti,ab,kw OR 'gastric stasis':ti,ab,kw OR 'stomach emptying':ti,ab,kw OR 'gastric dysmotility':ti,ab,kw OR 'gastroparesis':ti,ab,kw OR 'gastropareses':ti,ab,kw OR 'stasis, gastric':ti,ab,kw OR 'gastr* paralysis':ti,ab,kw OR 'gastroparesis syndrome':ti,ab,kw OR 'delay* gastr* empty*':ti,ab,kw OR 'disturbance of gastric emptying':ti,ab,kw OR 'gastr* motility disorder':ti,ab,kw OR 'impaired gastric accommodation':ti,ab,kw OR 'stomach paralysis':ti,ab,kw
	4	'Randomized controlled trial' or 'controlled adj7 (study or design or trial)' or 'random*' or 'intermethod comparison' or 'placebo' or 'compar*' or '(singl* or doubl* or trebl* or tripl*) adj (blind* or mask*)' or '(cross-over or crossover or cross over)' or 'trial':ti,kw,ab
	5	#1 AND #2 AND #3 AND #4
CENTRAL	1	(MeSH descriptor:[Acupuncture OR 'Acupuncture Therapy' OR 'Acupuncture Points' OR 'Needles' OR 'Auriculotherapy' OR 'Acupuncture, Ear' OR 'Electroacupuncture' OR 'Trigger Points' OR 'Meridians' OR 'Dry Needling'] explode all trees) OR (acupuncture* OR needl* OR electroacupuncture* OR 'auricular acupuncture*' OR 'acupuncture* needl*' OR 'scalp acupuncture*' OR 'manual acupuncture*' OR 'body acupuncture*' OR 'warm* acupuncture*' OR meridian* OR 'abdominal acupuncture*' OR 'trigger point*' OR 'dry needl*' OR 'acupuncture* therap*' OR acupoint* OR 'electric acupuncture*' OR electro-acupuncture* OR auricular-acupuncture* OR auriculotherap* OR 'elongated needl*' OR 'micro-puncture*' OR 'hand acupuncture*' OR 'warm* needl*' OR 'fire needl*' OR 'float* needl*' OR 'embedd* acupuncture*' OR 'needl* embedd*' OR 'intradermal acupuncture*'):ti,ab,kw
	2	MeSH descriptor:[General Surgery] explode all trees OR (surger* OR excision* OR operation* OR postoperation* OR postsurgical* OR postoperative* OR resection*):ti,ab,kw
	3	MeSH descriptor:[Gastroparesis] explode all trees OR (gastroparesis OR gastropareses OR gastric stasis OR gastric stases OR 'gastr* paralysis' OR 'delay* gastric emptying' OR 'disturbance of gastric emptying' OR 'weakness of gastric emptying' OR 'weakness of stomach' OR 'stomach palsy' OR 'gastr* motility disorder' OR 'gastroparesis syndrome' OR 'impaired gastric accommodation' OR 'stomach paresis'):ti,ab,kw
	4	#1 AND #2 AND #3
Medline (Ovid) (1946-)	1	exp Acupuncture/ or exp Acupuncture Therapy/ or exp Acupuncture Points/ or exp Needles/ or exp electroacupuncture/ or exp auriculotherapy/ or exp Acupuncture, Ear/ or exp Trigger Points/ or exp Meridians/ or exp dry needling/ OR (acupuncture* OR acupuncture therapy OR acupoint* OR electroacupuncture* OR electric acupuncture* OR auriculotherapy OR auricular acupuncture* OR elongated needl* OR scalp acupuncture* OR hand acupuncture* OR manual acupuncture* OR body acupuncture* OR warm* acupuncture* OR warm* needl* OR fire needl* OR trigger point* OR meridian* OR needl* OR abdominal acupuncture* OR float* needl* OR embedded acupuncture* OR intradermal acupuncture* OR needl* OR electro-acupuncture* OR micro-puncture* OR needl* embedding).ab,kw,ti.
	2	exp General Surgery/ or exp Postoperative Complications/ or exp "Postoperative Nausea and Vomiting"/ or exp Postoperative Period/ or (surger* or operation* or postoperati* or postsurgical

		or postoperati* or excision* or resecti*).ab,kw,ti.
	3	exp Gastroparesis/ or exp Gastrointestinal Motility/ or exp Gastric Emptying/ or (gastroparesis or stasis, gastric or delay* gastric emptying or disturbance of gastric emptying or weakness of gastric emptying or weakness of stomach or stomach palsy or gastr* motility disorder or gastric stasis or gastroparesis syndrome or impaired gastric accommodation or stomach paresis).ab,kw,ti.
	4	((randomised controlled trial or controlled clinical trial or pragmatic clinical trial).pt. or (random* or placebo or trial or groups).ti,ab. or drug therapy.fs.) not (animals not (humans and animals)).sh.
	5	#1 AND #2 AND #3 AND #4
Web of Science	1	TS=(acupuncture* OR acupuncture therapy OR acupoint* OR electroacupuncture* OR electric acupuncture* OR auriculotherapy OR auricular acupuncture* OR elongated needl* OR scalp acupuncture* OR hand acupuncture* OR manual acupuncture* OR body acupuncture* OR warm* acupuncture* OR warm* needl* OR fire needl* OR trigger point* OR meridian* OR needl* OR abdominal acupuncture* OR float* needl* OR embedded acupuncture* OR intradermal acupuncture* OR electro-acupuncture* OR micro-puncture* OR needl* embedding OR dry needl*)
	2	TS=(surger* OR operation* OR postoperati* OR postsurgical OR excision* OR resecti*)
	3	TS=(gastroparesis OR gastroparesis OR gastric stasis OR gastric stases OR stasis, gastric OR stases, gastric OR gastr* paralysis OR delay* gastric emptying OR disturbance of gastric emptying OR weakness of gastric emptying OR weakness of stomach OR stomach palsy OR gastr* motility disorder OR gastroparesis syndrome OR impaired gastric accommodation OR stomach paresis)
	4	ALL=((Randomized controlled trial) or ((controlled) "near/7" (study or design or trial)) or (random*) or (intermethod comparison) or (placebo) or (compar*) or ((singl* or doubl* or trebl* or tripl*) "near" (blind* or mask*)) or (cross over)) or TS=(trial)
	5	#1 AND #2 AND #3 AND #4
EBSCO	1	(SU (acupuncture* OR “acupuncture therapy” OR acupoint* OR electroacupuncture* OR “electric acupuncture*” OR auriculotherapy OR “auricular acupuncture*” OR “elongated needl*” OR “scalp acupuncture*” OR “hand acupuncture*” OR “manual acupuncture*” OR “body acupuncture*” OR “warm* acupuncture*” OR “warm* needl*” OR “fire needl*” OR “trigger point*” OR meridian* OR needl* OR “abdominal acupuncture*” OR “float* needl*” OR “embedded acupuncture*” OR “intradermal acupuncture*” OR electro-acupuncture* OR micro-puncture* OR “needl* embedding” OR “dry needl*”)) OR (TI (acupuncture* OR “acupuncture therapy” OR acupoint* OR electroacupuncture* OR “electric acupuncture*” OR auriculotherapy OR “auricular acupuncture*” OR “elongated needl*” OR “scalp acupuncture*” OR “hand acupuncture*” OR “manual acupuncture*” OR “body acupuncture*” OR “warm* acupuncture*” OR “warm* needl*” OR “fire needl*” OR “trigger point*” OR meridian* OR needl* OR “abdominal acupuncture*” OR “float* needl*” OR “embedded acupuncture*” OR “intradermal acupuncture*” OR electro-acupuncture* OR micro-puncture* OR “needl* embedding” OR “dry needl*”)) OR (AB (acupuncture* OR “acupuncture therapy” OR acupoint* OR electroacupuncture* OR “electric acupuncture*” OR auriculotherapy OR “auricular acupuncture*” OR “elongated needl*” OR “scalp acupuncture*” OR “hand acupuncture*” OR “manual acupuncture*” OR “body acupuncture*” OR “warm* acupuncture*” OR “warm* needl*” OR “fire needl*” OR “trigger point*” OR meridian* OR needl* OR “abdominal acupuncture*” OR “float* needl*” OR “embedded acupuncture*” OR “intradermal acupuncture*” OR electro-acupuncture* OR micro-puncture* OR “needl* embedding” OR “dry needl*”))

For peer review only

	2	(SU (surger* OR operation* OR postoperati* OR postsurgical OR excision* OR resecti*) OR (TI (surger* OR operation* OR postoperati* OR postsurgical OR excision* OR resecti*)) OR (AB (surger* OR operation* OR postoperati* OR postsurgical OR excision* OR resecti*))
	3	(SU (gastroparesis OR gastropareses OR gastric stasis OR gastric stases OR "gastr* paralysis" OR "delay* gastric emptying" OR "disturbance of gastric emptying" OR "weakness of gastric emptying" OR "weakness of stomach" OR "stomach palsy" OR "gastr* motility disorder" OR "gastroparesis syndrome" OR "impaired gastric accommodation" OR "stomach paresis")) OR (TI (gastroparesis OR gastropareses OR gastric stasis OR gastric stases OR "gastr* paralysis" OR "delay* gastric emptying" OR "disturbance of gastric emptying" OR "weakness of gastric emptying" OR "weakness of stomach" OR "stomach palsy" OR "gastr* motility disorder" OR "gastroparesis syndrome" OR "impaired gastric accommodation" OR "stomach paresis")) OR (AB (gastroparesis OR gastropareses OR gastric stasis OR gastric stases OR "gastr* paralysis" OR "delay* gastric emptying" OR "disturbance of gastric emptying" OR "weakness of gastric emptying" OR "weakness of stomach" OR "stomach palsy" OR "gastr* motility disorder" OR "gastroparesis syndrome" OR "impaired gastric accommodation" OR "stomach paresis"))
	4	(TX "Randomized controlled trial" OR "controlled N7 "study OR design or trial"" OR "random*" OR "intermethod comparison" OR "placebo" OR "compar*" OR ""singl* OR doubl* OR trebl* OR tripl*" N "blind* OR mask*"" OR "cross over") OR TI trial OR AB trial
	5	#1 AND #2 AND #3 AND #4
Scopus	1	TITLE-ABS-KEY (acupuncture* OR "acupuncture therapy" OR acupoint* OR electroacupuncture* OR "electric acupuncture*" OR musicotherapy OR "auricular acupuncture*" OR "elongated needl*" OR "scalp acupuncture*" OR "hand acupuncture*" OR "manual acupuncture*" OR "body acupuncture*" OR "warm* acupuncture*" OR "warm* needl*" OR "fire needl*" OR "trigger point*" OR meridian* OR needl* OR "abdominal acupuncture*" OR "float* needl*" OR "embedded acupuncture*" OR "intradermal acupuncture*" OR electro-acupuncture* OR micro-puncture* OR "needl* embedding" OR "dry needl*")
	2	TITLE-ABS-KEY (surger* OR operation* OR postoperati* OR postsurgical OR excision* OR resecti*)
	3	TITLE-ABS-KEY (gastroparesis OR gastroparesis OR gastric AND stasis OR gastric AND stases OR "gastr* paralysis" OR "delay* gastric emptying" OR "disturbance off gastric emptying" OR "weakness off gastric emptying" OR "weakness off stomach" OR "stomach palsy" OR "gastr* motility disorder" OR "gastroparesis syndrome" OR "impaired gastric accommodation" OR "stomach paresis")
	4	ALL ("Randomized controlled trial" OR "controlled W/7 " study OR design OR trial "" OR "random*" OR "intermeshed comparison" OR "placebo" OR "compar*" OR "" singl* OR doubl* OR trebl* OR tripl* " W " blind* OR mask* "" OR "cross over") OR TITLE-ABS-KEY (trial)
	5	#1 AND #2 AND #3 AND #4
OpenGrey	1	acupuncture* OR "acupuncture therapy" OR acupoint* OR electroacupuncture* OR "electric acupuncture*" OR auriculotherapy OR "auricular acupuncture*" OR "elongated needl*" OR "scalp acupuncture*" OR "hand acupuncture*" OR "manual acupuncture*" OR "body acupuncture*" OR "warm* acupuncture*" OR "warm* needl*" OR "fire needl*" OR "trigger point*" OR meridian* OR needl* OR "abdominal acupuncture*" OR "float* needl*" OR "embedded acupuncture*" OR "intradermal acupuncture*" OR electro-acupuncture* OR micro-puncture* OR "needl* embedding" OR "dry needl*"
	2	gastroparesis OR gastropareses OR gastric stasis OR gastric stases OR "gastr* paralysis" OR "delay* gastric emptying" OR "disturbance of gastric emptying" OR "weakness of gastric

For peer review only

		emptying" OR "weakness of stomach" OR "stomach palsy" OR"gastr* motility disorder" OR "gastroparesis syndrome" OR "impaired gastric accommodation" OR "stomach paresis"
	3	#1 AND #2
CNKI	1	术后 + 手术 + 切除
	2	胃瘫综合征 + 胃瘫 + 胃轻瘫 + 胃排空延迟 + 胃排空障碍 + 胃排空无力 + 胃无力 + 胃麻痹 + 胃动力障碍
	3	针灸 + 针刺 + 针法 + 刺法 + 穴位 + 温针灸 + 温针 + 电针 + 腹针 + 浮针 + 揿针 + 皮内针 + 埋针 + 火针 + 毫针 + 热针 + 耳针 + 头皮针 + 头针
	4	#1 AND #2 AND #3
Wanfang Database	1	术后 or 手术 or 切除
	2	胃瘫综合征 or 胃瘫 or 胃轻瘫 or 胃排空延迟 or 胃排空障碍 or 胃排空无力 or 胃无力 or 胃麻痹 or 胃动力障碍
	3	针灸 or 针刺 or 针法 or 刺法 or 穴位 or 温针灸 or 温针 or 电针 or 腹针 or 浮针 or 揿针 or 皮内针 or 埋针 or 火针 or 毫针 or 热针 or 耳针 or 头皮针 or 头针
	4	#1 AND #2 AND #3
VIP	1	术后 or 手术 or 切除
	2	胃瘫综合征 or 胃瘫 or 胃轻瘫 or 胃排空延迟 or 胃排空障碍 or 胃排空无力 or 胃无力 or 胃麻痹 or 胃动力障碍
	3	针灸 or 针刺 or 针法 or 刺法 or 穴位 or 温针灸 or 温针 or 电针 or 腹针 or 浮针 or 揿针 or 皮内针 or 埋针 or 火针 or 毫针 or 热针 or 耳针 or 头皮针 or 头针
	4	#1 AND #2 AND #3
CBM	1	术后 or 手术 or 切除
	2	胃瘫综合征 or 胃瘫 or 胃轻瘫 or 胃排空延迟 or 胃排空障碍 or 胃排空无力 or 胃无力 or 胃麻痹 or 胃动力障碍
	3	主题词："针刺 + 针刺疗法"[不加权:扩展] + 自由词 19 个
	4	#1 AND #2 AND #3
ICTRP	1	acupuncture* OR (acupuncture therapy) OR acupoint* OR electroacupuncture* OR (electric acupuncture*) OR auriculotherapy OR (auricular acupuncture*) OR (elongated needl*) OR (scalp acupuncture*) OR (hand acupuncture*) OR (manual acupuncture*) OR (body acupuncture*) OR (warm* acupuncture*) OR (warm* needl*) OR (fire needl*) OR (trigger point*) OR meridian* OR needl* OR (abdominal acupuncture*) OR (float* needl*) OR (embedded acupuncture*) OR (intradermal acupuncture*) OR electro-acupuncture* OR micro-puncture* OR (needl* embedding) OR (dry needl*)
	2	gastroparesis OR gastropareses OR gastric stasis OR gastric stases OR (gastr* paralysis) OR (delay* gastric emptying) OR (disturbance of gastric emptying) OR (weakness of gastric emptying) OR (weakness of stomach) OR (stomach palsy) OR (gastr* motility disorder) OR (gastroparesis syndrome) OR (impaired gastric accommodation) OR (stomach paresis)
	3	#1 AND #2
ClinicalTrials.gov	1	acupuncture* OR "acupuncture therapy" OR acupoint* OR electroacupuncture* OR "electric acupuncture*" OR auriculotherapy OR "auricular acupuncture*" OR "elongated needl*" OR "scalp acupuncture*" OR "hand acupuncture*" OR "manual acupuncture*"
	2	gastroparesis OR gastropareses OR gastric stasis OR gastric stases OR "gastr* paralysis" OR "delay* gastric emptying" OR "disturbance of gastric emptying" OR "weakness of gastric emptying" OR "weakness of stomach" OR "stomach palsy"
	3	#1 AND #2
ChiCTR	1	胃瘫综合征 or 胃瘫 or 胃轻瘫 or 胃排空延迟 or 胃排空障碍 or 胃排空无力 or 胃无力 or 胃麻痹 or 胃动力障碍

Supplemental Table 3 Basic characteristics of the included studies

Study ID	Study period	Age (years)	Sample size (M/F)	Surgical site (Ab/Th)	Intervention			Outcomes	Multicenter trial	Funding
					Control (mg)	Experiment	Course (days)			
Chen 2013	2009-2011	E:48-76<br>C:45-78	E:14/6<br>C:12/8	E:20/0<br>C:20/0	Mosa (15)	C; FN 30min qd	14	1)9)	No	Provincial
Chen 2016	2010-2015	E:60<br>C:58	E:15/10<br>C:14/11	E:22/3<br>C:22/3	Prokinetic	C; FN, EA, WNM 30min bid	14	1)2)3)4)9)	No	NA
Dong 2017	2010-2017	E:59.12<br>C:60.03	E:13/18<br>C:15/16	E:31/0<br>C:31/0	Meto (20)	C; FN, EA, WNM 30min bid	N/A	1)2)3)4)6)7)8)11)12)13)	No	Provincial
Huang 2020	2018-2019	E:56<br>C:56	E:36<br>C:35	E:36/0<br>C:35/0	Meto (20)	C; FN 1min bid	7	1)3)4)6)	No	NA
Li 2005	N/A	E:54<br>C:50	E:37<br>C:30	E:37/0<br>C:30/0	Cisa (30)	C; FN, EA 20min qd	7	5)	No	NA

For peer review only

Lin 2017	2016	E:60.50<br>C:61.00	E:27<br>C:29	E:30/0<br>C:28/2	Meto (10)	C; FN 30min qd	Until cured	3)5)6)9)	No	NA
Liu 2017	2008-2012	E:37.2<br>C:38.1	E:15/11<br>C:13/7	E:26/0<br>C:20/0	Cisa (30), EM (500)	C; FN 25min qd	20	1)2)	No	NA
Ma 2014	2007-2012	35-80	E:29<br>C:29	E:29/0<br>C:29/0	Meto (20), Mosa (30-60), EM, Seda	C; FN 30min qd	N/A	7)	No	NA
Meng 2012	2006-2011	E:56<br>C:55	E:14/10<br>C:8/4	E:24/0<br>C:12/0	Meto, Cisa, EM	C; FN, EA 20min qd	14	1)2)5)	Yes	NA
Sun 2010	2007-	E:61.3<br>C:59.9	E:11/7<br>C:12/6	E:0/18<br>C:0/18	Mosa (15)	C; FN 20min qd	N/A	1)	No	NA
Wang 2013	2010-2012	44.37	E:11<br>C:11	E:11/0<br>C:11/0	Prokinetic	C; FN, EA, WNM 30min qd	21	1)9)	No	NA

For peer review only

Yang 2010	2007-2009	E:60.66<br>C:63.7	E:11/8<br>C:11/7	E:19/0<br>C:18/0	Meto (40)	C; FN, EA 20min qd-bid	28	1)2)7)	No	NA
Zhang 2021	2019-2020	E:46.78<br>C:46.25	E:0/33<br>C:0/32	E:34/0<br>C:34/0	Mosa (15)	C; FN qd	7	1)2)3)4)6)7)8)	No	Provincial
Zhang 2022	2018-2020	58.58	E:14/12<br>C:13/9	E:26/0<br>C:22/0	Mosa (15), Dom (30)	C; FN 30min qd	14	1)2)5)6)9)	No	NA
Zhao 2021	2017-2019	E:62.6<br>C:62.5	E:15/18<br>C:17/15	E:28/5<br>C:25/7	Cisa (30)	C; FN, WNM 30min qd	14	1)2)5)6)10)	No	Provincial

E, experiment group; C, control group; M, male; F, female; Ab, abdominal surgery; Th, thoracic surgery; Cisa: Cisapride; EM, Erythromycin; Meto, Metoclopramide; Mosa, Mosapride; Seda, Sedative; Dom, Domperidone; FN, filiform needle; EA, electro-acupuncture; WNM, warming needle moxibustion; 1), total effective rate; 2), recovery rate; 3), motilin; 4), gastrin; 5), gastric juice volume; 6), days of gastrointestinal motility recovery; 7), days of gastrointestinal decompression; 8), days of resume diet for the first time; 9), clinical symptom score; 10), adverse events; 11), albumin; 12), hemoglobin; 13), transferrin.

For peer review only

Supplemental Table 4 Evidence quality of each study (GRADE)

Outcome	Quality assessment							Relative effect (95% CI)	Absolute effect	Quality
	No. of studies	Design	Risk of bias	Inconsistency	Indirectness	Imprecision	Other considerations			
Total effective rate	12	randomised trials	serious	no serious inconsistency	no serious indirectness	no serious imprecision	none	RR 1.23 (1.14 to 1.33)	-	⊕⊕⊕O MODERATE
Recovery rate	8	randomised trials	serious	no serious inconsistency	no serious indirectness	no serious imprecision	none	RR 1.55 (1.29 to 1.86)	-	⊕⊕⊕O MODERATE
Motilin	5	randomised trials	serious	serious	no serious indirectness	no serious imprecision	none	-	MD 68.15 higher (42.89 to 93.40 higher)	⊕⊕OO LOW
Gastrin	4	randomised trials	serious	no serious inconsistency	no serious indirectness	no serious imprecision	none	-	MD 40.28 higher (30.86 to 49.70 higher)	⊕⊕⊕O MODERATE
Gastric juice volume	5	randomised trials	very serious	no serious inconsistency	no serious indirectness	no serious imprecision	none	-	MD 202.95 lower (216.96 to 170.2 lower)	⊕⊕OO LOW
Days to gastrointestinal motility recovery	6	randomised trials	very serious	serious	no serious indirectness	no serious indirectness	none	-	MD 4.87 lower (7 to 1.94 lower)	⊕OOO VERY LOW

For peer review only

Days to gastrointestinal decompression	4	randomised trials	serious	serious	no serious indirectness	no serious indirectness	none	-	MD 3.47 lower (5.74 to 1.21 lower)	⊕⊕OO LOW
Days to resumption of diet for the first time	2	randomised trials	no serious indirectness	serious	no serious indirectness	serious	none	-	MD 2.68 lower (4.23 to 1.12 lower)	⊕⊕OO LOW
Clinical symptom score	3	randomised trials	very serious	serious	no serious indirectness	serious	none	-	MD 1.31 lower (3.24 lower to 0.61 higher)	⊕OOO VERY LOW

For peer review only

Supplemental Table 5 Sensitivity analysis

	Total effective rate	Recovery rate	Motilin	Gastrin	Gastric juice volume	Days to gastrointestinal motility recovery	Days to gastrointestinal decompression	Days to resumption of diet for the first time	Clinical symptom score
Random	1.18 (95% CI 1.09, 1.27)	1.46 (95% CI 1.22, 1.74)	68.15 (95% CI 42.89, 93.40)	40.28 (95% CI 30.86, 49.70)	-193.58 (95% CI -216.96, -170.20)	-4.87 (95% CI -7.80, -1.94)	-3.47 (95% CI -5.74, -1.21)	-2.68 (95% CI -4.23, -1.12)	-1.31 (95% CI -3.24, 0.61)
Fixed	1.23 (95% CI 1.14, 1.33)	1.55 (95% CI 1.29, 1.86)	60.38 (95% CI 51.05, 69.71)	38.16 (95% CI 32.59, 43.73)	-193.58 (95% CI -216.96, -170.20)	-5.41 (95% CI -5.88, -4.94)	-2.68 (95% CI -3.13, -2.23)	-2.87 (95% CI -3.56, -2.18)	-0.85 (95% CI -0.93, -0.76)

Supplemental Table 6 Egger's test

	Std_Eff	Coef.	Std. Err.	t	P>|t|	[95% Conf. Interval]	
total effective rate	slope	0.8623299	0.978937	0.88	0.399	-1.318878	3.043537
	bias	0.6521827	0.886963	0.74	0.479	-1.324094	2.628459

For peer review only

Supplemental Figures

A

Study or Subgroup	Experimental Events	Experimental Total	Control Events	Control Total	Weight	Risk Ratio M-H, Fixed, 95% CI
**1.2.1 course⩽14 days**						
Chen 2013	17	20	11	20	5.1%	1.55 [1.00, 2.39]
Chen 2016	25	25	23	25	10.9%	1.09 [0.95, 1.24]
Huang 2020	35	36	32	35	15.0%	1.06 [0.95, 1.19]
Meng 2012	24	24	11	12	7.0%	1.11 [0.90, 1.36]
Zhang 2021	30	33	24	32	11.3%	1.21 [0.97, 1.52]
Zhang 2022	24	26	16	22	8.0%	1.27 [0.96, 1.68]
Zhao 2021	28	33	20	32	9.4%	1.36 [1.00, 1.84]
**Subtotal (95% CI)**		**197**		**178**	**66.8%**	**1.20 [1.10, 1.31]**
Total events	183		137			
Heterogeneity: Chi² = 8.95, df = 6 (P = 0.18); I² = 33%						
Test for overall effect: Z = 4.07 (P < 0.0001)						
**1.2.2 course＞14 days**						
Liu 2017	24	26	15	20	7.9%	1.23 [0.93, 1.62]
Wang 2013	10	11	5	11	2.3%	2.00 [1.02, 3.92]
Yang 2010	15	19	11	18	5.2%	1.29 [0.84, 2.00]
**Subtotal (95% CI)**		**56**		**49**	**15.4%**	**1.37 [1.08, 1.72]**
Total events	49		31			
Heterogeneity: Chi² = 1.85, df = 2 (P = 0.40); I² = 0%						
Test for overall effect: Z = 2.65 (P = 0.008)						
**1.2.3 course unclear**						
Dong 2017	29	31	23	31	10.7%	1.26 [1.00, 1.58]
Sun 2010	18	18	15	18	7.2%	1.19 [0.95, 1.50]
**Subtotal (95% CI)**		**49**		**49**	**17.8%**	**1.23 [1.05, 1.45]**
Total events	47		38			
Heterogeneity: Chi² = 0.12, df = 1 (P = 0.73); I² = 0%						
Test for overall effect: Z = 2.51 (P = 0.01)						
**Total (95% CI)**		**302**		**276**	**100.0%**	**1.23 [1.14, 1.33]**
Total events	279		206			
Heterogeneity: Chi² = 14.20, df = 11 (P = 0.22); I² = 23%						
Test for overall effect: Z = 5.46 (P < 0.00001)						
Test for subgroup differences: Chi² = 1.09, df = 2 (P = 0.58), I² = 0%						

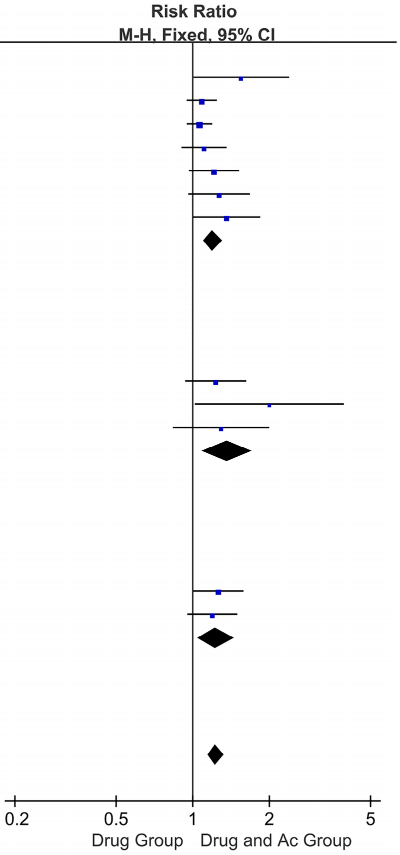

B

Study or Subgroup	Experimental Events	Experimental Total	Control Events	Control Total	Weight	Risk Ratio M-H, Fixed, 95% CI
**1.3.1 Drug and MAc**						
Chen 2013	17	20	11	20	5.1%	1.55 [1.00, 2.39]
Huang 2020	35	36	32	35	15.0%	1.06 [0.95, 1.19]
Sun 2010	18	18	15	18	7.2%	1.19 [0.95, 1.50]
Zhang 2021	30	33	24	32	11.3%	1.21 [0.97, 1.52]
Zhang 2022	24	26	16	22	8.0%	1.27 [0.96, 1.68]
**Subtotal (95% CI)**		**133**		**127**	**46.6%**	**1.21 [1.09, 1.34]**
Total events	124		98			
Heterogeneity: Chi² = 6.01, df = 4 (P = 0.20); I² = 33%						
Test for overall effect: Z = 3.56 (P = 0.0004)						
**1.3.2 Drug and SMAc**						
Chen 2016	25	25	23	25	10.9%	1.09 [0.95, 1.24]
Dong 2017	29	31	23	31	10.7%	1.26 [1.00, 1.58]
Liu 2017	24	26	15	20	7.9%	1.23 [0.93, 1.62]
Meng 2012	24	24	11	12	7.0%	1.11 [0.90, 1.36]
Wang 2013	10	11	5	11	2.3%	2.00 [1.02, 3.92]
Yang 2010	15	19	11	18	5.2%	1.29 [0.84, 2.00]
Zhao 2021	28	33	20	32	9.4%	1.36 [1.00, 1.84]
**Subtotal (95% CI)**		**169**		**149**	**53.4%**	**1.25 [1.13, 1.39]**
Total events	155		108			
Heterogeneity: Chi² = 7.80, df = 6 (P = 0.25); I² = 23%						
Test for overall effect: Z = 4.14 (P < 0.0001)						
**Total (95% CI)**		**302**		**276**	**100.0%**	**1.23 [1.14, 1.33]**
Total events	279		206			
Heterogeneity: Chi² = 14.20, df = 11 (P = 0.22); I² = 23%						
Test for overall effect: Z = 5.46 (P < 0.00001)						
Test for subgroup differences: Chi² = 0.23, df = 1 (P = 0.63), I² = 0%						

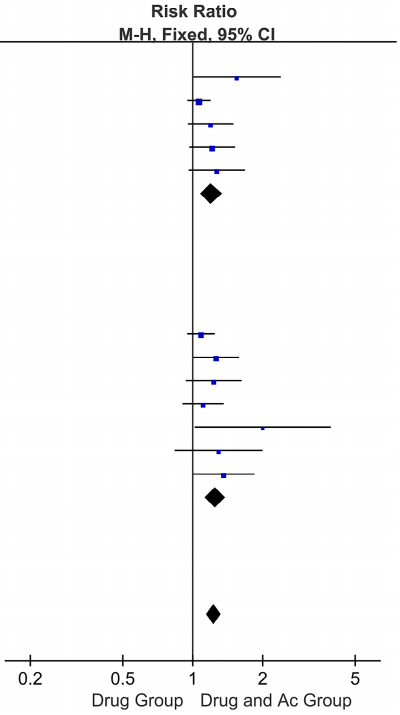

Supplemental Figure 1 Forest plots for subgroup analysis of acupuncture course(A) and acupuncture method(B) for the total effective rate.

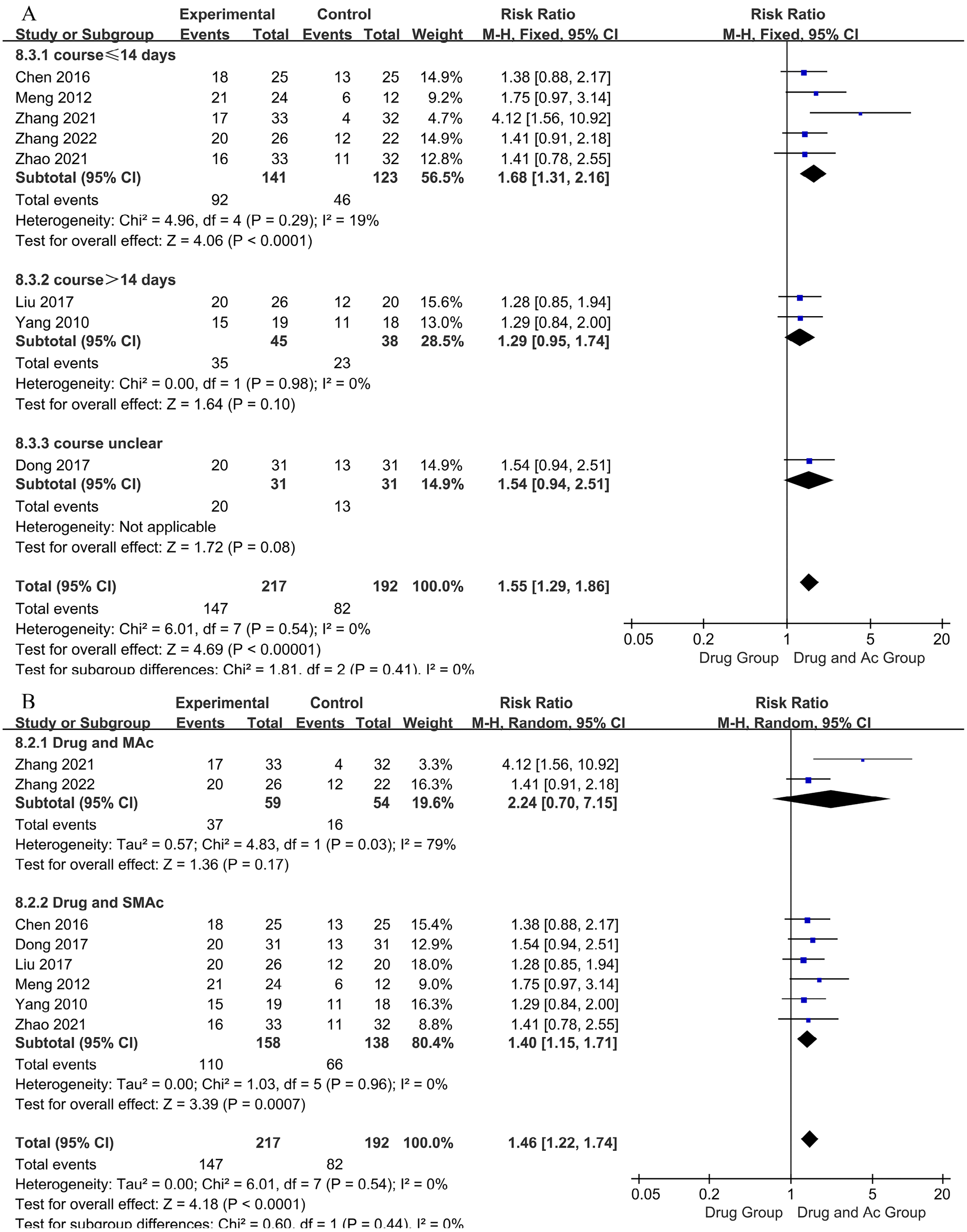

Supplemental Figure 2 Forest plots for subgroup analysis of acupuncture course(A) and acupuncture method(B) for recovery rate.

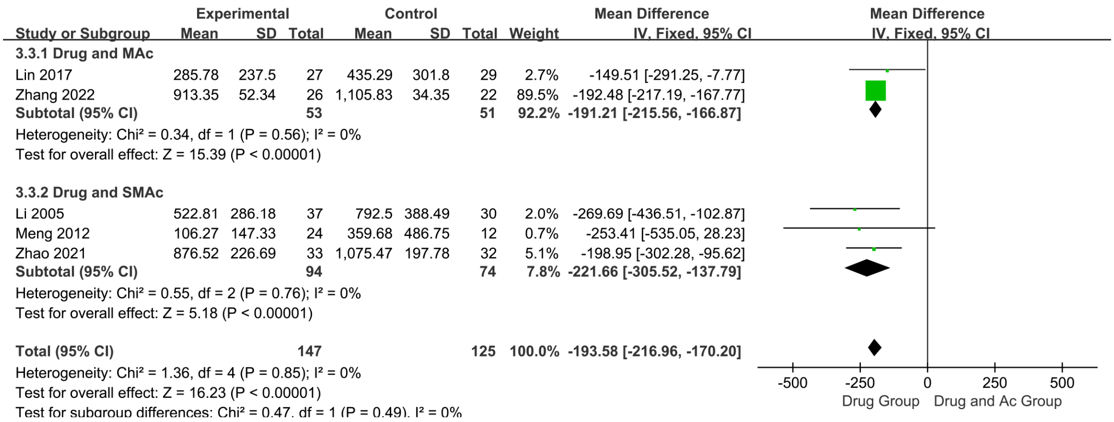

Supplemental Figure 3 Forest plot for subgroup analysis of acupuncture method for gastric juice volume.

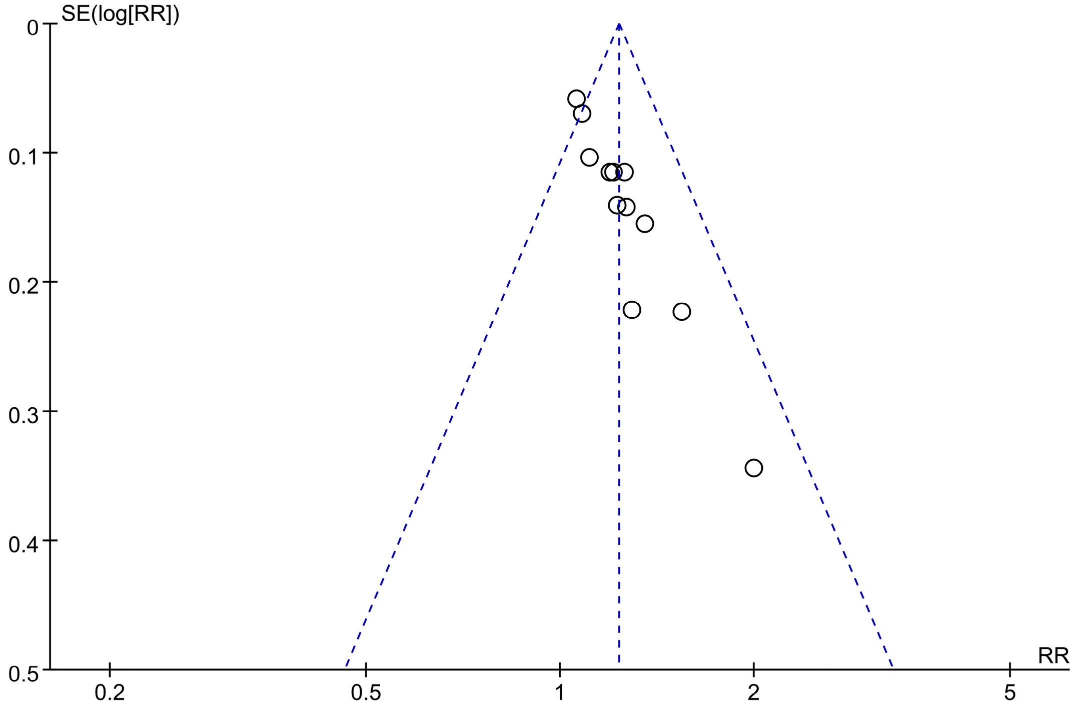

Supplemental Figure 4 Funnel plot of the total effective rate.

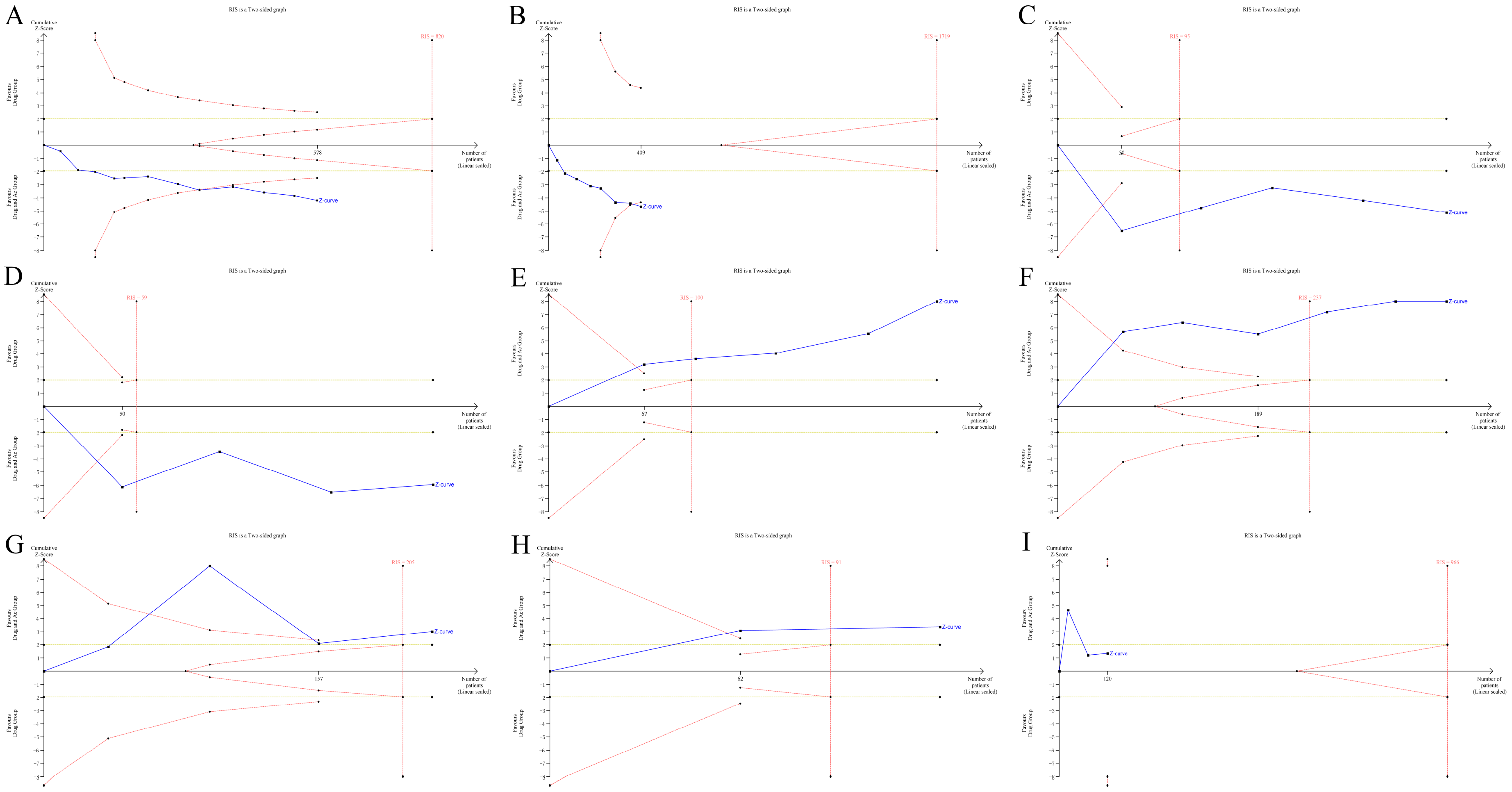

Supplemental Figure 5 TSA for (A)TER, (B)RCR, (C)MTL, (D)GSA, (E)GJV, (F)GMR, (G)GD, (H)RDT, (I)CSS. The continuous blue line represents the Z line (cumulative effect size), red dashed lines represent the trial sequential monitoring boundary and RIS, and the yellow dashed

lines represent the conventional test boundary.

For peer review only
